# Saving the Mahachai Betta: Genetic Erosion and Conservation Priorities Under Urbanization Pressure

**DOI:** 10.3390/ani15192820

**Published:** 2025-09-26

**Authors:** Ton Huu Duc Nguyen, Trifan Budi, Tavun Pongsanarm, Thitipong Panthum, Worapong Singchat, Narongrit Muangmai, Aingorn Chaiyes, Warong Suksavate, Sahabhop Dokkaew, Darren K. Griffin, Prateep Duengkae, Kornsorn Srikulnath

**Affiliations:** 1 Animal Genomics and Bioresource Research Unit (AGB Research Unit), Faculty of Science, Kasetsart University, 50 Ngamwongwan, Chatuchak, Bangkok 10900, Thailand; nguyenhuuduc.t@ku.th (T.H.D.N.); trifanbudi26@gmail.com (T.B.); tavan.p@ku.th (T.P.); thitipong.pa@ku.th (T.P.); worapong.singc@ku.ac.th (W.S.); aingorn.ch@ku.ac.th (A.C.); fforwos@ku.ac.th (W.S.); d.k.griffin@kent.ac.uk (D.K.G.); prateep.du@ku.ac.th (P.D.); 2 Interdisciplinary Graduate Program in Bioscience, Faculty of Science, Kasetsart University, 50 Ngamwongwan, Chatuchak, Bangkok 10900, Thailand; 3 Faculty of Biology Education, School of Education, Can Tho University, 3/2 Street, Ninh Kieu Ward, Can Tho 900000, Vietnam; 4 School of Agricultural Technology, King Mongkut’s Institute of Technology Ladkrabang, Bangkok 10520, Thailand; 5 Special Research Unit for Wildlife Genomics (SRUWG), Department of Forest Biology, Faculty of Forestry, Kasetsart University, 50 Ngamwongwan, Chatuchak, Bangkok 10900, Thailand; 6 Department of Fishery Biology, Faculty of Fisheries, Kasetsart University, Bangkok 10900, Thailand; ffisnrm@ku.ac.th; 7 The International Undergraduate Program in Bioscience and Technology, Faculty of Science, Kasetsart University, Bangkok 10900, Thailand; 8 Department of Aquaculture, Faculty of Fisheries, Kasetsart University, Bangkok 10900, Thailand; ffisspd@ku.ac.th; 9 School of Natural Sciences, University of Kent, Canterbury, Kent CT2 7NJ, UK; 10 Center for Advanced Studies in Tropical Natural Resources, National Research University-Kasetsart University, Kasetsart University, Bangkok 10900, Thailand; 11 Biodiversity Center Kasetsart University (BDCKU), Faculty of Science, Kasetsart University, Bangkok 10900, Thailand

**Keywords:** conservation, genetic diversity, salinity, fighting fish

## Abstract

Background/Objectives: Mahachai Betta (*Betta mahachaiensis*) is a bubble-nesting fighting fish endemic to brackish habitats in Bangkok, Samut Sakhon, and Samut Prakan, where rapid urbanization and industrial growth threaten persistence. We evaluated genetic structure and diversity across 10 populations (81 individuals) to inform conservation planning. Methods: This study combined microsatellite genotyping (13 loci) with ecological niche modeling to assess genetic variability, population connectivity, and landscape–environmental drivers of differentiation. Results: Habitat loss and fragmentation were associated with reduced gene flow and decreased genetic diversity. Mean allelic richness was 2.65 and expected heterozygosity ranged from 0.20 to 0.46, with *F*_ST_ values up to 0.400. Forward simulations predicted severe erosion of diversity within the next 12.5–37.5 years. Populations showed clear genetic subdivision, most pronounced in Samut Prakan and Samut Sakhon, with two Samut Sakhon populations (SKN3 and SKN7) reflecting strong environmental heterogeneity. Conclusions: Improving habitat connectivity and intensifying local community engagement are priority actions to enhance the resilience and long-term persistence of Mahachai Betta. This study provides the first integrated genetic and landscape-based assessment of the species, highlighting its rapid genetic erosion under urbanization and offering a foundation for targeted, evidence-based conservation strategies.

## Simple Summary

The Mahachai Betta is a rare fighting fish that lives only in brackish canals and ponds around Bangkok and nearby provinces. These habitats are being rapidly lost because of urban growth, pollution, and changes in water quality. To understand how this affects the species, we studied ten populations and analyzed their genetic diversity. The results showed that many groups of Mahachai Betta have very low genetic variation and are becoming increasingly isolated from each other. Computer simulations further suggest that the loss of diversity will continue rapidly in the coming decades if no action is taken. This means that the fish will become less able to adapt to environmental changes and more at risk of extinction. Our findings highlight the urgent need to protect and connect the remaining habitats and to involve local communities in conservation. By safeguarding both the environment and the genetic diversity of this unique fish, we can improve its chances of survival for future generations.

## 1. Introduction

Fighting fish, or bettas (*Betta* spp.) are native to Southeast Asia and prized in the aquarium trade for their fighting and ornamental characteristics. Mahachai Betta (*Betta mahachaiensis*) is distinguished by ornamental males with iridescent green-blue stripes on a black-brown background and is restricted to Samut Sakhon and Samut Prakan provinces in central Thailand. These fish inhabit tidal brackish waters with Nipa Palms (*Nypa fruticans*), where they establish territories, build bubble nests, and spawn [1,2,3,4]. Being the only wild *Betta* species surviving in such a harsh environment, their distribution illustrates the hypothetical model of parapatric speciation [5]. However, the species is likely endangered due to specific water chemistry requirements, restricted habitat range, and accelerating human activities such as urbanization and industrial development.

Habitat loss, which involves the reduction in forests, grasslands, deserts, and wetlands, impacts biodiversity and ecological functions and is recognized as a major environmental issue worldwide for many species [6]. Habitat loss is accelerated by rapid urbanization, which reduces species composition and population sizes globally [7,8]. Rapid urbanization has occurred particularly in Thailand’s central coastal regions, including the Samut Sakhon, Samut Prakan, and Bangkok provinces. Here, urbanization rates have increased from 46.19% in 2013 to 56.61% in 2023 [9]. As a primary economic driver that contributed 39.79% to the nation’s GDP in 2022, exponential population growth was observed in the three provinces, where the population increased from 2.2 million people in the 1960s to over 12.8 million people in 2023 [9]. Severe environmental pressures, including land subsidence, saltwater intrusion and water pollution, are exacerbated by rapid development and excessive groundwater extraction [10,11,12,13]. The adaptation of Mahachai Betta near urban areas is thus hampered, and the species faces inbreeding risks. Furthermore, habitat loss due to urban and industrial growth increases extinction risk [14]. To protect this species, natural habitats must be conserved and conservation measures must be implemented in order to mitigate against the effects of urbanization in central coastal Thailand [15,16].

In conservation biology, species distribution and habitat extinction risks are assessed to improve survival in connected areas [17]. The environmental dependence of a species must be understood, and richness patterns must be clarified to reveal the range limitations and causes of gene flow. Remarkably, ecological theories have been studied using species from diverse landscapes; the Mahachai Betta reflects the dynamics amid Thailand’s coastal development [1]. In this context, it is important to quantify how variations in ecological factors that are linked to habitat quality affect and shape genetic variability in wild populations. The effects of habitat loss and fragmentation on genetic structure and variability have been extensively validated through empirical evidence and formalized in theoretical frameworks [18]. Identifying habitat suitability is essential for delineating species distribution boundaries and conservation zones.

To investigate their genetic variation, Mahachai Betta across 10 populations in 3 central Thai provinces were analyzed using microsatellite genotyping and comprehensive population genetic and landscape analyses. The correlation between genetic and environmental features was examined using ecological niche models. Two hypotheses pertaining to central Thailand’s developing regions have been proposed in this context as follows: (1) genetic diversity in Mahachai Betta is influenced by landscape and environmental factors; (2) population structure differences are caused by geographic isolation. In this study, the population status of Mahachai Betta was thus investigated as an ecological bioindicator to provide insights that will aid in the development of effective conservation and management plans.

## 2. Materials and Methods

### 2.1. Specimen Collection and DNA Extraction

**Figure 1:**
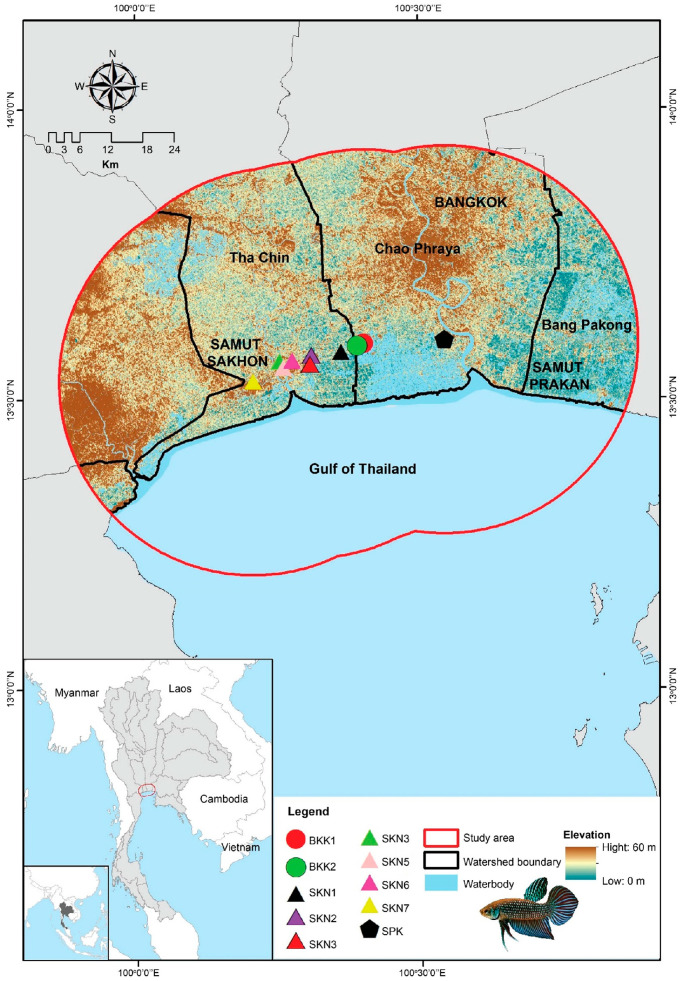
Geographic distribution of sampling sites (red dots) across the regions of Bangkok, Samut Sakhon, and Samut Prakan in Thailand.

Mahachai Betta were collected from three provinces in central Thailand: Bangkok, Samut Prakan, and Samut Sakhon. Eighty-one individuals were collected from 10 populations, including those that belong to the Chao Phraya River watershed with the Bang Pla Kot Canal (SPK) (one population), Sanam Chai Canal (BKK1 and BKK2) sub-watersheds (two populations), and the Tha Chin River watershed with the Maha Chai Canal (SKN1–SKN3), Khru Canal (SKN4–SKN6), and Sunak Hon Canal (SKN7) sub-watersheds (seven populations) (Figure 1). Water quality parameters (pH, dissolved oxygen [DO], conductivity, and salinity) were measured in situ and analyzed using APHA methods [19]. The Kruskal–Wallis test was used to compare water quality parameters between populations using R version 4.1.2 [20]. Detailed information on the samples is provided in Table S1. The Mahachai Betta specimens were identified morphologically using prior descriptions Kowasupat et al. [1]. All animal care and experimental procedures were approved by the Kasetsart University Animal Experiment Committee (approval numbers ACKU65-SCI-026 and ACKU01157) and conducted in accordance with the Regulations of Animal Experiments at Kasetsart University and ARRIVE guidelines (https://arriveguidelines.org, accessed on 30 November 2024). Caudal fin clips from each fish specimen were collected and preserved in 95% ethanol. Genomic DNA was isolated based on the salting-out protocol described by Supikamolseni et al. [21], with slight modifications. DNA quality and concentration were assessed as described by Wattanadilokchatkun et al. [22].

### 2.2. Microsatellite Genotyping and Data Analysis

Thirteen microsatellite loci were used based on a previous study on Siamese fighting fish (*Betta splendens*) [23,24]. Detailed information on the primers used is provided in Table S2. The 5’-end of each forward primer was labeled with either Fluorescein Amidite (6-FAM) or Hexachloro-fluorescein (HEX) fluorescent dye (Macrogen, Seoul, Republic of Korea). PCR amplification, product visualization, and microsatellite genotyping were performed as described by Wattanadilokchatkun et al. [22]. Genotyping data from the 10 Mahachai Betta populations were used for the analysis. Genetic diversity parameters, including allelic richness (*AR*), average number of alleles per locus (*N*_a_), number of effective alleles (*N*_e_), Shannon’s information index (*I*), heterozygosity (*H*_o_ and *H*_e_), *F*-statistics (*F*_IS_ and *F*_ST_), polymorphic information content (*PIC*), relatedness (*r*), and *M* ratio were calculated as previously described by Wattanadilokchatkun et al. [22]. Deviations from Hardy–Weinberg equilibrium and linkage disequilibrium were assessed using Arlequin software version 3.5.2.2 [25]. Significant differences in heterozygosity within and among populations, distribution of pairwise *r*-values, overall *F*_IS_, and recent bottleneck event investigations based on the single mutation model (SMM) and two-phase mutation (TPM) were conducted following Wattanadilokchatkun et al. [22]. The selective sweep was investigated by plotting *H*_e_ and *F*_IS_ values at each microsatellite locus, as described by Budi et al. [26]. Neutral or balanced selection is reflected by a low *F*_IS_ and high *H*_e_, whereas a high *F*_IS_ and low *H*_e_ indicate a selective sweep or purifying selection [27]. Microsatellite locus neutrality was tested using a Bayesian regression approach implemented in BAYESCAN [28], which estimates the probability of a locus being under selection by calculating the Bayes factor, which represents the ratio of the posterior probabilities of selection versus neutral models based on the data.

### 2.3. Population Genetic Structure and Demography Analyses

Principal Coordinate Analysis (PcoA), Discriminant Analysis of Principal Components (DAPC), and STRUCTURE analyses were performed as described by Budi et al. [26]. Forward simulations to simulate future genetic changes were performed as described by Wongloet et al. [29]. Five scenarios related to carrying capacity were incorporated in this study: 50% reduced population size, current population size, 50% increase in population size, doubled population size, and 150% increase in population size. Each simulation was conducted over 250 generations with a substitution rate of 3.5% per million years [30]. Recent migration rates were assessed using BayesAss version 3.0.5 [31], and historical gene flow was calculated in MIGRATE-N version 3.6.11 [32], following the settings of Patta et al. [33]. Landscape shape interpolation (LSI) analysis was performed using Alleles in Space version 1.0 [34] to visualize the spatial patterns of genetic diversity as previously outlined by Wattanadilokchatkun et al. [22]. The model that best explained the statistical origin of the reintroduced population was determined using DIYABC software (version 2.1.0). A Bayesian computational approach (ABC) was used to evaluate the posterior probabilities of past scenarios [35].

### 2.4. Analysis of Landscape Using Occurrence and Climate Data

As mentioned above, data on Mahachai Betta in Thailand are considered scarce, with only 10 known localities identified. Ten occurrence records were randomly divided at a 70:30 ratio into training and testing data from the field observation dataset. Coarse-scale environmental dimensions are commonly used as a proxy for a species’ fundamental niche, i.e., the environmental conditions under which the species can survive and maintain populations in the long term without the need for immigration [36]. Fifteen bioclimatic variables from the WorldClim version 2.1 online database (https://www.worldclim.org/data/worldclim21.html, accessed on 30 November 2024) were used as the additional environmental data. Variables with artifactual values were excluded because of abrupt, unrealistic climatic changes observed between neighboring pixels (i.e., 8, 9, 18, and 19) [37]. These variables were interpolated to a global surface at a spatial resolution of 30 arcseconds (approximately 1 km^2^ cell size), spanning 1970–2000 [38]. Variable selection was examined using variance inflation factor (VIF) analysis [39], which is considered an effective method for selecting variables [40]. VIF was calculated by dividing the variance of a model that includes multiple predictors by the variance of models considering each predictor individually. This method helped identify the variance explained by each variable. Predictors with VIF values < 5 were selected as the most appropriate variables [41].

### 2.5. Model Calibration Area

The interactions among geographical access, abiotic conditions, and biotic environmental factors have been highlighted in the BAM framework [42,43]. When models were built, it was crucial to include the areas a species could reach, defined as M (movement) within the BAM framework [42]. The study sites assumed that this area formed the boundary of the regions where Mahachai Betta had historically dispersed. This calibration area was used to limit the occurrence of environmental layers and unique species, generating an ecological niche model representing the fundamental niche of Mahachai Betta. The study area was defined by buffering the occurrence points 37 km apart, thus restricting the geographical extent of the model (Figure 1). The total study area covered 4858 km^2^ (13°18′–13°56′ N, 99°52′–100°53′ E), and all environmental raster grids were clipped to this study area.

### 2.6. Ecological Niche Model

The fundamental niche model was developed using the ellipsoid model function from the “ellipse” package in R version 4.1.2 [20]. In Grinnellian terms, an ecological niche is referred to as a set of environmental conditions that enable a species to sustain populations over time without needing immigration [36]. Ellipsoid envelope models were generated using the “ellipse” package, assuming that a species’ ecological niche was convex, with a single optimal point, and that the species’ response to each variable was correlated with other variables [41,44]. Mahalanobis distances were used to measure the extent to which the environmental conditions were at the optimal point (ellipsoid centroid). The suitability values were generated by applying a multivariate normal transformation to these distances. Consequently, higher suitability values were found near the centroid, and lower values were found closer to the boundary of the ellipsoid. Suitability predictions were reclassified into binary layers: 0 (unsuitable) and 1 (suitable).

### 2.7. Investigating the Relationship Between Environmental Influences and Genetic Diversity

Spearman’s coefficient was used to evaluate the correlation between Mahachai Betta genetic diversity (*AR*, *H*_e_, and *F*_IS_) and habitat suitability, alongside landscape-level variables (pH, DO, conductivity, salinity, water temperature, annual mean temperature, precipitation from 1981 to 2010, and elevation; Table S3). The relationship between each landscape-level variable and the genetic diversity of Mahachai Betta was quantified. The linear association between genetic diversity and landscape-level variables was quantified using multiple regression analysis. The best model was identified using a stepwise selection procedure based on the Akaike information criterion [45]. Statistical significance was set at *p* < 0.05. R version 4.4.1 [20] was used for all statistical analyses.

## 3. Results

### 3.1. Water Quality of Mahachai Betta Population Habitats

pH was highest in the habitat of population SKN4 and lowest in that of population SKN5 (*p* < 0.05) (Figure S1). The highest DO was found in the habitat of SKN3 and the lowest in that of BKK1. Differences in conductivity and salinity were observed, with the highest values observed in the habitat of SKN3 and the lowest in that of SKN2. Across all populations, water quality parameters varied considerably, with pH ranging from 6.8 to 8.9, dissolved oxygen from 0.8 to 6.5 mg/L^−1^, salinity from 3 to 27 PSU, and conductivity from 5000 to 42,000 μS/cm^−1^.

### 3.2. Assessment of the Genetic Variability of Mahachai Betta Populations

In total, 81 individuals across 10 populations were genotyped, and 344 alleles were found across all loci. The average number of alleles per locus ranged from 1.288 to 4.219. Positive *F*-values were observed in six populations (SPK, BKK1, SKN2, SKN4, SKN5, and SKN7), whereas negative *F*-values were found in the remaining four (BKK2, SKN1, SKN3, and SKN6) (Table 1). The *PIC* values across populations ranged from 0.425 to 0.653. The *I* value varied from 0.346 to 0.841 across populations (Table 1 and Table S4). *H*_o_ and *H*_e_ values ranged from 0.238 to 0.462 and 0.201 to 0.464, respectively (Table 1 and Table S4). No significant difference was observed between *H*_o_ and *H*_e_ based on Welch’s *t*-test (Table 2). Similarly, Bartlett’s test indicated no significant differences between the two indices across populations (Table S5). The mean *AR* across all populations was 2.646 ± 0.149 (Table 1 and Table S4). Detailed genetic diversity data are presented in Table 1 and Table S4. Most allelic frequencies in the population deviated from what would be expected under Hardy–Weinberg equilibrium (Tables S6–S15). Null alleles were frequently observed at eight loci (BettaMS4, BettaMS15, BettaMS23, BettaMS25, BettaMS28, BettaMS40, BettaMS2.2, and BettaMS10.1), and all markers listed were treated similarly. The results from the 250 simulated generations indicated a decrease in both *AR* and *H*_e_ values. Notably, the decreasing rates of *AR* and *H*_e_ varied for each carrying capacity scenario and were lowest when the carrying capacity was highest. Very low levels, close to 1 for *AR* and 0 for *H*_e_, were reached after fewer than 50–150 generations, depending on the carrying capacity (Figure S2).

The mean pairwise relatedness *r*-values were computed for 461 combinations encompassing 81 sampled individuals. The mean *r*-values for the 10 populations ranged from −0.283 to −0.030 (Table S16). Most *r* pairs had results ranging from greater than −0.25 to less than 0.25, except for seven pairs with values lower than −0.25 (Tables S17–S26). A left-skewed distribution was observed in the *r*-values of the Mahachai Betta population, indicating that the pairwise *r*-values were lower than those expected by chance for unrelated individuals (Figure S3). The pairwise distribution of *r* significantly differed between the BKK2, SKN1, SKN2, SKN5, SKN6, and SKN7 populations. However, no significant differences were observed in the pairwise distribution of *r* among the remaining populations, namely, SPK, BKK1, SKN3, and SKN4 (Table S27). The *F*_IS_ values for the 10 populations ranged from −0.756 to −0.134 (Tables S28–S37). The distributions of *F*_IS_ in the SPK, BKK2, SKN1, SKN2, and SKN3 populations differed significantly (Table S27). A left-skewed distribution was observed in *F*_IS_ (Figure S3). The effective population sizes (*N*_e_) in SPK, SKN4, and SKN7 were 48.500 (95% CI: 12.300–13.000), 38.100 (95% CI: 7.400–infinite), and 0.900 (95% CI: 0.900–infinite), respectively. However, the remaining populations, including BKK1, BKK2, SKN1, SKN2, SKN3, SKN5, and SKN6, had infinite values (95% CI: Infinite) (Table S16).

**Figure 2:**
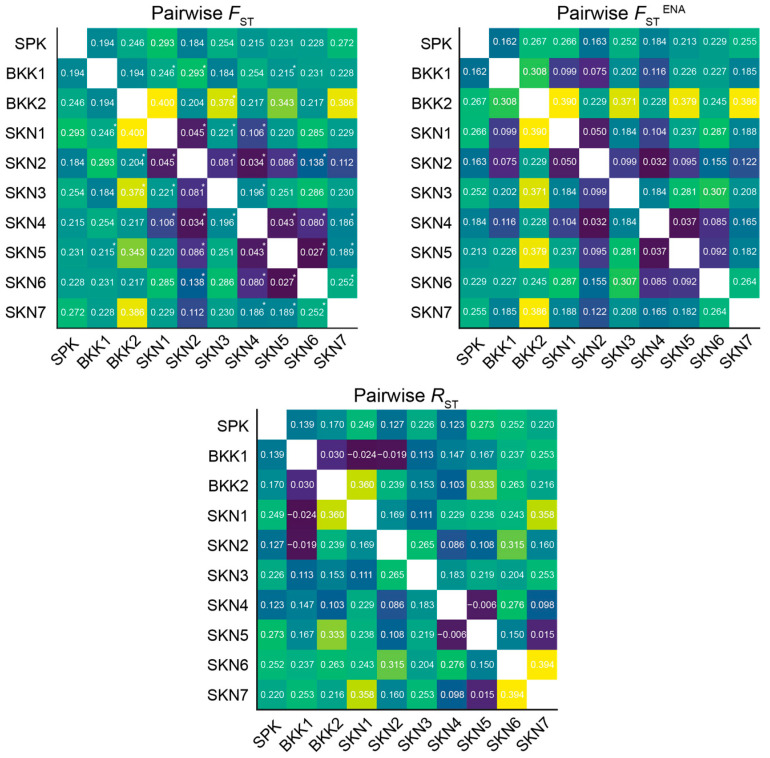
Pairwise genetic differentiation (*F*_ST_), pairwise *F*_ST_^ENA^ values with ENA correction for null alleles and *R*_ST_ values using FSTAT version 2.9.3 [47] of Mahachai betta (*Betta mahachaiensis*) based on 13 microsatellite loci. The number indicates *p* values, with 110 permutations. * = statistically significant difference.

After 110 permutations, the *F*_ST_ values across the 10 populations ranged from 0.027 to 0.400. Among the 45 combination pairs, 20 exhibited significant differences in *F*_ST_ estimates (*p* < 0.05) (Table S38). A similar pattern was observed when estimating *F*_ST_^ENA^ (allele frequency–corrected *F*_ST_ using the ENA method to reduce bias from null alleles) between populations, ranging from 0.032 to 0.390 (Figure 2). The analysis of molecular variance results for the Mahachai Betta populations indicated that 19% of the genetic variation was found within populations and 16% between populations (Table S38). The results of the *R*_ST_ of the 10 populations ranged from −0.024 to 0.394. The closest relationship between the populations, as indicated by the *R*_ST_ values, was between SKN6 and SKN7. By contrast, the most distant relationship was observed between BKK1 and SKN1 (Figure 2). Based on Nei’s genetic distances, the BKK2 and SKN5 populations showed the most distant genetic relationships, whereas the SKN2 and SKN4 populations had the closest genetic relationships (Table S39). Wilcoxon signed-rank tests for recent population bottlenecks were conducted using the SMM (with values ranging from 0.004 to 0.979) and TPM (ranging from 0.004 to 0.974) methods across all populations. Mode-shift tests indicated a “normal L-shaped distribution” for populations SPK, BKK1, SKN2, and SKN4, which suggested stability without recent bottlenecks (Table S40). By contrast, the remaining populations exhibited a “Shifted mode,” which implied that recent bottlenecks likely altered their allele frequency distributions. The *M* ratio in the populations ranged from 0.157 to 0.387, which suggested historic bottleneck events (<0.68) (Table 1) [46]. No evidence of genetically selective sweeps was observed (Figure S4).

### 3.3. Clustering, Gene Pool Profiling, and Gene Flow

**Figure 3:**
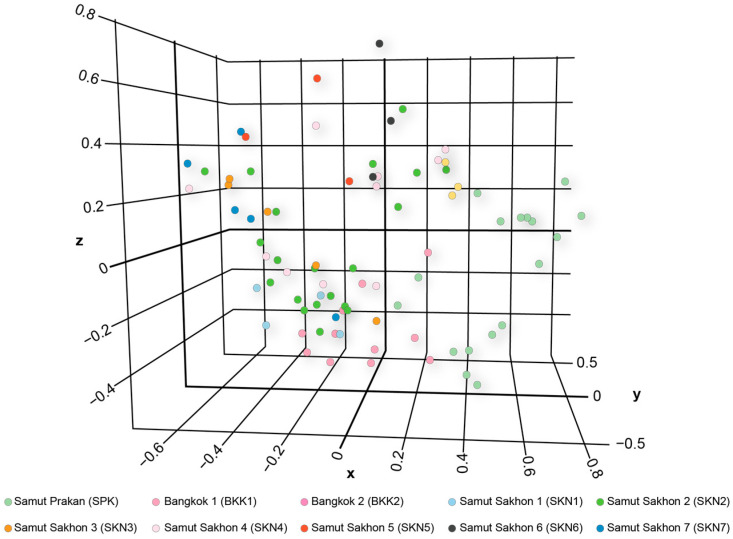
Principal Component Analysis (PCoA) for Mahachai Betta (*Betta mahachaiensis*) at Samut Prakan, Bangkok, and Samut Sakhon.

**Figure 4:**
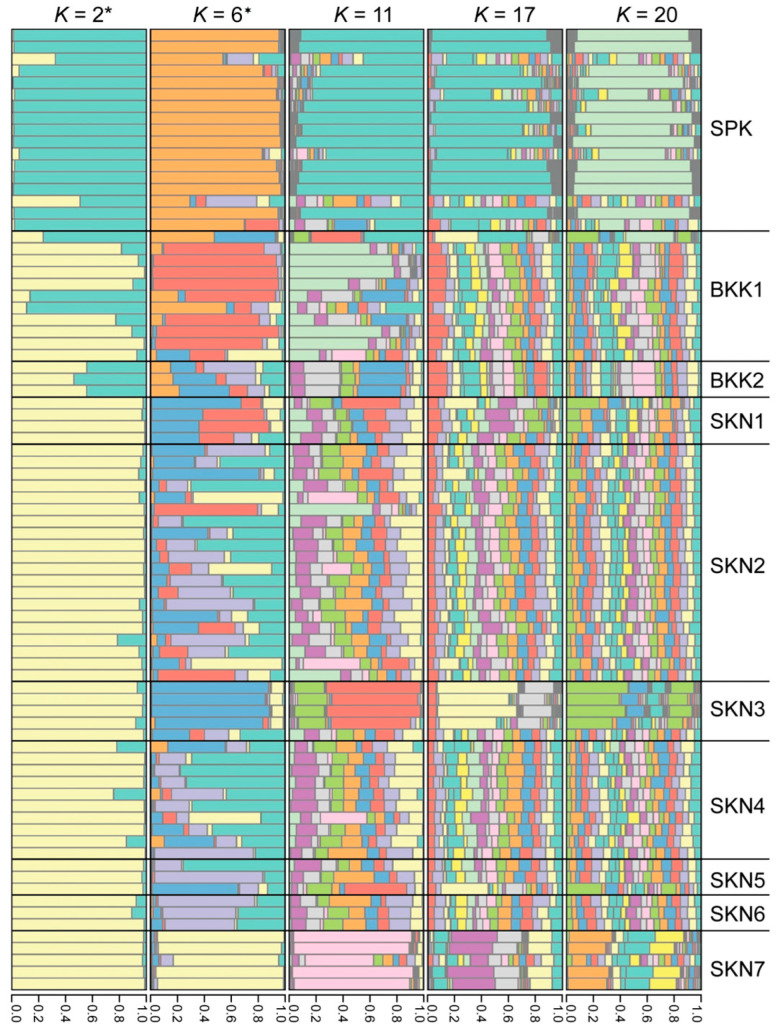
Population structure of the 10 populations of Mahachai Betta (*Betta mahachaiensis*). The *x*-axis represents the proportion of membership (posterior probability) in each genetic cluster, while each horizontal bar on the *y*-axis represents an individual. The most probable number of *K*-values is represented by an asterisk (*) and star (✶), according to Evanno’s Δ*K* and ln Pr (X|*K*) strategies, respectively. Each color represents a distinct genetic cluster inferred by STRUCTURE analysis.

The PCoA demonstrated that the first, second, and third principal components explained 13.58, 11.27, and 9.40% of the total variation, respectively (Figure 3). The PCoA and DAPC results revealed a distinct cluster in the SPK population compared with the other populations (Figure S5). The model-based Bayesian clustering algorithms implemented in STRUCTURE generated different population patterns, with *K*-value increasing from 2 to 20. Based on Evanno’s Δ*K*, the highest posterior probability was observed at *K* = 2 (Figure S6a); however, based on ln Pr (X|*K*), the optimized population structure patterns were assigned to six clusters (*K* = 6) (Figure S6b). At *K* = 2, a distinct pattern was observed in the SPK population, whereas similar patterns appeared in BKK1 and BKK2, and identical patterns were observed in SKN1–SKN7. At *K* = 6, a different pattern was observed in the SPK population than in the others. Diverse gene pools were observed in the BKK1, BKK2, SKN1, SKN2, SKN4, SKN5, and SKN6 populations, whereas SKN3 and SKN7 exhibited different genotypes. With an increased *K*-value, similar gene pool patterns were observed in the Mahachai Betta populations, except for SPK, SKN3, and SKN7 (Figure 4). Loci BetaMS14.2, BetaMS5, and BetaMS40 were identified using the BAYESCAN approach as having a high probability of being under directional selection (Figure S7). Of these alleles, 2 (194 and 202) at the BetaMS5 locus were found only in the SPK population, 3 (139, 141, and 143) at the BetaMS40 locus were found only in the SKN2 population, and 1 (220) at the BetaMS14.2 locus was found only in the BKK2 population.

**Figure 5:**
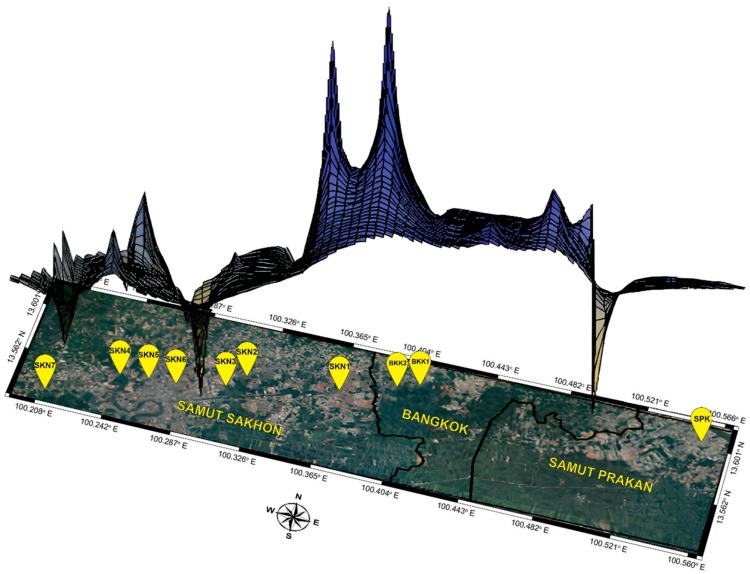
Results of genetic landscape shape interpolation analysis. x and y axes correspond to geographic locations within the populations analyzed in the study. Surface plot heights reflect genetic distance differentiation; high values (blue) denote areas with more significant genetic differentiation, while low values (yellow) denote areas with lower genetic differentiation, indicating more genetic similarity.

**Figure 6:**
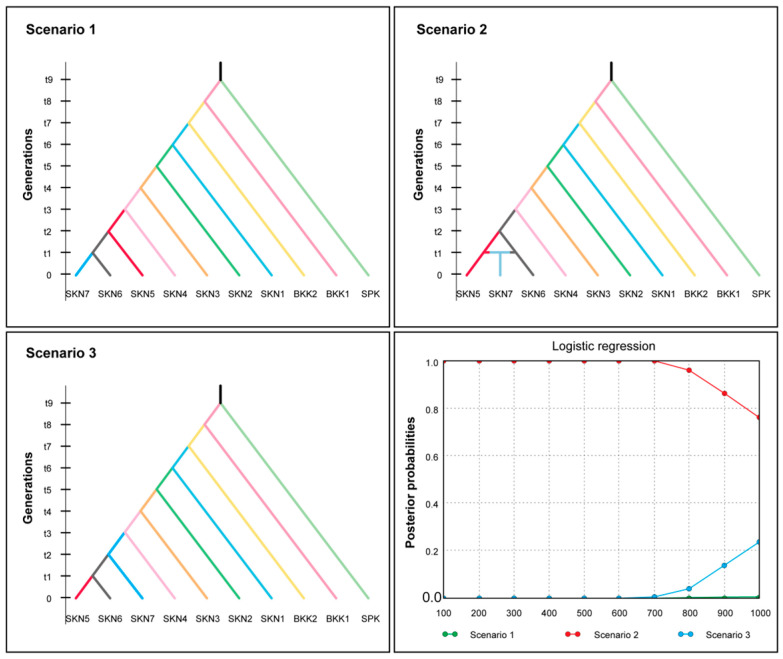
Assessment of population history scenarios for Mahachai Betta (*Betta mahachaiensis*) using approximate Bayesian calculation (ABC) inference.

The BAYESASS analysis revealed that gene flow varied from 0.689 to 0.890 within populations and from 0.011 to 0.135 between populations. The highest gene flow among the populations was 0.135 from SKN2 to SKN7. The lowest gene flow between populations was 0.011 from SPK to SKN2 (Table S41 and Figure S8). MIGRATE-N analysis showed a broad spectrum of mutation-scaled immigration rates (*M*) ranging from 5.667 to 979.000, with the highest value observed between SKN6 and SKN4. Variations in mutation-scaled population size (Θ) ranged from 0.000 to 0.099 across populations. The remaining populations (BKK1, BKK2, SKN3, and SKN7) displayed Θ values of 0.000 on the mutation-scaled scale (Table S42, Figure S8). The *N*_m_ values in the populations of this fish species ranged from 0.000 to 23.860, with the highest value of 23.860 observed in populations SKN1 to SKN5 (Table S43). IBD significantly correlated with population and geographical distance (*r* = 0.63, *p* < 0.01). Additionally, LSI analyses indicated the presence of genetically divergent areas in the 10 assessed populations. Low genetic differentiation in Mahachai Betta was observed in three main regions: the lower Chao Phraya River (SPK), the Maha Chai Canal (SKN3 and SKN6), and the lower Tha Chin River (SKN7) (Figure 5). ABC analysis was performed to determine the origin of the Mahachai Betta population (Figure 6). Scenario 2, with the highest posterior probability of 0.958, showed that the SKN7 population originated from SKN5 and SKN6. By contrast, Scenario 1, in which SKN7 diverged from SKN6, and Scenario 3, in which SKN7 diverged before the split between SKN5 and SKN6, exhibited very low posterior probabilities of 0.001 and 0.041, respectively.

### 3.4. Habitat Suitability of Mahachai Betta

**Figure 7:**
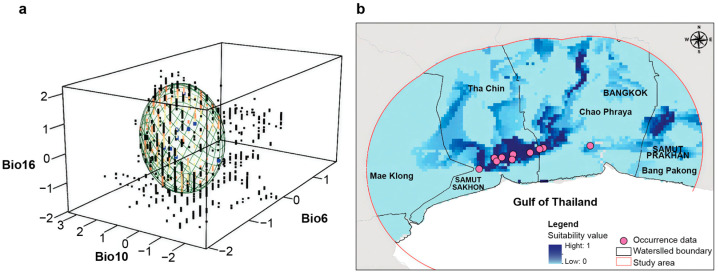
Environmental suitability and habitat maps of Mahachai Betta (*Betta mahachaiensis*). (**a**) Representation of suitability values in environmental space. Occurrence data is represented in blue. Cold colors represent low suitability, and warm colors indicate high suitability. (**b**) Habitat suitability maps of *B. mahachaiensis*. Suitability values range between 0 and 1.

Three bioclimatic variables were selected based on the available climate for Mahachai Betta: Bio6 (Min Temperature of Coldest Month; VIF = 0.164), Bio10 (Mean Temperature of Warmest Quarter; VIF = 0.264), and Bio16 (Precipitation of Wettest Quarter; VIF = 0.319) (Figure 7a). According to the Mahalanobis distances, the most suitable habitats in the Tha Chin watershed (covering SKN4 and SKN5) were mainly distributed at the junction of flat plains in the southern Tha Chin and Chao Phraya watersheds, with a few patches in the Chao Phraya and Bang Pakong watersheds (Figure 7b).

### 3.5. Genetic Diversity and Habitat Suitability of Mahachai Betta and Landscape-Level Variables

No statistically significant correlations were found between the genetic diversity (*AR*, *H*_e_, and *F*_IS_) of Mahachai Betta and habitat suitability (Figure S9). Additionally, no correlation was observed between the genetic diversity (*AR*, *H*_e_, and *F*_IS_) of Mahachai Betta and landscape-level variables (Table S44).

## 4. Discussion

The mainland and numerous Southeast Asian islands, with their hot and humid climates, offer a vast array of bioresources. A greater decline in biodiversity has been observed however in freshwater than in terrestrial or marine ecosystems [48,49]. The endangerment of freshwater fish is caused by habitat fragmentation, pollution, and overfishing, with land use and climate change posing significant threats to biodiversity and genetic diversity [48,50]. Species with specific geographical ranges, such as Mahachai Betta, are threatened by environmental factors, which increase the risk of extinction due to limited migration. Population genetics and landscape analyses of Mahachai Betta in Thailand were performed to improve our understanding of the processes affecting endemic freshwater fishes in rapidly urbanizing regions. Genetic management is considered crucial to prevent species becoming threatened and ultimately extinct.

### 4.1. Bottlenecks and Low Genetic Diversity in Mahachai Betta Populations

Low genetic diversity is typically noted in species with restricted distributions owing to genetic drift or inbreeding [51]. Although low *F*_IS_ and *r*-values indicate random mating in Mahachai Betta populations, the very low *AR* and *H*_e_ levels suggest that immediate adaptation and expansion are limited. This restricts population fitness and survival in changing environments and increases the local extinction risk [52]. Low genetic diversity is likely caused by habitat fragmentation due to anthropogenic activities, such as urbanization, which has increased by approximately 5.45% in the examined area over the last 17 years. Mahachai Betta has been severely affected by this fragmentation through population isolation, reduced gene flow, decreased mating opportunities, and increased genetic drift (Figure S10). Historical bottlenecks were confirmed by *M* ratios below the critical value (*M* = 0.68) observed in all populations [53]. Additionally, recent bottlenecks were indicated in 6 of the 10 populations by significant differences in heterozygosity within and among populations. By contrast, Plakad-Pa Pak-Tawan-Ok (*Betta siamorientalis*) populations, which are distributed more widely due to a broader geographical range, showed higher *AR* and *H*_e_ values than those of Mahachai Betta, despite ongoing rapid urbanization [22]. The current *H*_e_ and *AR* of the Mahachai Betta populations were used to predict forward genetic simulations. The results indicated that genetic diversity would decline and approach homogeneity within 12.5–37.5 years, based on a 3-month generation estimate [54]. Due to their low genetic diversity, urgent genetic management is required for Mahachai Betta populations. However, bottleneck results may be biased by factors such as population size, gene flow, sample number, and locus number [55,56]. Moreover, bias was found in at least three microsatellite loci with a high probability of directional selection. Thus, analysis with a larger sample size is recommended for reliable results. Despite potential biases, bottleneck indications suggest that conservation and restoration efforts are required for specific resident populations to maintain their evolutionary potential. Although low levels of genetic diversity have been reported in Mahachai Betta, it remains unclear whether this condition is a long-standing demographic feature or a more recent outcome of anthropogenic pressures. The absence of baseline data on the original genetic background of these populations complicates efforts to disentangle historical constraints from contemporary processes. This knowledge gap highlights the need for studies that incorporate temporal or comparative reference populations to better reconstruct the genetic trajectories of this species.

Nonetheless, genetic monitoring of the SPK, SKN4, and SKN7 populations revealed a large *N*_e_ over time, while most populations exhibited an infinite number, a result that was potentially biased due to the small sample size. These biases can be minimized using enhanced sampling strategies, which should be carefully considered in future studies. Continuous genetic monitoring is essential to conserve and enhance gene pools in populations with low genetic diversity.

### 4.2. Urbanization and Environmental Factors Drive Genetic Differentiation in Mahachai Betta Populations

The SPK population of Mahachai Betta exhibited distinct genetic structures, as shown by PCoA, DAPC, and STRUCTURE analyses. This suggests that limited gene flow, caused by physical barriers, such as the Chao Phraya River, habitat fragmentation, and urbanization, reduces genetic diversity within populations and contributes to increased genetic differentiation. A significant IBD was observed, which indicated a strong correlation between genetic differentiation and geographical distance. However, no statistically significant correlations were found between genetic diversity and habitat suitability or landscape-level variables. Lower genetic differentiation was observed in Mahachai Betta in three main regions: the lower Chao Phraya River, Maha Chai Canal (SKN3 and SKN6), and the lower Tha Chin River. This aligns with the observation that the most suitable habitats in the Tha Chin watershed are distributed mainly at the junction of the flat plains in the southern Tha Chin and Chao Phraya watersheds, with some patches in the Chao Phraya and Bang Pakong watersheds, where the SPK population is located. Surprisingly, according to the watershed regions, except for SPK, the identified genetic clusters did not align with the Mahachai Betta population. They do not reflect the current hydrogeographic configuration among different watersheds or within small tributaries within the same watershed. A high genetic structure was observed on a small scale (<1.3 km) among the Mahachai Betta populations, which suggested that small-scale historical hydrographic changes explain the origin of these groups. *F*_ST_ and *R*_ST_ values were calculated to examine the extent of genetic differentiation. The infinite allele model, which accounts for mutation and migration, underpins *F*_ST_, whereas *R*_ST_ is independent of the mutation rate under the SMM [57]. Higher *F*_ST_ than *R*_ST_ values were observed in most population pairs, which suggest that gene flow contributed to the observed high genetic differentiation. Notably, unidirectional historical gene flow was observed toward SK5 and SK6 in most populations. *N*_m_ values exceeding 1.0 strongly indicate that gene flow effects dominate over drift effects [58,59], which suggests that historical gene flow has a greater impact among populations, with all migrants directed toward SK5 and SK6. The estimated gene flow aligned with the dominant direction of water flow from the eastern side to the western region. However, no evidence of a geographical connection was observed in most populations. One explanation might relate to the annual flooding events in central Thailand and the unidirectional flow toward the mouth of the Tha Chin River in the Tha Chin watershed [60,61]. This area may be influenced by the direction of flooding, which flows from natural ponds on the eastern side to the mouth of the Tha Chin River. Pond-adapted isolates can periodically transform into temporary ponds due to flooding or habitat disruption. Similarly, a model that explained how stickleback colonization [62] and flood dispersal influence copepod diversity [63] was proposed for flooding. However, the effects of environmentally mediated mechanisms on gene flow remain largely unexplored. This may also be attributed to the large population size and incomplete lineage sorting from the population’s origin to the present, as no urbanization or farming existed in the area over 50 years ago (Figure S11). However, the BKK1 and BKK2 populations are located near the eastern side of the Chao Phraya River, whereas SKN7 is positioned on the western side of the mouth of the Tha Chin River in the opposite direction (west to east). This is questionable because gene flow within a river typically follows the drainage direction from up- to downstream. Alternatively, asymmetric gene flow toward SK5 and SK6 may be explained by recent habitat changes, which have often been attributed to habitat changes in many species, including mammals, birds, amphibians, and fish [64,65,66]. More than a 10-fold increase in segments of gene flow were observed moving toward SK5 and SK6, which are considered the most suitable habitats. This supports the hypothesis that large-scale urbanization from Bangkok expanded its boundaries into the Samut Sakhon territory, which created strong barriers to gene flow within the species region. Biodiversity, genetic diversity, and population structure were negatively affected by urbanization. Increased urban development decreases gene flow and genetic variation, which accelerates potential extinction. Considering the low genetic diversity and bottlenecks among fragmented populations, conservation should be prioritized.

### 4.3. Isolated Environmental Patches: Shaping Distinct Gene Pool Patterns

Genetic differentiation via drift and selection was evaluated by correlating genetic distance with geographical distance and environmental dissimilarity, which highlights these factors as key determinants of population structure. Mahachai Betta inhabit brackish water environments and are susceptible to fluctuations in water quality and changes in vegetation. Environmental heterogeneity drives local adaptations and genetic differentiation through selective pressures from pH, salinity, and DO variations. Apart from the SPK population, which was distant from the others, SKN3 and SKN7 exhibited significantly different gene pool patterns. Despite being in the same watershed, SKN3 is genetically distinct from nearby populations such as SKN1, SKN2, and SKN4–6. High salinity and conductivity were observed in SKN3 compared with those in other populations, which contributed to the ecological niche and fitness of the SKN3 Mahachai Betta population. Environmental heterogeneity plays a greater role in genetic differentiation than geographic distance. This suggests that it is the primary reason for the substantial genetic differentiation of the SKN3 Mahachai Betta population, which lacks an obvious IBD pattern. Notably, the Samut Sakhon coastal region experiences complex interactions between freshwater and saline water. Changes in tidal and freshwater flows limit salinity from advancing and retreating. During the wet season, local rainfall and flood flows from the upland regions maintain salinity limits near the coastline. However, salinity in areas such as SKN3 is maintained under wetland conditions and floodplains due to the continuous tidal influence of the Gulf of Thailand [67]. The progressive intrusion of saline water upstream renders the region vulnerable to increasing salinity. Comparable results have been obtained for other species in natural environments, which supports the conclusion that environmental factors play a greater role than geographical distance [68,69,70]. Genetic differences and plasticity in salinity tolerance may allow populations to adapt to future salinity changes. Management and conservation efforts must consider geographically adjacent populations with different genetic characteristics to avoid adverse effects on fitness and persistence.

On the other hand, SKN7 is found under deficient DO conditions, whereas BKK1, which inhabits similar conditions, shares a similar gene pool pattern with BKK2 and other SKN populations. One possible explanation is provided by ABC analysis, which showed that SKN7 is derived from both SKN5 and SKN6. Currently, no history of the reintroduction of Mahachai Betta into Thailand exists. However, the release of cultivated wild Mahachai Betta, derived from artificial mating with various ecotypes, may obscure the relationship between landscape characteristics and gene flow by separating human-facilitated gene movement from landscape influences [5]. More SKN7 samples and additional sampling sites near SKN7 are required to examine the origin of the different gene pool patterns.

### 4.4. Using Information to Enhance Conservation Efforts

Ongoing global warming, along with the increased demand for water resources and urbanization, has exacerbated the negative impacts on the distribution range of Mahachai Betta. Thus, the identification of conservation units for a given species is considered a key element in conservation biology. These units guide management and conservation efforts when resources are limited. Genetic data are used to establish differences among populations within a species, which helps define conservation units. Practical conservation efforts may include categorizing populations into appropriate evolutionarily significant (ESUs) and management (MUs) units to tailor strategies [71]. In addition, distinct genetic structures should be identified and managed as separate ESUs and MUs [72]. For isolated populations, such as genetically distinct SPK populations with low genetic diversity, it is crucial to improve habitat connectivity to promote gene flow, which is crucial for water resource management and urban planning. However, while genetic rescue through enhanced gene flow may increase diversity and reduce inbreeding, it also carries risks such as outbreeding depression, disruption of local adaptations, or unintended homogenization of distinct genetic units [73]. Therefore, any intervention aiming to increase gene flow should be carefully evaluated and monitored within a long-term conservation framework. Reduced genetic diversity increases vulnerability to environmental changes and the risk of population decline. The SPK population should be recognized as an independent ESU and MU, whereas other populations should be managed collectively based on genetic similarity and ecological factors. BKK1 and BKK2 may be classified as the same ESU and MU, whereas SKN1–SKN7 are considered part of the same ESU but comprise three Mus: (1) SKN1, 2, 4, 5, and 6; (2) SKN3; and (3) SKN7. SKN7 should be recognized for its potential release activities and confirmed using high-throughput genetic technology. Regular monitoring of genetic diversity across different ESUs and MUs will help ensure the long-term resilience and adaptability of Mahachai Betta populations. Urban planning restrictions are recommended to facilitate recovery and habitat protection. Critical habitats should be protected for the survival and reproduction of Mahachai Betta, and local communities should be engaged in conservation efforts and educated on the importance of genetic diversity. By tailoring conservation measures to the specific genetic and ecological needs of Mahachai Betta, the preservation of its diversity and resilience can be ensured. Data and behavior among Mahachat Betta enthusiasts, however, do not support a captive breeding strategy, as this may mix different origins to produce mass populations for reintroduction, which results in the treatment of all localities within the species distribution area as a single population. Knowledge of population genetic structure is relevant for developing effective management strategies. If genetic factors are ignored, inappropriate recovery strategies may be implemented, which may lead to the potential loss of genetic structure. However, ex situ strategies may be needed if in situ conservation fails to restore populations. Despite the possibility of further reducing the genetic diversity of ESUs and MUs through captive breeding programs, these measures should be planned and implemented before the sole surviving population of these MUs becomes extinct. Given the success of recent captive breeding programs for several highly endangered freshwater fish species, this strategy is promising for the long-term preservation of the most endangered Mahachai Betta populations. Future studies examining the factors driving genetic diversity patterns in Mahachai Betta should explore alternative markers and measures of diversity, as evidence suggests that population-level processes affect genetic loci differently. We analyzed genetic diversity patterns using mean heterozygosity and rarefied allele counts from neutral markers. However, as next-generation sequencing costs decrease and data availability increases, investigations can begin to determine whether genome-wide genetic diversity patterns in Mahachai Betta are related to factors such as habitat, conservation status, and life history traits.

## 5. Conclusions

This study analyzed the genetic diversity and population structure of Mahachai Betta using microsatellite markers and landscape analyses. The results revealed significant differentiation among populations despite low overall variability, with isolation by distance clearly observed. Key environmental factors (pH, DO, conductivity, salinity), together with geographic barriers such as river systems and urban development, restrict gene flow and shape population divergence. Selective pressures also contributed to the genetic makeup of these populations. These findings highlight the urgent need to conserve Mahachai Betta in their natural habitats, particularly smaller populations, and provide a foundation for future conservation strategies.

## Figures and Tables

**Table 1:** Genetic diversity of Mahachai betta (*Betta mahachaiensis*) populations based on 13 mi-crosatellite loci.

Population		N	*N*_a_	*AR*	*N*_e_	*I*	*H*_o_	*H*_e_	*F*	*M* Ratio	*PIC*
SPK ^1^	Mean	17.000	3.222	3.308	1.881	0.697	0.314	0.381	0.201	0.322	0.425
	SE	0.166	1.575	0.472	0.223	0.128	0.091	0.066	0.134	0.277	0.217
BKK1 ^2^	Mean	11.000	2.733	3.154	1.972	0.711	0.315	0.380	0.175	0.318	0.434
	SE	0.385	1.340	0.492	0.281	0.144	0.094	0.072	0.154	0.285	0.245
BKK2 ^3^	Mean	3.000	1.769	1.769	1.491	0.346	0.308	0.201	−0.507	0.157	0.522
	SE	0.000	1.120	0.323	0.212	0.138	0.122	0.078	0.084	0.112	0.281
SKN1 ^4^	Mean	4.000	1.505	1.769	1.562	0.411	0.295	0.261	−0.030	0.320	0.564
	SE	0.122	0.693	0.231	0.179	0.119	0.113	0.074	0.202	0.311	0.131
SKN2 ^5^	Mean	20.000	4.219	4.462	2.239	0.841	0.374	0.425	0.079	0.350	0.473
	SE	0.368	2.514	0.781	0.445	0.155	0.092	0.066	0.147	0.264	0.207
SKN3 ^6^	Mean	5.000	1.696	1.923	1.483	0.398	0.342	0.240	−0.374	0.243	0.434
	SE	0.166	0.718	0.288	0.159	0.116	0.116	0.069	0.152	0.234	0.207
SKN4 ^7^	Mean	10.000	3.122	3.308	2.010	0.812	0.439	0.464	0.091	0.387	0.530
	SE	0.166	1.319	0.444	0.147	0.098	0.097	0.047	0.165	0.298	0.102
SKN5 ^8^	Mean	3.000	1.502	2.308	1.951	0.647	0.359	0.393	0.043	0.379	0.653
	SE	0.166	0.331	0.286	0.224	0.127	0.116	0.072	0.205	0.261	0.210
SKN6 ^9^	Mean	3.000	2.092	2.462	2.051	0.703	0.462	0.423	−0.108	0.301	0.606
	SE	0.077	0.710	0.312	0.267	0.123	0.110	0.065	0.176	0.212	0.179
SKN7 ^10^	Mean	5.000	1.288	2.000	1.530	0.429	0.238	0.258	0.072	0.345	0.534
	SE	0.312	0.277	0.340	0.161	0.124	0.076	0.072	0.109	0.147	0.103
All Population	Mean	7.800	3.308	2.646	2.120	0.773	0.422	0.421	0.010	0.312	0.517
	SE	0.513	0.190	0.149	0.092	0.045	0.034	0.022	0.051	0.240	0.188

Sample size (N); average number of alleles per locus (*N*_a_); allelic richness (*AR*); number of effective alleles (*N*_e_); Shannon’s information index (*I*); observed heterozygosity (*H*_o_); expected heterozygosity (*H*_e_); ratio of number of allele to allele range (*M* ratio); polymorphic information content (*PIC*); fixation index (*F*). ^1^ SPK = Samut Prakan. ^2^ BKK = Bangkok 1. ^3^ BKK2 = Bangkok 2. ^4^ SKN1 = Samut Sakhon 1. ^5^ SKN2 = Samut Sakhon 2. ^6^ SKN3 = Samut Sakhon 3. ^7^ SKN4 = Samut Sakhon 4. ^8^ SKN5 = Samut Sakhon 5. ^9^ SKN6 = Samut Sakhon 6. ^10^ SKN7 = Samut Sakhon 7.

**Table 2:** Comparison of observed (*H*_o_) and expected (*H*_e_) Heterozygosity values between ten populations of Mahachai betta (*Betta mahachaiensis*) based on 13 microsatellite.

Population	*H*_o_	*H*_e_	df	*t*-Test	*p*-Value
SPK ^1^	0.314 ± 0.091	0.381 ± 0.066	−0.067	−0.596	0.556
BKK1 ^2^	0.315 ± 0.094	0.380 ± 0.072	−0.065	−0.549	0.590
BKK2 ^3^	0.308 ± 0.122	0.201 ± 0.078	0.107	0.739	0.508
SKN1 ^4^	0.295 ± 0.113	0.261 ± 0.074	0.034	0.252	0.811
SKN2 ^5^	0.374 ± 0.092	0.425 ± 0.066	−0.051	−0.450	0.655
SKN3 ^6^	0.342 ± 0.116	0.240 ± 0.069	0.102	0.756	0.476
SKN4 ^7^	0.439 ± 0.097	0.464 ± 0.047	−0.025	−0.232	0.820
SKN5 ^8^	0.359 ± 0.116	0.393 ± 0.072	−0.034	−0.249	0.818
SKN6 ^9^	0.462 ± 0.110	0.423 ± 0.065	0.039	0.305	0.779
SKN7 ^10^	0.238 ± 0.076	0.258 ± 0.072	−0.020	−0.191	0.853

df = Difference in means. ^1^ SPK = Samut Prakan. ^2^ BKK = Bangkok 1. ^3^ BKK2 = Bangkok 2. ^4^ SKN1 = Samut Sakhon 1. ^5^ SKN2 = Samut Sakhon 2. ^6^ SKN3 = Samut Sakhon 3. ^7^ SKN4 = Samut Sakhon 4. ^8^ SKN5 = Samut Sakhon 5. ^9^ SKN6 = Samut Sakhon 6. ^10^ SKN7 = Samut Sakhon 7.

## Data Availability

The genotypic data generated in this study were deposited in the Dryad Digital Repository Dataset (http://datadryad.org/stash/share/0X0PI6WHzfvdInPokqtB20m-hQ-GivHyMIQs2XhrzlA, accessed on 4 November 2024).

## Supplementary Materials

The following supporting information can be downloaded at: https://www.mdpi.com/article/10.3390/ani15192820/s1, Figure S1: Water quality in the habitats of populations of Mahachai Betta (*Betta mahachaiensis*). (a) pH, (b) dissolved oxygen, (c) conductivity, and (d) salinity.; Figure S2: Simulation results illustrating the intergenerational connections in terms of (a) expected heterozygosity and (b) allelic richness; Figure S3: (a) Observed distribution of relatedness (*r*) in Mahachai Betta (*Betta mahachaiensis*) populations plotted against expected distributions. (b) Observed distribution of inbreeding coefficients (*F*_IS_) in Mahachai Betta (*Betta mahachaiensis*) populations plotted against expected distributions; Figure S4: Mapping of expected heterozygosity (*H*_e_) against inbreeding coefficients (*F*_IS_) along the length of the physical map. (a) Mahachai Betta (*Betta mahachaiensis*) populations. (b) microsatellite loci; Figure S5: Population structure of the 10 Mahachai Betta (*Betta mahachaiensis*) populations, (a) Evanno’s Δ*K* and (b) ln P(*K*) plots; Figure S6: Discriminant analysis of principal components (DAPC) for Mahachai Betta (*Betta mahachaiensis*) based on 13 microsatellite loci; Figure S7: Loci bias in Mahachai Betta (*Betta mahachaiensis*) populations; Figure S8: (a) Historical gene flow dynamics based on MIGRATE-N among *Betta mahachaiensis* populations over space (only values ≥500 are shown). (b) Recent gene flow dynamics based on BayesAss among *B. mahachaiensis* populations (only values ≥0.05 are shown). The width of the curves represents the relative magnitude of migration; Figure S9: Relationships between genetic diversity and habitat suitability in Mahachai Betta (*Betta mahachaiensis*). (a) Allelic richness (ρ = −0.042, p = 0.918). (b) Expected heterozygosity (ρ = 0.127, p = 0.732). (c) Inbreeding coefficients (FIS) (ρ = 0.030, p = 0.945); Figure S10; Land-use types in 2002 (a) and 2019 (b) in the study area. Source: Land Development Department (2021); Figure S11: Combination bands 7, 6, and 5 from Landsat 1 view on January 7, 1973 (a) and February 14, 2024 (b). In this false-color image, shades of green indicate vegetated land; Table S1: Summary of Mahachai Betta (*Betta mahachaiensis*) individuals sampled in this study; Table S2: Microsatellite primers and sequences used in this study; Table S3: Mahachai Betta (*Betta mahachaiensis*) occurrence points considering geographic coordinates, genetic diversity, habitat suitability value, and landscape-level variables; Table S4: Genetic diversity of 81 Mahachai Betta (*Betta mahachaiensis*) individuals based on 13 microsatellite loci; Table S5: Comparison of genetic diversity parameters between Mahachai Betta (*Betta mahachaiensis*) individuals based on 13 microsatellite loci; Table S6: Hardy–Weinberg and linkage disequilibrium analysis of the alleles of 13 microsatellites of Mahachai Betta (*Betta mahachaiensis*) individuals from SPK; Table S7: Hardy–Weinberg and linkage disequilibrium analysis of the alleles of 13 microsatellites of Mahachai Betta (*Betta mahachaiensis*) individuals from BKK1; Table S8: Hardy–Weinberg and linkage disequilibrium analysis of the alleles of 13 microsatellites of Mahachai Betta (*Betta mahachaiensis*) individuals from BKK2; Table S9: Hardy–Weinberg and linkage disequilibrium analysis of the alleles of 13 microsatellites of Mahachai Betta (*Betta mahachaiensis*) individuals from SPK1; Table S10: Hardy–Weinberg and linkage disequilibrium analysis of the alleles of 13 microsatellites of Mahachai Betta (*Betta mahachaiensis*) individuals from SPK2; Table S11: Hardy–Weinberg and linkage disequilibrium analysis of the alleles of 13 microsatellites of Mahachai Betta (*Betta mahachaiensis*) individuals from SPK3; Table S12: Hardy–Weinberg and linkage disequilibrium analysis of the alleles of 13 microsatellites of Mahachai Betta (*Betta mahachaiensis*) individuals from SPK4; Table S13: Hardy–Weinberg and linkage disequilibrium analysis of the alleles of 13 microsatellites of Mahachai Betta (*Betta mahachaiensis*) individuals from SPK5; Table S14: Hardy–Weinberg and linkage disequilibrium analysis of the alleles of 13 microsatellites of Mahachai Betta (*Betta mahachaiensis*) individuals from SPK6; Table S15: Hardy–Weinberg and linkage disequilibrium analysis of the alleles of 13 microsatellites of Mahachai Betta (*Betta mahachaiensis*) individuals from SPK7; Table S16: Pairwise genetic relatedness (*r*) of all 17 Mahachai Betta (*Betta mahachaiensis*) individuals from SPK; Table S17: Inbreeding coefficients, relatedness, effective population size, and the ratio of effective population size and census population (*N*_e_*/N*) of the 81 Mahachai Betta (*Betta mahachaiensis*) individuals; Table S18: Pairwise genetic relatedness (*r*) of all 11 Mahachai Betta (*Betta mahachaiensis*) individuals from BKK1; Table S19: Pairwise genetic relatedness (*r*) of all three Mahachai Betta (*Betta mahachaiensis*) individuals from BKK2; Table S20: Pairwise genetic relatedness (*r*) of all four Mahachai Betta (*Betta mahachaiensis*) individuals from SKN1; Table S21: Pairwise genetic relatedness (*r*) of all 20 Mahachai Betta (*Betta mahachaiensis*) individuals from SKN2; Table S22: Pairwise genetic relatedness (*r*) of all five Mahachai Betta (*Betta mahachaiensis*) individuals from SKN3; Table S23: Pairwise genetic relatedness (*r*) of all 10 Mahachai Betta (*Betta mahachaiensis*) individuals from SKN4; Table S24: Pairwise genetic relatedness (*r*) of all three Mahachai Betta (*Betta mahachaiensis*) individuals from SKN5; Table S25: Pairwise genetic relatedness (*r*) of all three Mahachai Betta (*Betta mahachaiensis*) individuals from SKN6; Table S26: Pairwise genetic relatedness (*r*) of all five Mahachai Betta (*Betta mahachaiensis*) individuals from SKN7; Table S27: Distributions of *r* and *F*_IS_ values for Mahachai Betta (*Betta mahachaiensis*); Table S28: Pairwise inbreeding coefficients (*F*_IS_) of all 17 Mahachai Betta (*Betta mahachaiensis*) individuals from SPK; Table S29: Pairwise inbreeding coefficients (*F*_IS_) of all 11 Mahachai Betta (*Betta mahachaiensis*) individuals from BKK1; Table S30: Pairwise inbreeding coefficients (*F*_IS_) of all three Mahachai Betta (*Betta mahachaiensis*) individuals from BKK2; Table S31: Pairwise inbreeding coefficients (*F*_IS_) of all four Mahachai Betta (*Betta mahachaiensis*) individuals from SKN1; Table S32: Pairwise inbreeding coefficients (*F*_IS_) of all 20 Mahachai Betta (*Betta mahachaiensis*) individuals from SKN2; Table S33: Pairwise inbreeding coefficients (*F*_IS_) of all five Mahachai Betta (*Betta mahachaiensis*) individuals from SKN3; Table S34: Pairwise inbreeding coefficients (*F*_IS_) of all 10 Mahachai Betta (*Betta mahachaiensis*) individuals from SKN4; Table S35: Pairwise inbreeding coefficients (*F*_IS_) of all three Mahachai Betta (*Betta mahachaiensis*) individuals from SKN5; Table S36: Pairwise inbreeding coefficients (*F*_IS_) of all three Mahachai Betta (*Betta mahachaiensis*) individuals from SKN6; Table S37: Pairwise inbreeding coefficients (*F*_IS_) of all five Mahachai Betta (*Betta mahachaiensis*) individuals from SKN7; Table S38: Pairwise genetic differentiation (*F_ST_*), pairwise *F*_ST_^ENA^ values with ENA correction for null alleles, and *R*_ST_ values determined using FSTAT version 2:9:3 (Goudet, 1995) of Mahachai Betta (*Betta mahachaiensis*) based on 13 microsatellite loci: The number indicates *p*-values with 110 permutations; Table S39: Analysis of molecular variance (AMOVA) results for Mahachai Betta (*Betta mahachaiensis*) individuals based on 13 microsatellite loci using Arlequin version 3:5:2:2 (Excoffier & Lischer, 2010); Table S40: Pairwise population Nei’s genetic distance (*D*) values (GenAlEx version 6:5) (Peakall & Smouse, 2012) of Mahachai Betta (*Betta mahachaiensis*) individuals based on 13 microsatellite loci; Table S41: Wilcoxon signed-rank test to assess mutation drift equilibrium across various models in the 81 samples of Mahachai Betta (*Betta mahachaiensis*); Table S42: All source/recipient population comparisons with the mean migration rates and 95% confidence intervals determined by BAYESASS using microsatellite data; Table S43: Bayesian estimates of mutation-scaled effective population sizes (Θ) and asymmetric migration rates (*M*) calculated among the 81 Mahachai Betta (*Betta mahachaiensis*) individuals across 13 microsatellite loci; Table S44: The effective number of immigrants (*N*_m_) from population *i* to population *j* per generation; Table S45: Multiple linear regression model of the genetic diversity and habitat suitability of Mahachai Betta (*Betta mahachaiensis*) considering landscape-level variables.

## Abbreviations

The following abbreviations are used in this manuscript:

ABC	Approximate Bayesian Computation
AMOVA	Analysis of Molecular Variance
AR	Allelic richness
BAM	Biotic–Abiotic–Movement framework
DAPC	Discriminant Analysis of Principal Components
DO	Dissolved Oxygen
ESUs	Evolutionarily Significant Units
F	Fixation index
IBD	Isolation by Distance
LSI	Landscape Shape Interpolation
MUs	Management Units
PCoA	Principal Coordinate Analysis
PIC	Polymorphic information content
PSU	Practical Salinity
