# Supplementary material for: Saving the Mahachai Betta: Genetic Erosion and Conservation Priorities Under Urbanization Pressure
Source: Animals (Basel). 2025 Sep 26;15(19):2820. doi: 10.3390/ani15192820 (PMC12523270; doi:10.3390/ani15192820)
Supplement: Supplementary file 1 [file animals-15-02820-s001.zip › animals-3881614-supplementary.pdf]

Table S1. Summary of Mahachai betta (*Betta mahachaiensis*) individuals sampled in this study

No.	Abbreviation/Code	Coordinate	Locality
1	SPK-1	13°36'06.7"N 100°32'37.4"E	Samut Prakan
2	SPK-2	13°36'06.7"N 100°32'37.4"E	Samut Prakan
3	SPK-3	13°36'06.7"N 100°32'37.4"E	Samut Prakan
4	SPK-4	13°36'06.7"N 100°32'37.4"E	Samut Prakan
5	SPK-5	13°36'06.7"N 100°32'37.4"E	Samut Prakan
6	SPK-6	13°36'06.7"N 100°32'37.4"E	Samut Prakan
7	SPK-7	13°36'06.7"N 100°32'37.4"E	Samut Prakan
8	SPK-8	13°36'06.7"N 100°32'37.4"E	Samut Prakan
9	SPK-9	13°36'06.7"N 100°32'37.4"E	Samut Prakan
10	SPK-10	13°36'06.7"N 100°32'37.4"E	Samut Prakan
11	SPK-11	13°36'06.7"N 100°32'37.4"E	Samut Prakan
12	SPK-12	13°36'06.7"N 100°32'37.4"E	Samut Prakan
13	SPK-13	13°36'06.7"N 100°32'37.4"E	Samut Prakan
14	SPK-14	13°36'06.7"N 100°32'37.4"E	Samut Prakan
15	SPK-15	13°36'06.7"N 100°32'37.4"E	Samut Prakan
16	SPK-16	13°36'06.7"N 100°32'37.4"E	Samut Prakan
17	SPK-17	13°36'06.7"N 100°32'37.4"E	Samut Prakan
18	BKK1-1	13°35'43.8"N 100°24'04.2"E	Bangkok
19	BKK1-2	13°35'43.8"N 100°24'04.2"E	Bangkok
20	BKK1-3	13°35'43.8"N 100°24'04.2"E	Bangkok
21	BKK1-4	13°35'43.8"N 100°24'04.2"E	Bangkok
22	BKK1-5	13°35'43.8"N 100°24'04.2"E	Bangkok
23	BKK1-6	13°35'43.8"N 100°24'04.2"E	Bangkok
24	BKK1-7	13°35'43.8"N 100°24'04.2"E	Bangkok
25	BKK1-8	13°35'43.8"N 100°24'04.2"E	Bangkok
26	BKK1-9	13°35'43.8"N 100°24'04.2"E	Bangkok
27	BKK1-10	13°35'43.8"N 100°24'04.2"E	Bangkok
28	BKK1-11	13°35'43.8"N 100°24'04.2"E	Bangkok
29	BKK2-1	13°35'30.0"N 100°23'22.7"E	Bangkok
30	BKK2-2	13°35'30.0"N 100°23'22.7"E	Bangkok
31	BKK2-3	13°35'30.0"N 100°23'22.7"E	Bangkok
32	SKN1-1	13°34'52.7"N 100°21'40.9"E	Samut Sakhon

No.	Abbreviation/Code	Coordinate	Locality
33	SKN1-2	13°34'52.7"N 100°21'40.9"E	Samut Sakhon
34	SKN1-3	13°34'52.7"N 100°21'40.9"E	Samut Sakhon
35	SKN1-4	13°34'52.7"N 100°21'40.9"E	Samut Sakhon
36	SKN2-1	13°34'33.2"N 100°18'33.1"E	Samut Sakhon
37	SKN2-2	13°34'33.2"N 100°18'33.1"E	Samut Sakhon
38	SKN2-3	13°34'33.2"N 100°18'33.1"E	Samut Sakhon
39	SKN2-4	13°34'33.2"N 100°18'33.1"E	Samut Sakhon
40	SKN2-5	13°34'33.2"N 100°18'33.1"E	Samut Sakhon
41	SKN2-6	13°34'33.2"N 100°18'33.1"E	Samut Sakhon
42	SKN2-7	13°34'33.2"N 100°18'33.1"E	Samut Sakhon
43	SKN2-8	13°34'33.2"N 100°18'33.1"E	Samut Sakhon
44	SKN2-9	13°34'33.2"N 100°18'33.1"E	Samut Sakhon
45	SKN2-10	13°34'33.2"N 100°18'33.1"E	Samut Sakhon
46	SKN2-11	13°34'33.2"N 100°18'33.1"E	Samut Sakhon
47	SKN2-12	13°34'33.2"N 100°18'33.1"E	Samut Sakhon
48	SKN2-13	13°34'33.2"N 100°18'33.1"E	Samut Sakhon
49	SKN2-14	13°34'33.2"N 100°18'33.1"E	Samut Sakhon
50	SKN2-15	13°34'33.2"N 100°18'33.1"E	Samut Sakhon
51	SKN2-16	13°34'33.2"N 100°18'33.1"E	Samut Sakhon
52	SKN2-17	13°34'33.2"N 100°18'33.1"E	Samut Sakhon
53	SKN2-18	13°34'33.2"N 100°18'33.1"E	Samut Sakhon
54	SKN2-19	13°34'33.2"N 100°18'33.1"E	Samut Sakhon
55	SKN2-20	13°34'33.2"N 100°18'33.1"E	Samut Sakhon
56	SKN3-1	13°33'36.6"N 100°18'23.2"E	Samut Sakhon
57	SKN3-2	13°33'36.6"N 100°18'23.2"E	Samut Sakhon
58	SKN3-3	13°33'36.6"N 100°18'23.2"E	Samut Sakhon
59	SKN3-4	13°33'36.6"N 100°18'23.2"E	Samut Sakhon
60	SKN3-5	13°33'36.6"N 100°18'23.2"E	Samut Sakhon
61	SKN4-1	13°33'53.0"N 100°15'10.5"E	Samut Sakhon
62	SKN4-2	13°33'53.0"N 100°15'10.5"E	Samut Sakhon
63	SKN4-3	13°33'53.0"N 100°15'10.5"E	Samut Sakhon
64	SKN4-4	13°33'53.0"N 100°15'10.5"E	Samut Sakhon
65	SKN4-5	13°33'53.0"N 100°15'10.5"E	Samut Sakhon
66	SKN4-6	13°33'53.0"N 100°15'10.5"E	Samut Sakhon

No.	Abbreviation/Code	Coordinate	Locality
67	SKN4-7	13°33'53.0"N 100°15'10.5"E	Samut Sakhon
68	SKN4-8	13°33'53.0"N 100°15'10.5"E	Samut Sakhon
69	SKN4-9	13°33'53.0"N 100°15'10.5"E	Samut Sakhon
70	SKN4-10	13°33'53.0"N 100°15'10.5"E	Samut Sakhon
71	SKN5-1	13°33'17.2"N 100°15'34.6"E	Samut Sakhon
72	SKN5-2	13°33'17.2"N 100°15'34.6"E	Samut Sakhon
73	SKN5-3	13°33'17.2"N 100°15'34.6"E	Samut Sakhon
74	SKN6-1	13°33'59.0"N 100°16'30.6"E	Samut Sakhon
75	SKN6-2	13°33'59.0"N 100°16'30.6"E	Samut Sakhon
76	SKN6-3	13°33'59.0"N 100°16'30.6"E	Samut Sakhon
77	SKN7-1	13°31'51.5"N 100°12'22.7"E	Samut Sakhon
78	SKN7-2	13°31'51.5"N 100°12'22.7"E	Samut Sakhon
79	SKN7-3	13°31'51.5"N 100°12'22.7"E	Samut Sakhon
80	SKN7-4	13°31'51.5"N 100°12'22.7"E	Samut Sakhon
81	SKN7-5	13°31'51.5"N 100°12'22.7"E	Samut Sakhon

Table S2. Microsatellite primers and sequences.

Primer	Fluorescence	Primer sequence 5′ to 3′		Size (bp)
		Reverse	Forward	
BettaMS4	FAM	GTTTCATCAGGAGCAGCAGCATAA	CTGTTTGATGGCCGACTTTT	259 – 315
BettaMS5	HEX	GTTTCGTCACCTTCTGAGCAAACA	AAATGCGCTGGGTAGACTTG	198 – 218
BettaMS8	FAM	CGTGAGCTGCAAAGAAAACA	GCTGTTGCACATGAATCCAG	223
BettaMS15	HEX	ACTGTAACCGGGCTGTTCTG	AACGCACCCAGAAACAAATC	194 – 221
BettaMS17	HEX	AAGCAGGTCTTTCACCTCCA	TCACCCTGCGTCTAAGTCAA	277 – 296
BettaMS23	FAM	GTTTGAGAGAAATGGGTTCTTCG	TCACTACGCTGCCAAATCAG	194 – 224
BettaMS25	FAM	GTTTGGGTAAAACCCAACTCTGG	AACGTCACGTGGAACAGATG	136 – 165
BettaMS28	HEX	GTTTCTATTGCCTTAGGGCTCCA	TGCTCCTGAGAGGACTATTGG	280 – 313
BettaMS40	HEX	CAGTACATTTGACTGATCGCAGA	CAGGATGCTTCCTTGGGTAA	324 – 396
BettaMS2.2	FAM	ATTCCTTTCTGCCGCTAA	AAAGAGGGCACTAAGCCA	259-315
BettaMS10.1	FAM	TCTGAGGAAGGAGGCGATTA	GCGTGCACTGAAGCATAAAG	165 – 199
BettaMS14.1	FAM	GGGCTGCACCTTAAACTCAT	GTCCACTGGGCTGATGTTCT	228
BettaMS14.2	HEX	CCCGGTTTCTTGTCATTC	CGCTGATGGAAATTGAGT	198 – 218

Table S3. *B. mahachaiensis* occurrence points in geographic coordinate, genetic diversity and habitat suitability value and land scape level variables.

ID	Code	Longitude	Latitude	*AR*	F_{IS}	H_e	HBS	pH	DO (mgL^{-1})	Conductivity (mgL^{-1})	Salinity (PSU)	Water Temperature (°C)	Precipitation* (mm)	Temperature* (°C)	Elevation (m)
1	SPK	100.54	13.60	3.222	–0.157	0.381	0.059	7.96	2.41	6.07	3.30	28.00	126.49	28.91	3.00
2	BKK1	100.40	13.59	2.733	–0.207	0.380	0.427	8.25	0.46	20.14	11.90	27.00	120.79	28.81	5.00
3	BKK2	100.38	13.59	1.769	–0.756	0.201	0.206	8.07	1.95	10.34	5.85	29.00	120.12	28.79	7.00
4	SKN1	100.36	13.58	1.505	–0.586	0.261	0.881	8.47	4.59	7.32	4.11	28.00	118.57	28.76	4.00
5	SKN2	100.30	13.57	4.219	–0.134	0.425	0.606	7.90	2.52	1.44	0.75	29.00	115.65	28.68	5.00
6	SKN3	100.30	13.56	1.696	–0.650	0.240	0.541	7.99	5.65	38.05	23.90	29.00	114.95	28.66	7.00
7	SKN4	100.25	13.56	3.122	–0.236	0.464	0.331	8.59	5.47	21.74	13.00	28.00	113.12	28.61	7.00
8	SKN5	100.25	13.55	1.502	–0.256	0.393	0.373	7.07	1.79	7.33	4.05	28.00	113.07	28.61	7.00
9	SKN6	100.27	13.56	2.092	–0.391	0.423	0.330	7.10	1.74	23.55	14.20	28.00	114.01	28.64	7.00
10	SKN7	100.20	13.53	1.288	–0.376	0.258	0.236	7.95	0.39	11.40	6.45	28.00	110.52	28.55	5.00

Remark. *AR*= Allelic Richness, H_e= Expected Heterozygosity, F_{IS}= Inbreeding coefficient, , HBS=Habitat suitability. *= Annual mean temperature (°C) and Annual mean precipitation between 1981–2010 were obtained from meteorological stations of the Thailand Meteorological Department (TMD).

Table S4. Genetic diversity of 81 Mahachai betta (*Betta mahachaiensis*) individuals based on 13 microsatellite loci.

Population	Locus	N	*AR*	N_a	N_e	*I*	H_o	H_e	*F*	*M* ratio	*PIC*
SPK [1]	BetaMS4	15.000	3.000	3.000	1.822	0.770	0.200	0.451	0.557	0.060	0.467
	BetaMS5	17.000	5.870	6.000	2.580	1.293	0.824	0.612	–0.345	0.273	0.631
	BetaMS8	17.000	1.999	2.000	1.192	0.298	0.176	0.161	–0.097	0.500	0.166
	BetaMS15	17.000	2.765	3.000	1.127	0.264	0.059	0.112	0.477	0.167	0.116
	BetaMS17	17.000	2.989	3.000	1.615	0.678	0.000	0.381	1.000	0.750	0.392
	BetaMS23	16.000	2.000	2.000	2.000	0.693	0.000	0.500	1.000	1.000	0.516
	BetaMS25	17.000	2.000	2.000	1.993	0.691	0.471	0.498	0.056	0.222	0.513
	BetaMS28	17.000	1.000	1.000	1.000	0.000	0.000	0.000	N/A	N/A	0.000
	BetaMS40	17.000	5.753	6.000	2.752	1.277	0.941	0.637	–0.478	0.231	0.656
	BetaMS2.2	17.000	5.861	6.000	3.753	1.482	0.706	0.734	0.038	0.130	0.756
	BetaMS10.1	17.000	1.882	2.000	1.061	0.133	0.059	0.057	–0.030	0.200	0.059
	BetaMS14.1	17.000	3.000	3.000	2.198	0.929	0.353	0.545	0.352	0.044	0.561
	BetaMS14.2	17.000	3.764	4.000	1.357	0.557	0.294	0.263	–0.118	0.286	0.271
	Mean	16.769	3.222	3.308	1.881	0.697	0.314	0.381	0.393	0.322	0.425
	SE	0.166	1.575	0.472	0.223	0.128	0.091	0.066	0.068	0.277	0.217
BKK1 [2]	BetaMS4	6.000	3.000	3.000	2.880	1.078	0.167	0.653	0.745	0.055	0.712
	BetaMS5	11.000	4.078	5.000	2.262	1.136	0.636	0.558	–0.141	0.227	0.584
	BetaMS8	11.000	1.545	2.000	1.095	0.185	0.091	0.087	–0.048	0.500	0.091
	BetaMS15	11.000	1.545	2.000	1.095	0.185	0.091	0.087	–0.048	0.167	0.091
	BetaMS17	11.000	2.776	3.000	1.754	0.760	0.000	0.430	1.000	0.750	0.450
	BetaMS23	10.000	1.853	2.000	1.220	0.325	0.000	0.180	1.000	1.000	0.189
	BetaMS25	11.000	1.971	2.000	1.424	0.474	0.182	0.298	0.389	0.222	0.312
	BetaMS28	11.000	1.000	1.000	1.000	0.000	0.000	0.000	N/A	N/A	0.000
	BetaMS40	10.000	5.456	7.000	3.333	1.541	1.000	0.700	–0.429	0.292	0.737
	BetaMS2.2	10.000	2.200	3.000	1.227	0.394	0.200	0.185	–0.081	0.100	0.195
	BetaMS10.1	11.000	1.999	2.000	1.766	0.625	0.636	0.434	–0.467	0.057	0.455
	BetaMS14.1	11.000	5.185	6.000	4.321	1.606	0.273	0.769	0.645	0.075	0.805
	BetaMS14.2	11.000	2.919	3.000	2.262	0.937	0.818	0.558	–0.467	0.375	0.584
	Mean	10.385	2.733	3.154	1.972	0.711	0.315	0.380	0.400	0.318	0.434
	SE	0.385	1.340	0.492	0.281	0.144	0.094	0.072	0.076	0.285	0.245
BKK2 [3]	BetaMS4	3.000	1.000	1.000	1.000	0.000	0.000	0.000	N/A	N/A	0.000
	BetaMS5	3.000	3.000	3.000	2.000	0.868	0.667	0.500	–0.333	0.167	0.600
	BetaMS8	3.000	1.000	1.000	1.000	0.000	0.000	0.000	N/A	N/A	0.000
	BetaMS15	3.000	2.000	2.000	1.385	0.451	0.333	0.278	–0.200	0.333	0.333
	BetaMS17	3.000	1.000	1.000	1.000	0.000	0.000	0.000	N/A	N/A	0.000
	BetaMS23	3.000	1.000	1.000	1.000	0.000	0.000	0.000	N/A	N/A	0.000
	BetaMS25	3.000	1.000	1.000	1.000	0.000	0.000	0.000	N/A	N/A	0.000
	BetaMS28	3.000	1.000	1.000	1.000	0.000	0.000	0.000	N/A	N/A	0.000
	BetaMS40	3.000	4.000	4.000	3.000	1.242	1.000	0.667	–0.500	0.133	0.800
	BetaMS2.2	3.000	1.000	1.000	1.000	0.000	0.000	0.000	N/A	N/A	0.000
	BetaMS10.1	3.000	2.000	2.000	2.000	0.693	1.000	0.500	–1.000	0.057	0.600

Population	Locus	N	*AR*	N_a	N_e	*I*	H_o	H_e	*F*	*M* ratio	*PIC*
	BetaMS14.1	3.000	1.000	1.000	1.000	0.000	0.000	0.000	N/A	N/A	0.000
	BetaMS14.2	3.000	4.000	4.000	3.000	1.242	1.000	0.667	–0.500	0.250	0.800
	Mean	3.000	1.769	1.769	1.491	0.346	0.308	0.201	0.241	0.157	0.522
	SE	0.000	1.120	0.323	0.212	0.138	0.122	0.078	0.093	0.112	0.281
SKN1 [4]	BetaMS4	3.000	1.000	1.000	1.000	0.000	0.000	0.000	N/A	N/A	0.000
	BetaMS5	4.000	2.750	3.000	2.462	0.974	1.000	0.594	–0.684	0.167	0.679
	BetaMS8	4.000	1.000	1.000	1.000	0.000	0.000	0.000	N/A	N/A	0.000
	BetaMS15	4.000	1.000	1.000	1.000	0.000	0.000	0.000	N/A	N/A	0.000
	BetaMS17	4.000	1.964	2.000	1.600	0.562	0.000	0.375	1.000	1.000	0.429
	BetaMS23	4.000	1.000	1.000	1.000	0.000	0.000	0.000	N/A	N/A	0.000
	BetaMS25	3.000	1.000	2.000	1.800	0.637	0.667	0.444	–0.500	0.222	0.533
	BetaMS28	4.000	1.000	1.000	1.000	0.000	0.000	0.000	N/A	N/A	0.000
	BetaMS40	4.000	2.929	3.000	2.667	1.040	1.000	0.625	–0.600	0.500	0.714
	BetaMS2.2	4.000	1.000	1.000	1.000	0.000	0.000	0.000	N/A	N/A	0.000
	BetaMS10.1	3.000	1.000	3.000	2.571	1.011	0.667	0.611	–0.091	0.060	0.733
	BetaMS14.1	4.000	1.964	2.000	1.600	0.562	0.000	0.375	1.000	0.042	0.429
	BetaMS14.2	4.000	1.964	2.000	1.600	0.562	0.500	0.375	–0.333	0.250	0.429
	Mean	3.769	1.505	1.769	1.562	0.411	0.295	0.261	0.303	0.320	0.564
	SE	0.122	0.693	0.231	0.179	0.119	0.113	0.074	0.086	0.311	0.131
SKN2 [5]	BetaMS4	17.000	5.824	6.000	1.914	1.023	0.471	0.478	0.014	0.150	0.492
	BetaMS5	20.000	4.564	5.000	2.332	1.030	0.900	0.571	–0.575	0.227	0.586
	BetaMS8	20.000	4.200	5.000	1.231	0.464	0.200	0.188	–0.067	0.278	0.192
	BetaMS15	16.000	3.000	3.000	1.210	0.371	0.188	0.174	–0.079	0.167	0.179
	BetaMS17	20.000	2.999	3.000	2.381	0.943	0.000	0.580	1.000	0.750	0.595
	BetaMS23	19.000	2.000	2.000	1.633	0.576	0.000	0.388	1.000	1.000	0.398
	BetaMS25	20.000	2.000	2.000	1.980	0.688	0.500	0.495	–0.010	0.222	0.508
	BetaMS28	20.000	1.000	1.000	1.000	0.000	0.000	0.000	N/A	N/A	0.000
	BetaMS40	20.000	7.686	8.000	3.376	1.597	1.000	0.704	–0.421	0.364	0.722
	BetaMS2.2	20.000	1.999	2.000	1.220	0.325	0.200	0.180	–0.111	0.500	0.185
	BetaMS10.1	20.000	3.600	4.000	1.946	0.826	0.650	0.486	–0.337	0.080	0.499
	BetaMS14.1	20.000	10.287	11.000	7.143	2.133	0.250	0.860	0.709	0.125	0.882
	BetaMS14.2	20.000	5.687	6.000	1.735	0.952	0.500	0.424	–0.180	0.333	0.435
	Mean	19.385	4.219	4.462	2.239	0.841	0.374	0.425	0.436	0.350	0.473
	SE	0.368	2.514	0.781	0.445	0.155	0.092	0.066	0.068	0.264	0.207
SKN3 [6]	BetaMS4	4.000	1.750	2.000	1.280	0.377	0.250	0.219	–0.143	0.080	0.250
	BetaMS5	5.000	2.467	3.000	1.852	0.802	0.600	0.460	–0.304	0.750	0.511
	BetaMS8	5.000	1.000	1.000	1.000	0.000	0.000	0.000	N/A	N/A	0.000
	BetaMS15	3.000	1.000	1.000	1.000	0.000	0.000	0.000	N/A	N/A	0.000
	BetaMS17	5.000	1.000	1.000	1.000	0.000	0.000	0.000	N/A	N/A	0.000
	BetaMS23	5.000	1.000	1.000	1.000	0.000	0.000	0.000	N/A	N/A	0.000
	BetaMS25	5.000	2.000	2.000	2.000	0.693	1.000	0.500	–1.000	0.222	0.556
	BetaMS28	5.000	1.000	1.000	1.000	0.000	0.000	0.000	N/A	N/A	0.000
	BetaMS40	5.000	3.167	4.000	2.778	1.168	1.000	0.640	–0.563	0.182	0.711

Population	Locus	N	*AR*	N_a	N_e	*I*	H_o	H_e	*F*	*M* ratio	*PIC*
	BetaMS2.2	5.000	1.000	1.000	1.000	0.000	0.000	0.000	N/A	N/A	0.000
	BetaMS10.1	5.000	2.000	2.000	2.000	0.693	1.000	0.500	–1.000	0.057	0.556
	BetaMS14.1	5.000	2.467	3.000	1.852	0.802	0.200	0.460	0.565	0.034	0.511
	BetaMS14.2	5.000	2.200	3.000	1.515	0.639	0.400	0.340	–0.176	0.375	0.378
	Mean	4.769	1.696	1.923	1.483	0.398	0.342	0.240	0.267	0.243	0.434
	SE	0.166	0.718	0.288	0.159	0.116	0.116	0.069	0.077	0.234	0.207
SKN4 [7]	BetaMS4	9.000	2.889	3.000	1.742	0.730	0.556	0.426	–0.304	0.100	0.451
	BetaMS5	10.000	3.796	4.000	2.564	1.110	0.900	0.610	–0.475	0.333	0.642
	BetaMS8	10.000	5.168	6.000	1.961	1.079	0.400	0.490	0.184	0.231	0.516
	BetaMS15	8.000	4.000	4.000	1.707	0.822	0.250	0.414	0.396	0.222	0.442
	BetaMS17	10.000	2.000	2.000	1.471	0.500	0.000	0.320	1.000	1.000	0.337
	BetaMS23	10.000	2.000	2.000	2.000	0.693	0.000	0.500	1.000	1.000	0.526
	BetaMS25	10.000	2.000	2.000	1.923	0.673	0.400	0.480	0.167	0.222	0.505
	BetaMS28	10.000	1.000	1.000	1.000	0.000	0.000	0.000	N/A	N/A	0.000
	BetaMS40	10.000	4.596	5.000	2.703	1.235	0.900	0.630	–0.429	0.313	0.663
	BetaMS2.2	10.000	2.000	2.000	1.724	0.611	0.400	0.420	0.048	0.500	0.442
	BetaMS10.1	10.000	2.000	2.000	1.980	0.688	0.900	0.495	–0.818	0.057	0.521
	BetaMS14.1	10.000	4.568	5.000	2.941	1.257	0.200	0.660	0.697	0.250	0.695
	BetaMS14.2	10.000	4.568	5.000	2.410	1.158	0.800	0.585	–0.368	0.417	0.616
	Mean	9.769	3.122	3.308	2.010	0.812	0.439	0.464	0.489	0.387	0.530
	SE	0.166	1.319	0.444	0.147	0.098	0.097	0.047	0.050	0.298	0.102
SKN5 [8]	BetaMS4	2.000	1.000	1.000	1.000	0.000	0.000	0.000	N/A	N/A	0.000
	BetaMS5	3.000	1.333	2.000	1.385	0.451	0.333	0.278	–0.200	0.500	0.333
	BetaMS8	3.000	1.600	3.000	2.000	0.868	0.333	0.500	0.333	0.250	0.600
	BetaMS15	1.000	2.000	2.000	2.000	0.693	1.000	0.500	–1.000	0.333	1.000
	BetaMS17	3.000	1.533	2.000	1.800	0.637	0.000	0.444	1.000	1.000	0.533
	BetaMS23	3.000	1.800	3.000	3.000	1.099	0.000	0.667	1.000	0.500	0.800
	BetaMS25	3.000	1.000	1.000	1.000	0.000	0.000	0.000	N/A	N/A	0.000
	BetaMS28	3.000	1.000	1.000	1.000	0.000	0.000	0.000	N/A	N/A	0.000
	BetaMS40	3.000	1.800	4.000	3.000	1.242	1.000	0.667	–0.500	0.286	0.800
	BetaMS2.2	3.000	1.533	2.000	1.800	0.637	0.667	0.444	–0.500	0.500	0.533
	BetaMS10.1	3.000	1.333	2.000	1.385	0.451	0.333	0.278	–0.200	0.057	0.333
	BetaMS14.1	3.000	1.800	3.000	3.000	1.099	0.000	0.667	1.000	0.034	0.800
	BetaMS14.2	3.000	1.800	4.000	3.000	1.242	1.000	0.667	–0.500	0.333	0.800
	Mean	2.769	1.502	2.308	1.951	0.647	0.359	0.393	0.503	0.379	0.653
	SE	0.166	0.331	0.286	0.224	0.127	0.116	0.072	0.096	0.261	0.210
SKN6 [9]	BetaMS4	3.000	1.933	2.000	1.800	0.637	0.667	0.444	–0.500	0.400	0.533
	BetaMS5	3.000	1.933	2.000	1.800	0.637	0.000	0.444	1.000	0.500	0.533
	BetaMS8	3.000	3.600	5.000	4.500	1.561	1.000	0.778	–0.286	0.278	0.933
	BetaMS15	2.000	2.000	2.000	2.000	0.693	1.000	0.500	–1.000	0.111	0.667
	BetaMS17	3.000	2.800	3.000	3.000	1.099	0.000	0.667	1.000	0.750	0.800
	BetaMS23	3.000	1.000	1.000	1.000	0.000	0.000	0.000	N/A	N/A	0.000
	BetaMS25	3.000	1.933	2.000	1.800	0.637	0.667	0.444	–0.500	0.222	0.533

**Population**	**Locus**	**N**	***AR***	N_a	N_e	***I***	H_o	H_e	***F***	***M* ratio**	***PIC***
	BetaMS28	3.000	1.000	1.000	1.000	0.000	0.000	0.000	N/A	N/A	0.000
	BetaMS40	3.000	2.333	3.000	2.000	0.868	0.667	0.500	–0.333	0.125	0.600
	BetaMS2.2	3.000	1.667	2.000	1.385	0.451	0.333	0.278	–0.200	0.500	0.333
	BetaMS10.1	3.000	1.667	2.000	1.385	0.451	0.333	0.278	–0.200	0.057	0.333
	BetaMS14.1	3.000	2.333	3.000	2.000	0.868	0.333	0.500	0.333	0.038	0.600
	BetaMS14.2	3.000	3.000	4.000	3.000	1.242	1.000	0.667	–0.500	0.333	0.800
	Mean	2.923	2.092	2.462	2.051	0.703	0.462	0.423	0.513	0.301	0.606
	SE	0.077	0.710	0.312	0.267	0.123	0.110	0.065	0.079	0.212	0.179
SKN7 [10]	BetaMS4	5.000	1.000	1.000	1.000	0.000	0.000	0.000	N/A	N/A	0.000
	BetaMS5	5.000	1.000	1.000	1.000	0.000	0.000	0.000	N/A	N/A	0.000
	BetaMS8	5.000	1.378	3.000	1.515	0.639	0.400	0.340	–0.176	0.500	0.378
	BetaMS15	1.000	1.000	1.000	1.000	0.000	0.000	0.000	N/A	N/A	0.000
	BetaMS17	5.000	1.644	3.000	2.381	0.943	0.600	0.580	–0.034	0.167	0.644
	BetaMS23	5.000	1.000	1.000	1.000	0.000	0.000	0.000	N/A	N/A	0.000
	BetaMS25	5.000	1.467	2.000	1.724	0.611	0.200	0.420	0.524	0.222	0.467
	BetaMS28	4.000	1.000	1.000	1.000	0.000	0.000	0.000	N/A	N/A	0.000
	BetaMS40	5.000	1.667	5.000	2.500	1.228	0.800	0.600	–0.333	0.357	0.667
	BetaMS2.2	4.000	1.429	2.000	1.600	0.562	0.500	0.375	–0.333	0.500	0.429
	BetaMS10.1	4.000	1.000	1.000	1.000	0.000	0.000	0.000	N/A	N/A	0.000
	BetaMS14.1	5.000	1.556	2.000	2.000	0.693	0.200	0.500	0.600	0.167	0.556
	BetaMS14.2	5.000	1.600	3.000	2.174	0.898	0.400	0.540	0.259	0.500	0.600
	Mean	4.462	1.288	2.000	1.530	0.429	0.238	0.258	0.288	0.345	0.534
	SE	0.312	0.277	0.340	0.161	0.124	0.076	0.072	0.080	0.147	0.103

Sample size (N); allelic richness (*AR*); number of alleles (N_a); number of effective alleles (N_e); Shannon's information index (*I*); observed heterozygosity (H_o); expected heterozygosity (H_e); fixation index (*F*); ratio of number of allele to allele range (*M* ratio); polymorphic information content (*PIC*).

[1] SPK = Samut Prakan. [2] BKK = Bangkok 1. [3] BKK2 = Bangkok 2. [4] SKN1 = Samut Sakhon 1. [5] SKN2 = Samut Sakhon 2. [6] SKN3 = Samut Sakhon 3. [7] SKN4 = Samut Sakhon 4. [8] SKN5 = Samut Sakhon 5. [9] SKN6 = Samut Sakhon 6. [10] SKN7 = Samut Sakhon 7.

Table S5. Comparison of genetic diversity parameters between Mahachai betta (*Betta mahachaiensis*) individuals based on 13 microsatellite loci.

	Population 1	Population 2	df	SE	t-test	*p*-value
Heterozygosity (H_o)	SPK [1]	BKK1 [2]	–0.001	0.131	–0.008	0.994
	SPK [1]	BKK2 [3]	0.006	0.152	0.039	0.970
	SPK [1]	SKN1 [4]	0.019	0.145	0.131	0.899
	SPK [1]	SKN2 [5]	–0.060	0.129	–0.464	0.646
	SPK [1]	SKN3 [6]	–0.028	0.147	–0.190	0.853
	SPK [1]	SKN4 [7]	–0.125	0.133	–0.940	0.357
	SPK [1]	SKN5 [8]	–0.045	0.147	–0.305	0.773
	SPK [1]	SKN6 [9]	–0.148	0.143	–1.037	0.344
	SPK [1]	SKN7 [10]	0.076	0.119	0.641	0.531
	BKK1 [2]	BKK2 [3]	0.007	0.154	0.045	0.966
	BKK1 [2]	SKN1 [4]	0.020	0.147	0.136	0.895
	BKK1 [2]	SKN2 [5]	–0.059	0.132	–0.449	0.657
	BKK1 [2]	SKN3 [6]	–0.027	0.149	–0.181	0.860
	BKK1 [2]	SKN4 [7]	–0.124	0.135	–0.918	0.370
	BKK1 [2]	SKN5 [8]	–0.044	0.149	–0.295	0.780
	BKK1 [2]	SKN6 [9]	–0.147	0.145	–1.016	0.353
	BKK1 [2]	SKN7 [10]	0.077	0.121	0.637	0.535
	BKK2 [3]	SKN1 [4]	0.013	0.166	0.078	0.941
	BKK2 [3]	SKN2 [5]	–0.066	0.153	–0.432	0.685
	BKK2 [3]	SKN3 [6]	–0.034	0.168	–0.202	0.848
	BKK2 [3]	SKN4 [7]	–0.131	0.156	–0.840	0.440
	BKK2 [3]	SKN5 [8]	–0.051	0.168	–0.303	0.777
	BKK2 [3]	SKN6 [9]	–0.154	0.164	–0.937	0.402
	BKK2 [3]	SKN7 [10]	0.070	0.144	0.487	0.655
	SKN1 [4]	SKN2	–0.079	0.146	–0.542	0.603
	SKN1 [4]	SKN3 [6]	–0.047	0.162	–0.290	0.780
	SKN1 [4]	SKN4 [7]	–0.144	0.149	–0.967	0.363
	SKN1 [4]	SKN5 [8]	–0.064	0.162	–0.395	0.710
	SKN1 [4]	SKN6 [9]	–0.167	0.158	–1.059	0.339
	SKN1 [4]	SKN7 [10]	0.057	0.136	0.419	0.691
	SKN2 [5]	SKN3 [6]	0.032	0.148	0.216	0.833
	SKN2 [5]	SKN4 [7]	–0.065	0.134	–0.486	0.631
	SKN2 [5]	SKN5 [8]	0.015	0.148	0.101	0.923
	SKN2 [5]	SKN6 [9]	–0.088	0.143	–0.614	0.564

	Population 1	Population 2	df	SE	t-test	*p*-value
	SKN2 [5]	SKN7 [10]	0.136	0.119	1.140	0.270
	SKN3 [6]	SKN4 [7]	–0.097	0.151	–0.641	0.536
	SKN3 [6]	SKN5 [8]	–0.017	0.164	–0.104	0.921
	SKN3 [6]	SKN6 [9]	–0.120	0.160	–0.751	0.484
	SKN3 [6]	SKN7 [10]	0.104	0.139	0.750	0.478
	SKN4 [7]	SKN5 [8]	0.080	0.151	0.529	0.619
	SKN4 [7]	SKN6 [9]	–0.023	0.147	–0.157	0.881
	SKN4 [7]	SKN7 [10]	0.201	0.123	1.631	0.127
	SKN5 [8]	SKN6 [9]	–0.103	0.160	–0.644	0.555
	SKN5 [8]	SKN7 [10]	0.121	0.139	0.873	0.435
	SKN6 [9]	SKN7 [10]	0.224	0.134	1.675	0.171
Heterozygosity (H_e)	SPK [1]	BKK1 [2]	0.001	0.098	0.010	0.992
	SPK [1]	BKK2 [3]	0.180	0.102	1.762	0.133
	SPK [1]	SKN1 [4]	0.120	0.099	1.210	0.258
	SPK [1]	SKN2 [5]	–0.044	0.093	–0.471	0.640
	SPK [1]	SKN3 [6]	0.141	0.095	1.477	0.165
	SPK [1]	SKN4 [7]	–0.083	0.081	–1.024	0.315
	SPK [1]	SKN5 [8]	–0.012	0.098	–0.123	0.906
	SPK [1]	SKN6 [9]	–0.042	0.093	–0.453	0.663
	SPK [1]	SKN7 [10]	0.123	0.098	1.259	0.233
	BKK1 [2]	BKK2 [3]	0.179	0.106	1.686	0.143
	BKK1 [2]	SKN1 [4]	0.119	0.103	1.153	0.279
	BKK1 [2]	SKN2 [5]	–0.045	0.098	–0.461	0.649
	BKK1 [2]	SKN3 [6]	0.140	0.100	1.404	0.186
	BKK1 [2]	SKN4 [7]	–0.084	0.086	–0.977	0.342
	BKK1 [2]	SKN5 [8]	–0.013	0.102	–0.128	0.902
	BKK1 [2]	SKN6 [9]	–0.043	0.097	–0.443	0.670
	BKK1 [2]	SKN7 [10]	0.122	0.102	1.198	0.255
	BKK2 [3]	SKN1 [4]	–0.060	0.108	–0.558	0.602
	BKK2 [3]	SKN2 [5]	–0.224	0.102	–2.192	0.074
	BKK2 [3]	SKN3 [6]	–0.039	0.104	–0.374	0.724
	BKK2 [3]	SKN4 [7]	–0.263	0.091	–2.888	0.051
	BKK2 [3]	SKN5 [8]	–0.192	0.106	–1.809	0.145
	BKK2 [3]	SKN6 [9]	–0.222	0.102	–2.186	0.096
	BKK2 [3]	SKN7 [10]	–0.057	0.106	–0.537	0.614

Population 1	Population 2	df	SE	t-test	*p*-value
SKN1 [4]	SKN2	–0.164	0.099	–1.654	0.133
SKN1 [4]	SKN3 [6]	0.021	0.101	0.208	0.842
SKN1 [4]	SKN4 [7]	–0.203	0.088	–2.316	0.063
SKN1 [4]	SKN5 [8]	–0.132	0.103	–1.278	0.259
SKN1 [4]	SKN6 [9]	–0.162	0.098	–1.645	0.161
SKN1 [4]	SKN7 [10]	0.003	0.103	0.029	0.978
SKN2 [5]	SKN3 [6]	0.185	0.095	1.938	0.076
SKN2 [5]	SKN4 [7]	–0.039	0.081	–0.481	0.634
SKN2 [5]	SKN5 [8]	0.032	0.098	0.328	0.754
SKN2 [5]	SKN6 [9]	0.002	0.093	0.022	0.983
SKN2 [5]	SKN7 [10]	0.167	0.098	1.710	0.113
SKN3 [6]	SKN4 [7]	–0.224	0.083	–2.683	0.028
SKN3 [6]	SKN5 [8]	–0.153	0.100	–1.534	0.184
SKN3 [6]	SKN6 [9]	–0.183	0.095	–1.930	0.106
SKN3 [6]	SKN7 [10]	–0.018	0.100	–0.180	0.861
SKN4 [7]	SKN5 [8]	0.071	0.086	0.826	0.456
SKN4 [7]	SKN6 [9]	0.041	0.080	0.511	0.634
SKN4 [7]	SKN7 [10]	0.206	0.086	2.396	0.045
SKN5 [8]	SKN6 [9]	–0.030	0.097	–0.309	0.773
SKN5 [8]	SKN7 [10]	0.135	0.102	1.326	0.239
SKN6 [9]	SKN7 [10]	0.165	0.097	1.701	0.143

df = Difference of means

[1] SPK = Samut Prakan. [2] BKK = Bangkok 1. [3] BKK2 = Bangkok 2. [4] SKN1 = Samut Sakhon 1. [5] SKN2 = Samut Sakhon 2. [6] SKN3 = Samut Sakhon 3. [7] SKN4 = Samut Sakhon 4. [8] SKN5 = Samut Sakhon 5. [9] SKN6 = Samut Sakhon 6. [10] SKN7 = Samut Sakhon 7.

Table S6. Hardy–Weinberg and linkage disequilibrium analysis of alleles of 13 microsatellites of Mahachai betta (*Betta mahachaiensis*) individuals of SPK.

Locus	Number of Genotypes	Observed Heterozygosity	Expected Heterozygosity	*p*-value	SD	Number of linked loci	Polymorphic Locus
BetaMS4	15	0.200	0.467	0.012	0.000	2	1
BetaMS5	17	0.824	0.631	0.817	0.000	1	1
BetaMS8	17	0.176	0.166	1.000	0.000	0	1
BetaMS15	17	0.059	0.116	0.029	0.000	1	1
BetaMS17	17	0.000	0.392	0.000	0.000	2	1
BetaMS23	16	0.000	0.516	0.000	0.000	1	1
BetaMS25	17	0.471	0.513	1.000	0.000	0	1
BetaMS28	N/A	N/A	N/A	N/A	N/A	0	0
BetaMS40	17	0.941	0.656	0.140	0.000	2	1
BetaMS2.2	17	0.706	0.756	0.159	0.000	1	1
BetaMS10.1	17	0.059	0.059	1.000	0.000	0	1
BetaMS14.1	17	0.353	0.562	0.002	0.000	4	1
BetaMS14.2	17	0.294	0.271	1.000	0.000	2	1

Table S7. Hardy–Weinberg and linkage disequilibrium analysis of alleles of 13 microsatellites of Mahachai betta (*Betta mahachaiensis*) individuals of BKK1.

Locus	Number of Genotypes	Observed Heterozygosity	Expected Heterozygosity	*p*-value	SD	Number of linked loci	Polymorphic Locus
BetaMS4	6	0.167	0.712	0.013	0.000	1	1
BetaMS5	11	0.636	0.584	0.602	0.000	2	1
BetaMS8	11	0.091	0.091	1.000	0.000	0	1
BetaMS15	11	0.091	0.091	1.000	0.000	1	1
BetaMS17	11	0.000	0.450	0.000	0.000	2	1
BetaMS23	10	0.000	0.189	0.053	0.000	1	1
BetaMS25	11	0.182	0.312	0.279	0.000	1	1
BetaMS28	N/A	N/A	N/A	N/A	N/A	0	0
BetaMS40	10	1.000	0.737	0.749	0.000	2	1
BetaMS2.2	10	0.200	0.195	1.000	0.000	1	1
BetaMS10.1	11	0.636	0.455	0.480	0.001	0	1
BetaMS14.1	11	0.273	0.805	0.001	0.000	1	1
BetaMS14.2	11	0.818	0.584	0.306	0.000	0	1

Table S8. Hardy–Weinberg and linkage disequilibrium analysis of alleles of 13 microsatellites of Mahachai betta (*Betta mahachaiensis*) individuals of BKK2.

Locus	Number of Genotypes	Observed Heterozygosity	Expected Heterozygosity	*p*-value	SD	Number of linked loci	Polymorphic Locus
BetaMS4	N/A	N/A	N/A	N/A	N/A	0	0
BetaMS5	3	0.667	0.600	1.000	0.000	0	1
BetaMS8	N/A	N/A	N/A	N/A	N/A	0	0
BetaMS15	3	0.333	0.333	1.000	0.000	0	1
BetaMS17	N/A	N/A	N/A	N/A	N/A	0	0
BetaMS23	N/A	N/A	N/A	N/A	N/A	0	0
BetaMS25	N/A	N/A	N/A	N/A	N/A	0	0
BetaMS28	N/A	N/A	N/A	N/A	N/A	0	0
BetaMS40	3	1.000	0.800	1.000	0.000	0	1
BetaMS2.2	N/A	N/A	N/A	N/A	N/A	0	0
BetaMS10.1	3	1.000	0.600	0.400	0.000	0	1
BetaMS14.1	N/A	N/A	N/A	N/A	N/A	0	0
BetaMS14.2	3	1.000	0.800	1.000	0.000	0	1

Table S9. Hardy–Weinberg and linkage disequilibrium analysis of alleles of 13 microsatellites of Mahachai betta (*Betta mahachaiensis*) individuals of SPK1.

Locus	Number of Genotypes	Observed Heterozygosity	Expected Heterozygosity	*p*-value	SD	Number of linked loci	Polymorphic Locus
BetaMS4	N/A	N/A	N/A	N/A	N/A	0	0
BetaMS5	4	1.000	0.679	0.316	0.000	0	1
BetaMS8	N/A	N/A	N/A	N/A	N/A	0	0
BetaMS15	N/A	N/A	N/A	N/A	N/A	0	0
BetaMS17	4	0.000	0.429	0.143	0.000	0	1
BetaMS23	N/A	N/A	N/A	N/A	N/A	0	0
BetaMS25	3	0.667	0.533	1.000	0.000	0	1
BetaMS28	N/A	N/A	N/A	N/A	N/A	0	0
BetaMS40	4	1.000	0.714	0.543	0.000	0	1
BetaMS2.2	N/A	N/A	N/A	N/A	N/A	0	0
BetaMS10.1	3	0.667	0.733	1.000	0.000	0	1
BetaMS14.1	4	0.000	0.429	0.142	0.000	0	1
BetaMS14.2	4	0.500	0.429	1.000	0.000	0	1

Table S10. Hardy–Weinberg and linkage disequilibrium analysis of alleles of 13 microsatellites of Mahachai betta (*Betta mahachaiensis*) individuals of SPK2.

Locus	Number of Genotypes	Observed Heterozygosity	Expected Heterozygosity	*p*-value	SD	Number of linked loci	Polymorphic Locus
BetaMS4	17	0.471	0.492	0.250	0.000	1	1
BetaMS5	20	0.900	0.586	0.009	0.000	2	1
BetaMS8	20	0.200	0.192	1.000	0.000	1	1
BetaMS15	16	0.188	0.179	1.000	0.000	2	1
BetaMS17	20	0.000	0.595	0.000	0.000	3	1
BetaMS23	19	0.000	0.398	0.000	0.000	1	1
BetaMS25	20	0.500	0.508	1.000	0.000	0	1
BetaMS28	N/A	N/A	N/A	N/A	N/A	0	0
BetaMS40	20	1.000	0.722	0.290	0.000	1	1
BetaMS2.2	20	0.200	0.185	1.000	0.000	0	1
BetaMS10.1	20	0.650	0.499	0.123	0.000	0	1
BetaMS14.1	20	0.250	0.882	0.000	0.000	2	1
BetaMS14.2	20	0.500	0.435	1.000	0.000	1	1

Table S11. Hardy–Weinberg and linkage disequilibrium analysis of alleles of 13 microsatellites of Mahachai betta (*Betta mahachaiensis*) individuals of SPK3.

Locus	Number of Genotypes	Observed Heterozygosity	Expected Heterozygosity	*p*-value	SD	Number of linked loci	Polymorphic Locus
BetaMS4	4	0.250	0.250	1.000	0.000	0	1
BetaMS5	5	0.600	0.511	1.000	0.000	0	1
BetaMS8	N/A	N/A	N/A	N/A	N/A	0	0
BetaMS15	N/A	N/A	N/A	N/A	N/A	0	0
BetaMS17	N/A	N/A	N/A	N/A	N/A	0	0
BetaMS23	N/A	N/A	N/A	N/A	N/A	0	0
BetaMS25	5	1.000	0.556	0.127	0.000	0	1
BetaMS28	N/A	N/A	N/A	N/A	N/A	0	0
BetaMS40	5	1.000	0.711	0.427	0.000	0	1
BetaMS2.2	N/A	N/A	N/A	N/A	N/A	0	0
BetaMS10.1	5	1.000	0.556	0.128	0.000	0	1
BetaMS14.1	5	0.200	0.511	0.111	0.000	0	1
BetaMS14.2	5	0.400	0.378	1.000	0.000	0	1

Table S12. Hardy–Weinberg and linkage disequilibrium analysis of alleles of 13 microsatellites of Mahachai betta (*Betta mahachaiensis*) individuals of SPK4.

Locus	Number of Genotypes	Observed Heterozygosity	Expected Heterozygosity	*p*-value	SD	Number of linked loci	Polymorphic Locus
BetaMS4	9	0.556	0.451	1.000	0.000	0	1
BetaMS5	10	0.900	0.642	0.351	0.001	1	1
BetaMS8	10	0.400	0.516	0.139	0.000	1	1
BetaMS15	8	0.250	0.442	0.016	0.000	2	1
BetaMS17	10	0.000	0.337	0.009	0.000	1	1
BetaMS23	10	0.000	0.526	0.001	0.000	0	1
BetaMS25	10	0.400	0.505	0.572	0.000	1	1
BetaMS28	N/A	N/A	N/A	N/A	N/A	0	0
BetaMS40	10	0.900	0.663	0.683	0.000	1	1
BetaMS2.2	10	0.400	0.442	1.000	0.000	0	1
BetaMS10.1	10	0.900	0.521	0.045	0.000	0	1
BetaMS14.1	10	0.200	0.695	0.000	0.000	1	1
BetaMS14.2	10	0.800	0.616	0.876	0.000	0	1

Table S13. Hardy–Weinberg and linkage disequilibrium analysis of alleles of 13 microsatellites of Mahachai betta (*Betta mahachaiensis*) individuals of SPK5.

Locus	Number of Genotypes	Observed Heterozygosity	Expected Heterozygosity	*p*-value	SD	Number of linked loci	Polymorphic Locus
BetaMS4	N/A	N/A	N/A	N/A	N/A	0	0
BetaMS5	3	0.333	0.333	1.000	0.000	1	1
BetaMS8	3	0.333	0.600	0.198	0.000	1	1
BetaMS15	1	1.000	1.000	1.000	0.000	1	1
BetaMS17	3	0.000	0.533	0.199	0.000	0	1
BetaMS23	3	0.000	0.800	0.067	0.000	0	1
BetaMS25	N/A	N/A	N/A	N/A	N/A	0	0
BetaMS28	N/A	N/A	N/A	N/A	N/A	0	0
BetaMS40	3	1.000	0.800	1.000	0.000	0	1
BetaMS2.2	3	0.667	0.533	1.000	0.000	1	1
BetaMS10.1	3	0.333	0.333	1.000	0.000	0	1
BetaMS14.1	3	0.000	0.800	0.068	0.000	0	1
BetaMS14.2	3	1.000	0.800	1.000	0.000	0	1

Table S14. Hardy–Weinberg and linkage disequilibrium analysis of alleles of 13 microsatellites of Mahachai betta (*Betta mahachaiensis*) individuals of SPK6.

Locus	Number of Genotypes	Observed Heterozygosity	Expected Heterozygosity	*p*-value	SD	Number of linked loci	Polymorphic Locus
BetaMS4	3	0.667	0.533	1.000	0.000	0	1
BetaMS5	3	0.000	0.533	0.200	0.000	0	1
BetaMS8	3	1.000	0.933	1.000	0.000	0	1
BetaMS15	2	1.000	0.667	1.000	0.000	0	1
BetaMS17	3	0.000	0.800	0.066	0.000	0	1
BetaMS23	N/A	N/A	N/A	N/A	N/A	0	0
BetaMS25	3	0.667	0.533	1.000	0.000	0	1
BetaMS28	N/A	N/A	N/A	N/A	N/A	0	0
BetaMS40	3	0.667	0.600	1.000	0.000	0	1
BetaMS2.2	3	0.333	0.333	1.000	0.000	0	1
BetaMS10.1	3	0.333	0.333	1.000	0.000	0	1
BetaMS14.1	3	0.333	0.600	0.200	0.000	0	1
BetaMS14.2	3	1.000	0.800	1.000	0.000	0	1

Table S15. Hardy–Weinberg and linkage disequilibrium analysis of alleles of 13 microsatellites of Mahachai betta (*Betta mahachaiensis*) individuals of SPK7.

Locus	Number of Genotypes	Observed Heterozygosity	Expected Heterozygosity	*p*-value	SD	Number of linked loci	Polymorphic Locus
BetaMS4	N/A	N/A	N/A	N/A	N/A	0	0
BetaMS5	N/A	N/A	N/A	N/A	N/A	0	0
BetaMS8	5	0.400	0.378	1.000	0.000	0	1
BetaMS15	N/A	N/A	N/A	N/A	N/A	0	0
BetaMS17	5	0.600	0.644	1.000	0.000	0	1
BetaMS23	N/A	N/A	N/A	N/A	N/A	0	0
BetaMS25	5	0.200	0.467	0.334	0.000	0	1
BetaMS28	N/A	N/A	N/A	N/A	N/A	0	0
BetaMS40	5	0.800	0.667	1.000	0.000	0	1
BetaMS2.2	4	0.500	0.429	1.000	0.000	0	1
BetaMS10.1	N/A	N/A	N/A	N/A	N/A	0	0
BetaMS14.1	5	0.200	0.556	0.366	0.000	0	1
BetaMS14.2	5	0.400	0.600	0.620	0.000	0	1

Table S16. Inbreeding coefficients, relatedness, effective population size and ratio of effective population size to sample size (N_e/N) of 81 Mahachai betta (*Betta mahachaiensis*) individuals.

Population	N	F_{IS}	Relatedness (*r*)	Estimated N_e	95% CIs for N_e	N_e/N
SPK[1]	17	–0.157±0.045	–0.035±0.083	48.500	12.300 – 13.000	2.853
BKK1[2]	11	–0.207±0.090	–0.061±0.090	Infinite	Infinite	Infinite
BKK2[3]	3	–0.756±0.175	–0.158±0.013	Infinite	Infinite	Infinite
SKN1[4]	4	–0.586±0.049	–0.158±0.096	Infinite	Infinite	Infinite
SKN2[5]	20	–0.134±0.057	–0.030±0.065	Infinite	Infinite	Infinite
SKN3[6]	5	–0.650±0.113	–0.102±0.094	Infinite	Infinite	Infinite
SKN4[7]	10	–0.236±0.104	–0.058±0.088	38.100	7.400	3.810
SKN5[8]	3	–0.256±0.056	–0.283±0.086	Infinite	Infinite	Infinite
SKN6[9]	3	–0.391±0.178	–0.242±0.106	Infinite	Infinite	Infinite
SKN7[10]	5	–0.376±0.145	–0.128±0.104	91.100	0.900	18.220

Estimates were calculated using NeEstimator version 2.1 (Do et al., 2013), COANCESTRY version 1.0.1.9 (Wang, 2011), and GenAlEx version 6.5 (Peakall & Smouse, 2012). Detailed information for all Mahachai betta individuals is presented in Table S1. Sample size (N); inbreeding coefficient (F_{IS}); effective population size (N_e).

[1] SPK = Samut Prakan. [2] BKK = Bangkok 1. [3] BKK2 = Bangkok 2. [4] SKN1 = Samut Sakhon 1. [5] SKN2 = Samut Sakhon 2. [6] SKN3 = Samut Sakhon 3. [7] SKN4 = Samut Sakhon 4. [8] SKN5 = Samut Sakhon 5. [9] SKN6 = Samut Sakhon 6. [10] SKN7 = Samut Sakhon 7.

Table S17. Pairwise genetic relatedness (*r*) for all 17 Mahachai betta (*Betta mahachaiensis*) individuals in SPK.

Sample 1	Sample 2	*r*
SPK-1	SPK-2	0.051
SPK-1	SPK-3	–0.110
SPK-2	SPK-3	–0.010
SPK-1	SPK-4	–0.117
SPK-2	SPK-4	–0.025
SPK-3	SPK-4	–0.181
SPK-1	SPK-5	–0.059
SPK-2	SPK-5	0.071
SPK-3	SPK-5	–0.189
SPK-4	SPK-5	–0.033
SPK-1	SPK-6	–0.088
SPK-2	SPK-6	–0.138
SPK-3	SPK-6	0.121
SPK-4	SPK-6	0.025
SPK-5	SPK-6	–0.089
SPK-1	SPK-7	–0.090
SPK-2	SPK-7	0.013
SPK-3	SPK-7	–0.120
SPK-4	SPK-7	0.017
SPK-5	SPK-7	0.030
SPK-6	SPK-7	0.022
SPK-1	SPK-8	0.151
SPK-2	SPK-8	–0.021
SPK-3	SPK-8	–0.086
SPK-4	SPK-8	–0.140
SPK-5	SPK-8	–0.083
SPK-6	SPK-8	–0.065
SPK-7	SPK-8	0.076
SPK-1	SPK-9	–0.003
SPK-2	SPK-9	–0.055
SPK-3	SPK-9	0.026
SPK-4	SPK-9	–0.030
SPK-5	SPK-9	–0.038
SPK-6	SPK-9	0.156
SPK-7	SPK-9	–0.082
SPK-8	SPK-9	–0.003
SPK-1	SPK-10	0.013
SPK-2	SPK-10	0.001
SPK-3	SPK-10	–0.095
SPK-4	SPK-10	0.015
SPK-5	SPK-10	–0.027
SPK-6	SPK-10	–0.073
SPK-7	SPK-10	–0.079
SPK-8	SPK-10	0.038
SPK-9	SPK-10	0.065
SPK-1	SPK-11	–0.024
SPK-2	SPK-11	–0.184
SPK-3	SPK-11	–0.067
SPK-4	SPK-11	–0.040
SPK-5	SPK-11	–0.103
SPK-6	SPK-11	–0.065
SPK-7	SPK-11	0.103

Sample 1	Sample 2	*r*
SPK-8	SPK-11	0.000
SPK-9	SPK-11	–0.011
SPK-10	SPK-11	–0.005
SPK-1	SPK-12	0.081
SPK-2	SPK-12	–0.023
SPK-3	SPK-12	–0.088
SPK-4	SPK-12	–0.087
SPK-5	SPK-12	–0.085
SPK-6	SPK-12	–0.078
SPK-7	SPK-12	–0.101
SPK-8	SPK-12	0.104
SPK-9	SPK-12	–0.042
SPK-10	SPK-12	0.035
SPK-11	SPK-12	–0.015
SPK-1	SPK-13	–0.049
SPK-2	SPK-13	0.066
SPK-3	SPK-13	–0.107
SPK-4	SPK-13	–0.089
SPK-5	SPK-13	0.085
SPK-6	SPK-13	–0.174
SPK-7	SPK-13	–0.046
SPK-8	SPK-13	–0.026
SPK-9	SPK-13	–0.109
SPK-10	SPK-13	0.032
SPK-11	SPK-13	–0.155
SPK-12	SPK-13	0.100
SPK-1	SPK-14	–0.083
SPK-2	SPK-14	0.119
SPK-3	SPK-14	–0.173
SPK-4	SPK-14	0.073
SPK-5	SPK-14	0.104
SPK-6	SPK-14	–0.012
SPK-7	SPK-14	–0.082
SPK-8	SPK-14	–0.154
SPK-9	SPK-14	–0.019
SPK-10	SPK-14	0.001
SPK-11	SPK-14	–0.119
SPK-12	SPK-14	–0.028
SPK-13	SPK-14	0.094
SPK-1	SPK-15	–0.011
SPK-2	SPK-15	–0.074
SPK-3	SPK-15	0.065
SPK-4	SPK-15	–0.099
SPK-5	SPK-15	–0.156
SPK-6	SPK-15	0.040
SPK-7	SPK-15	–0.246
SPK-8	SPK-15	–0.011
SPK-9	SPK-15	–0.012
SPK-10	SPK-15	–0.030
SPK-11	SPK-15	–0.059
SPK-12	SPK-15	0.070
SPK-13	SPK-15	–0.059
SPK-14	SPK-15	–0.112
SPK-1	SPK-16	0.041
SPK-2	SPK-16	–0.008

Sample 1	Sample 2	r
SPK-3	SPK-16	–0.043
SPK-4	SPK-16	–0.049
SPK-5	SPK-16	–0.102
SPK-6	SPK-16	0.054
SPK-7	SPK-16	–0.119
SPK-8	SPK-16	0.065
SPK-9	SPK-16	–0.050
SPK-10	SPK-16	0.027
SPK-11	SPK-16	–0.045
SPK-12	SPK-16	0.085
SPK-13	SPK-16	–0.011
SPK-14	SPK-16	0.017
SPK-15	SPK-16	0.038
SPK-1	SPK-17	–0.021
SPK-2	SPK-17	–0.106
SPK-3	SPK-17	–0.215
SPK-4	SPK-17	–0.029
SPK-5	SPK-17	0.052
SPK-6	SPK-17	–0.115
SPK-7	SPK-17	0.102
SPK-8	SPK-17	–0.162
SPK-9	SPK-17	–0.064
SPK-10	SPK-17	–0.077
SPK-11	SPK-17	0.072
SPK-12	SPK-17	–0.164
SPK-13	SPK-17	–0.156
SPK-14	SPK-17	–0.041
SPK-15	SPK-17	–0.192
SPK-16	SPK-17	–0.119

Table S18. Pairwise genetic relatedness (*r*) for all 11 Mahachai betta (*Betta mahachaiensis*) individuals in BKK1.

Sample 1	Sample 2	*r*
BKK1-1	BKK1-2	–0.131
BKK1-1	BKK1-3	–0.170
BKK1-2	BKK1-3	0.139
BKK1-1	BKK1-4	–0.148
BKK1-2	BKK1-4	–0.081
BKK1-3	BKK1-4	–0.029
BKK1-1	BKK1-5	–0.167
BKK1-2	BKK1-5	–0.064
BKK1-3	BKK1-5	–0.134
BKK1-4	BKK1-5	–0.030
BKK1-1	BKK1-6	–0.070
BKK1-2	BKK1-6	–0.158
BKK1-3	BKK1-6	–0.075
BKK1-4	BKK1-6	–0.094
BKK1-5	BKK1-6	–0.006
BKK1-1	BKK1-7	–0.043
BKK1-2	BKK1-7	–0.020
BKK1-3	BKK1-7	–0.129
BKK1-4	BKK1-7	–0.152
BKK1-5	BKK1-7	–0.013
BKK1-6	BKK1-7	–0.013
BKK1-1	BKK1-8	–0.180
BKK1-2	BKK1-8	–0.154
BKK1-3	BKK1-8	–0.100
BKK1-4	BKK1-8	–0.033
BKK1-5	BKK1-8	0.026
BKK1-6	BKK1-8	0.063
BKK1-7	BKK1-8	–0.168
BKK1-1	BKK1-9	–0.121
BKK1-2	BKK1-9	–0.095
BKK1-3	BKK1-9	0.055
BKK1-4	BKK1-9	0.079
BKK1-5	BKK1-9	0.017
BKK1-6	BKK1-9	–0.063
BKK1-7	BKK1-9	–0.083
BKK1-8	BKK1-9	–0.086
BKK1-1	BKK1-10	–0.197
BKK1-2	BKK1-10	0.085
BKK1-3	BKK1-10	0.080
BKK1-4	BKK1-10	0.050
BKK1-5	BKK1-10	–0.050
BKK1-6	BKK1-10	–0.117
BKK1-7	BKK1-10	–0.249
BKK1-8	BKK1-10	–0.045
BKK1-9	BKK1-10	–0.095
BKK1-1	BKK1-11	0.217
BKK1-2	BKK1-11	–0.133
BKK1-3	BKK1-11	–0.123
BKK1-4	BKK1-11	0.041
BKK1-5	BKK1-11	–0.032
BKK1-6	BKK1-11	–0.086
BKK1-7	BKK1-11	–0.119

Sample 1	Sample 2	*r*
BKK1-8	BKK1-11	–0.071
BKK1-9	BKK1-11	–0.065
BKK1-10	BKK1-11	–0.023

Table S19. Pairwise genetic relatedness (*r*) for all 3 Mahachai betta (*Betta mahachaiensis*) individuals in BKK2.

Sample 1	Sample 2	*r*
BKK2-1	BKK2-2	–0.165
BKK2-1	BKK2-3	–0.143
BKK2-2	BKK2-3	–0.165

Table S20. Pairwise genetic relatedness (*r*) for all 4 Mahachai betta (*Betta mahachaiensis*) individuals in SKN1.

Sample 1	Sample 2	*r*
SKN1-1	SKN1-2	–0.121
SKN1-1	SKN1-3	–0.073
SKN1-2	SKN1-3	–0.115
SKN1-1	SKN1-4	–0.247
SKN1-2	SKN1-4	–0.222
SKN1-3	SKN1-4	–0.095

Table S21. Pairwise genetic relatedness (*r*) for all 20 Mahachai betta (*Betta mahachaiensis*) individuals in SKN2.

Sample 1	Sample 2	*r*
SKN2-1	SKN2-2	0.081
SKN2-1	SKN2-3	0.086
SKN2-2	SKN2-3	0.101
SKN2-1	SKN2-4	–0.083
SKN2-2	SKN2-4	–0.055
SKN2-3	SKN2-4	–0.035
SKN2-1	SKN2-5	–0.045
SKN2-2	SKN2-5	–0.030
SKN2-3	SKN2-5	0.068
SKN2-4	SKN2-5	–0.046
SKN2-1	SKN2-6	–0.094
SKN2-2	SKN2-6	–0.093
SKN2-3	SKN2-6	–0.053
SKN2-4	SKN2-6	–0.011
SKN2-5	SKN2-6	0.087
SKN2-1	SKN2-7	–0.093
SKN2-2	SKN2-7	0.006
SKN2-3	SKN2-7	–0.054
SKN2-4	SKN2-7	0.151
SKN2-5	SKN2-7	–0.113
SKN2-6	SKN2-7	0.026
SKN2-1	SKN2-8	0.079
SKN2-2	SKN2-8	0.116
SKN2-3	SKN2-8	0.111
SKN2-4	SKN2-8	–0.058
SKN2-5	SKN2-8	0.011
SKN2-6	SKN2-8	–0.061
SKN2-7	SKN2-8	–0.036
SKN2-1	SKN2-9	–0.103
SKN2-2	SKN2-9	–0.031
SKN2-3	SKN2-9	–0.063
SKN2-4	SKN2-9	–0.043
SKN2-5	SKN2-9	–0.125
SKN2-6	SKN2-9	–0.054
SKN2-7	SKN2-9	0.178
SKN2-8	SKN2-9	0.060
SKN2-1	SKN2-10	–0.090
SKN2-2	SKN2-10	–0.084
SKN2-3	SKN2-10	–0.064
SKN2-4	SKN2-10	0.000
SKN2-5	SKN2-10	–0.010
SKN2-6	SKN2-10	–0.011
SKN2-7	SKN2-10	–0.064
SKN2-8	SKN2-10	–0.051
SKN2-9	SKN2-10	–0.074
SKN2-1	SKN2-11	–0.039
SKN2-2	SKN2-11	–0.118
SKN2-3	SKN2-11	–0.087
SKN2-4	SKN2-11	–0.019
SKN2-5	SKN2-11	–0.039
SKN2-6	SKN2-11	–0.030
SKN2-7	SKN2-11	–0.086

Sample 1	Sample 2	*r*
SKN2-8	SKN2-11	–0.066
SKN2-9	SKN2-11	0.039
SKN2-10	SKN2-11	0.018
SKN2-1	SKN2-12	–0.120
SKN2-2	SKN2-12	0.045
SKN2-3	SKN2-12	–0.110
SKN2-4	SKN2-12	–0.009
SKN2-5	SKN2-12	–0.071
SKN2-6	SKN2-12	0.014
SKN2-7	SKN2-12	0.006
SKN2-8	SKN2-12	–0.096
SKN2-9	SKN2-12	–0.082
SKN2-10	SKN2-12	–0.023
SKN2-11	SKN2-12	–0.037
SKN2-1	SKN2-13	–0.060
SKN2-2	SKN2-13	–0.157
SKN2-3	SKN2-13	–0.106
SKN2-4	SKN2-13	0.032
SKN2-5	SKN2-13	–0.055
SKN2-6	SKN2-13	–0.041
SKN2-7	SKN2-13	–0.032
SKN2-8	SKN2-13	–0.107
SKN2-9	SKN2-13	–0.024
SKN2-10	SKN2-13	–0.026
SKN2-11	SKN2-13	0.035
SKN2-12	SKN2-13	–0.066
SKN2-1	SKN2-14	–0.067
SKN2-2	SKN2-14	0.077
SKN2-3	SKN2-14	0.017
SKN2-4	SKN2-14	–0.090
SKN2-5	SKN2-14	–0.067
SKN2-6	SKN2-14	–0.135
SKN2-7	SKN2-14	–0.101
SKN2-8	SKN2-14	0.032
SKN2-9	SKN2-14	–0.036
SKN2-10	SKN2-14	–0.133
SKN2-11	SKN2-14	–0.128
SKN2-12	SKN2-14	0.004
SKN2-13	SKN2-14	–0.120
SKN2-1	SKN2-15	–0.036
SKN2-2	SKN2-15	0.019
SKN2-3	SKN2-15	0.043
SKN2-4	SKN2-15	0.000
SKN2-5	SKN2-15	0.024
SKN2-6	SKN2-15	–0.021
SKN2-7	SKN2-15	–0.020
SKN2-8	SKN2-15	0.005
SKN2-9	SKN2-15	–0.066
SKN2-10	SKN2-15	–0.060
SKN2-11	SKN2-15	–0.081
SKN2-12	SKN2-15	–0.069
SKN2-13	SKN2-15	–0.090
SKN2-14	SKN2-15	–0.028
SKN2-1	SKN2-16	–0.094
SKN2-2	SKN2-16	–0.093

Sample 1	Sample 2	*r*
SKN2-3	SKN2-16	0.037
SKN2-4	SKN2-16	0.005
SKN2-5	SKN2-16	0.065
SKN2-6	SKN2-16	0.021
SKN2-7	SKN2-16	–0.037
SKN2-8	SKN2-16	–0.053
SKN2-9	SKN2-16	0.030
SKN2-10	SKN2-16	0.005
SKN2-11	SKN2-16	–0.014
SKN2-12	SKN2-16	–0.041
SKN2-13	SKN2-16	0.011
SKN2-14	SKN2-16	–0.093
SKN2-15	SKN2-16	–0.006
SKN2-1	SKN2-17	–0.069
SKN2-2	SKN2-17	0.007
SKN2-3	SKN2-17	–0.068
SKN2-4	SKN2-17	–0.070
SKN2-5	SKN2-17	–0.044
SKN2-6	SKN2-17	–0.054
SKN2-7	SKN2-17	–0.111
SKN2-8	SKN2-17	–0.083
SKN2-9	SKN2-17	–0.091
SKN2-10	SKN2-17	0.079
SKN2-11	SKN2-17	–0.034
SKN2-12	SKN2-17	–0.001
SKN2-13	SKN2-17	–0.045
SKN2-14	SKN2-17	–0.040
SKN2-15	SKN2-17	–0.036
SKN2-16	SKN2-17	–0.038
SKN2-1	SKN2-18	–0.063
SKN2-2	SKN2-18	–0.106
SKN2-3	SKN2-18	–0.056
SKN2-4	SKN2-18	0.015
SKN2-5	SKN2-18	–0.069
SKN2-6	SKN2-18	0.042
SKN2-7	SKN2-18	0.037
SKN2-8	SKN2-18	–0.024
SKN2-9	SKN2-18	0.038
SKN2-10	SKN2-18	–0.066
SKN2-11	SKN2-18	–0.071
SKN2-12	SKN2-18	–0.104
SKN2-13	SKN2-18	0.093
SKN2-14	SKN2-18	–0.079
SKN2-15	SKN2-18	–0.059
SKN2-16	SKN2-18	–0.024
SKN2-17	SKN2-18	0.131
SKN2-1	SKN2-19	–0.063
SKN2-2	SKN2-19	0.013
SKN2-3	SKN2-19	0.057
SKN2-4	SKN2-19	–0.081
SKN2-5	SKN2-19	0.117
SKN2-6	SKN2-19	0.053
SKN2-7	SKN2-19	–0.044
SKN2-8	SKN2-19	–0.023
SKN2-9	SKN2-19	–0.062

Sample 1	Sample 2	*r*
SKN2-10	SKN2-19	–0.076
SKN2-11	SKN2-19	–0.105
SKN2-12	SKN2-19	–0.128
SKN2-13	SKN2-19	–0.113
SKN2-14	SKN2-19	0.000
SKN2-15	SKN2-19	0.005
SKN2-16	SKN2-19	–0.055
SKN2-17	SKN2-19	–0.087
SKN2-18	SKN2-19	–0.095
SKN2-1	SKN2-20	–0.126
SKN2-2	SKN2-20	–0.014
SKN2-3	SKN2-20	–0.123
SKN2-4	SKN2-20	0.075
SKN2-5	SKN2-20	0.077
SKN2-6	SKN2-20	0.103
SKN2-7	SKN2-20	–0.086
SKN2-8	SKN2-20	–0.105
SKN2-9	SKN2-20	–0.081
SKN2-10	SKN2-20	–0.033
SKN2-11	SKN2-20	–0.056
SKN2-12	SKN2-20	0.062
SKN2-13	SKN2-20	–0.059
SKN2-14	SKN2-20	–0.050
SKN2-15	SKN2-20	–0.095
SKN2-16	SKN2-20	–0.022
SKN2-17	SKN2-20	0.023
SKN2-18	SKN2-20	–0.106
SKN2-19	SKN2-20	0.161

Table S22. Pairwise genetic relatedness (*r*) for all 5 Mahachai betta (*Betta mahachaiensis*) individuals in SKN3.

Sample 1	Sample 2	*r*
SKN3-1	SKN3-2	–0.002
SKN3-1	SKN3-3	0.010
SKN3-2	SKN3-3	–0.082
SKN3-1	SKN3-4	0.001
SKN3-2	SKN3-4	–0.170
SKN3-3	SKN3-4	–0.014
SKN3-1	SKN3-5	–0.154
SKN3-2	SKN3-5	–0.183
SKN3-3	SKN3-5	–0.216
SKN3-4	SKN3-5	–0.211

Table S23. Pairwise genetic relatedness (*r*) for all 10 Mahachai betta (*Betta mahachaiensis*) individuals in SKN4.

Sample 1	Sample 2	*r*
SKN4-1	SKN4-2	–0.002
SKN4-1	SKN4-3	0.010
SKN4-2	SKN4-3	–0.082
SKN4-1	SKN4-4	0.001
SKN4-2	SKN4-4	–0.170
SKN4-3	SKN4-4	–0.014
SKN4-1	SKN4-5	–0.154
SKN4-2	SKN4-5	–0.183
SKN4-3	SKN4-5	–0.216
SKN4-4	SKN4-5	–0.211
SKN4-1	SKN4-6	–0.234
SKN4-2	SKN4-6	0.004
SKN4-3	SKN4-6	0.099
SKN4-4	SKN4-6	–0.101
SKN4-5	SKN4-6	0.040
SKN4-1	SKN4-7	–0.151
SKN4-2	SKN4-7	0.000
SKN4-3	SKN4-7	–0.099
SKN4-4	SKN4-7	–0.069
SKN4-5	SKN4-7	–0.111
SKN4-6	SKN4-7	–0.052
SKN4-1	SKN4-8	–0.045
SKN4-2	SKN4-8	–0.089
SKN4-3	SKN4-8	–0.014
SKN4-4	SKN4-8	0.011
SKN4-5	SKN4-8	–0.061
SKN4-6	SKN4-8	–0.006
SKN4-7	SKN4-8	0.030
SKN4-1	SKN4-9	0.210
SKN4-2	SKN4-9	–0.056
SKN4-3	SKN4-9	–0.052
SKN4-4	SKN4-9	0.034
SKN4-5	SKN4-9	–0.132
SKN4-6	SKN4-9	–0.197
SKN4-7	SKN4-9	–0.109
SKN4-8	SKN4-9	–0.053
SKN4-1	SKN4-10	–0.170
SKN4-2	SKN4-10	–0.064
SKN4-3	SKN4-10	–0.051
SKN4-4	SKN4-10	–0.129
SKN4-5	SKN4-10	–0.245
SKN4-6	SKN4-10	–0.094
SKN4-7	SKN4-10	0.026
SKN4-8	SKN4-10	–0.052
SKN4-9	SKN4-10	–0.130

Table S24. Pairwise genetic relatedness (*r*) for all 3 Mahachai betta (*Betta mahachaiensis*) individuals in SKN5.

Sample 1	Sample 2	*r*
SKN5-1	SKN5-2	–0.209
SKN5-1	SKN5-3	–0.241
SKN5-2	SKN5-3	–0.248

Table S25. Pairwise genetic relatedness (*r*) for all 3 Mahachai betta (*Betta mahachaiensis*) individuals in SKN6.

Sample 1	Sample 2	*r*
SKN6-1	SKN6-2	–0.241
SKN6-1	SKN6-3	–0.243
SKN6-2	SKN6-3	–0.131

Table S26. Pairwise genetic relatedness (*r*) for all 5 Mahachai betta (*Betta mahachaiensis*) individuals in SKN7.

Sample 1	Sample 2	*r*
SKN7-1	SKN7-2	–0.020
SKN7-1	SKN7-3	–0.240
SKN7-2	SKN7-3	–0.170
SKN7-1	SKN7-4	–0.159
SKN7-2	SKN7-4	0.043
SKN7-3	SKN7-4	–0.234
SKN7-1	SKN7-5	0.000
SKN7-2	SKN7-5	–0.217
SKN7-3	SKN7-5	–0.111
SKN7-4	SKN7-5	–0.153

Table S27. Distributions of *r* values and F_{IS} values for the Mahachai betta (*Betta mahachaiensis*).

Population 1	Population 2	Relatedness (*r*)		Inbreeding coefficient (F_{IS})	
		Density	*p*-value	Density	*p*-value
All	SPK [1]	0.094	0.307	0.370	<0.05
All	BKK1 [2]	0.141	0.285	0.235	0.566
All	BKK2 [3]	0.879	<0.05	0.901	<0.05
All	SKN1 [4]	0.620	<0.05	0.827	<0.05
All	SKN2 [5]	0.134	<0.05	0.395	<0.05
All	SKN3 [6]	0.389	0.074	0.840	<0.05
All	SKN4 [7]	0.141	0.385	0.246	0.558
All	SKN5 [8]	0.959	<0.05	0.444	0.504
All	SKN6 [9]	0.859	<0.05	0.519	0.321
All	SKN7 [10]	0.496	<0.05	0.578	0.053
BKK1 [2]	BKK1 [2]	0.192	0.089	0.455	0.092
BKK1 [2]	BKK2 [3]	0.897	<0.05	1.000	<0.05
BKK1 [2]	SKN1 [4]	0.640	<0.05	1.000	<0.05
BKK1 [2]	SKN2 [5]	0.112	0.269	0.203	0.745
BKK1 [2]	SKN3 [6]	0.397	0.074	1.000	<0.05
BKK1 [2]	SKN4 [7]	0.225	0.050	0.524	<0.05
BKK1 [2]	SKN5 [8]	0.985	<0.05	0.667	0.119
BKK1 [2]	SKN6 [9]	0.882	<0.05	0.824	<0.05
BKK1 [2]	SKN7 [10]	0.531	<0.05	0.800	<0.05
BKK1	BKK2 [3]	0.818	<0.05	1.000	<0.05
BKK1	SKN1 [4]	0.509	0.076	1.000	<0.05
BKK1	SKN2 [5]	0.269	<0.05	0.455	0.073
BKK1	SKN3 [6]	0.355	0.192	1.000	<0.05
BKK1	SKN4 [7]	0.119	0.816	0.264	0.737
BKK1	SKN5 [8]	0.982	<0.05	0.455	0.615
BKK1	SKN6 [9]	0.764	<0.05	0.667	0.154
BKK1	SKN7 [10]	0.455	<0.05	0.800	<0.05
BKK2 [3]	SKN1 [4]	0.667	0.333	0.750	0.229
BKK2 [3]	SKN2 [5]	0.995	<0.05	1.000	<0.05
BKK2 [3]	SKN3 [6]	0.500	0.434	0.400	0.857
BKK2 [3]	SKN4 [7]	0.778	<0.05	1.000	<0.05
BKK2 [3]	SKN5 [8]	1.000	0.100	1.000	0.100
BKK2 [3]	SKN6 [9]	0.667	0.600	0.667	0.600
BKK2 [3]	SKN7 [10]	0.400	0.720	1.000	<0.05

Population 1	Population 2	Relatedness (*r*)		Inbreeding coefficient (F_{IS})	
		Density	*p*-value	Density	*p*-value
SKN1 [4]	SKN2 [5]	0.732	<0.05	1.000	<0.05
SKN1 [4]	SKN3 [6]	0.400	0.505	0.400	0.714
SKN1 [4]	SKN4 [7]	0.556	<0.05	1.000	<0.05
SKN1 [4]	SKN5 [8]	0.667	0.333	1.000	0.057
SKN1 [4]	SKN6 [9]	0.667	0.333	0.667	0.343
SKN1 [4]	SKN7 [10]	0.300	0.835	0.800	0.079
SKN2 [5]	SKN3 [6]	0.495	<0.05	1.000	<0.05
SKN2 [5]	SKN4 [7]	0.273	<0.05	0.600	<0.05
SKN2 [5]	SKN5 [8]	1.000	<0.05	0.800	<0.05
SKN2 [5]	SKN6 [9]	0.984	<0.05	0.900	<0.05
SKN2 [5]	SKN7 [10]	0.626	<0.05	0.800	<0.05
SKN3 [6]	SKN4 [7]	0.300	0.356	1.000	<0.05
SKN3 [6]	SKN5 [8]	0.800	0.070	1.000	<0.05
SKN3 [6]	SKN6 [9]	0.667	0.189	0.667	0.286
SKN3 [6]	SKN7 [10]	0.300	0.763	0.800	0.079
SKN4 [7]	SKN5 [8]	0.911	<0.05	0.367	0.832
SKN4 [7]	SKN6 [9]	0.756	<0.05	0.567	0.371
SKN4 [7]	SKN7 [10]	0.400	0.111	0.700	0.061
SKN5 [8]	SKN6 [9]	0.333	1.000	0.667	0.600
SKN5 [8]	SKN7 [10]	0.700	0.140	0.800	0.143
SKN6 [9]	SKN6 [9]	0.567	0.371	0.467	0.679

[1] SPK = Samut Prakan. [2] BKK = Bangkok 1. [3] BKK2 = Bangkok 2. [4] SKN1 = Samut Sakhon 1. [5] SKN2 = Samut Sakhon 2. [6] SKN3 = Samut Sakhon 3. [7] SKN4 = Samut Sakhon 4. [8] SKN5 = Samut Sakhon 5. [9] SKN6 = Samut Sakhon 6. [10] SKN7 = Samut Sakhon 7.

Table S28. Pairwise inbreeding coefficients (F_{IS}) for all 17 Mahachai betta (*Betta mahachaiensis*) individuals in SPK.

Individual	F_{IS}
SPK-1	–0.181
SPK-2	–0.168
SPK-3	–0.229
SPK-4	–0.084
SPK-5	–0.222
SPK-6	–0.137
SPK-7	–0.122
SPK-8	–0.154
SPK-9	–0.184
SPK-10	–0.123
SPK-11	–0.218
SPK-12	–0.160
SPK-13	–0.080
SPK-14	–0.096
SPK-15	–0.168
SPK-16	–0.191
SPK-17	–0.147

Table S29. Pairwise inbreeding coefficients (F_{IS}) for all 11 Mahachai betta (*Betta mahachaiensis*) individuals in BKK1.

Individual	F_{IS}
BKK1-1	–0.105
BKK1-2	–0.268
BKK1-3	–0.162
BKK1-4	–0.230
BKK1-5	–0.319
BKK1-6	–0.037
BKK1-7	–0.150
BKK1-8	–0.195
BKK1-9	–0.315
BKK1-10	–0.325
BKK1-11	–0.171

Table S30. Pairwise inbreeding coefficients (F_{IS}) for all 3 Mahachai betta (*Betta mahachaiensis*) individuals in BKK2.

Individual	F_{IS}
BKK2-1	–0.600
BKK2-2	–1.000
BKK2-3	–0.667

Table S31. Pairwise inbreeding coefficients (F_{IS}) for all 4 Mahachai betta (*Betta mahachaiensis*) individuals in SKN1.

Individual	F_{IS}
SKN1-1	–0.571
SKN1-2	–0.533
SKN1-3	–0.667
SKN1-4	–0.571

Table S32. Pairwise inbreeding coefficients (F_{IS}) for all 20 Mahachai betta (*Betta mahachaiensis*) individuals in SKN2.

Individual	F_{IS}
SKN2-1	–0.160
SKN2-2	–0.144
SKN2-3	–0.082
SKN2-4	–0.175
SKN2-5	–0.047
SKN2-6	–0.126
SKN2-7	–0.176
SKN2-8	–0.180
SKN2-9	–0.189
SKN2-10	–0.095
SKN2-11	–0.142
SKN2-12	–0.123
SKN2-13	–0.150
SKN2-14	–0.220
SKN2-15	0.006
SKN2-16	–0.109
SKN2-17	–0.134
SKN2-18	–0.179
SKN2-19	–0.206
SKN2-20	–0.045

Table S33. Pairwise inbreeding coefficients (F_{IS}) for all 5 Mahachai betta (*Betta mahachaiensis*) individuals in SKN3.

Individual	F_{IS}
SKN3-1	–0.557
SKN3-2	–0.548
SKN3-3	–0.671
SKN3-4	–0.857
SKN3-5	–0.619

Table S34. Pairwise inbreeding coefficients (F_{IS}) for all 10 Mahachai betta (*Betta mahachaiensis*) individuals in SKN4.

Individual	F_{IS}
SKN4-1	–0.058
SKN4-2	–0.174
SKN4-3	–0.301
SKN4-4	–0.163
SKN4-5	–0.205
SKN4-6	–0.474
SKN4-7	–0.289
SKN4-8	–0.234
SKN4-9	–0.265
SKN4-10	–0.197

Table S35. Pairwise inbreeding coefficients (F_{IS}) for all 3 Mahachai betta (*Betta mahachaiensis*) individuals in SKN5.

Individual	F_{IS}
SKN5-1	–0.180
SKN5-2	–0.311
SKN5-3	–0.278

Table S36. Pairwise inbreeding coefficients (F_{IS}) for all 3 Mahachai betta (*Betta mahachaiensis*) individuals in SKN6.

Individual	F_{IS}
SKN6-1	–0.625
SKN6-2	–0.192
SKN6-3	–0.356

Table S37. Pairwise inbreeding coefficients (F_{IS}) for all 5 Mahachai betta (*Betta mahachaiensis*) individuals in SKN7.

Individual	F_{IS}
SKN7-1	–0.114
SKN7-2	–0.432
SKN7-3	–0.373
SKN7-4	–0.408
SKN7-5	–0.556

Table S38. Pairwise genetic differentiation (F_{ST}), pairwise F_{ST}^{ENA} values with ENA correction for null alleles and R_{ST} values using FSTAT version 2.9.3 (Goudet, 1995) of Mahachai betta (*Betta mahachaiensis*) based on 13 microsatellite loci. The number indicates *p* values, with 110 permutations.

Combination	F_{ST}	F_{ST}^{ENA}	R_{ST}
SPK [1] x BKK1 [2]	0.194^{ns}	0.162	0.139
SPK [1] x BKK2 [3]	0.246^{ns}	0.267	0.170
SPK [1] x SKN1 [4]	0.293^{ns}	0.266	0.249
SPK [1] x SKN2 [5]	0.184^{ns}	0.163	0.127
SPK [1] x SKN3 [6]	0.254^{ns}	0.252	0.226
SPK [1] x SKN4 [7]	0.215^{ns}	0.184	0.123
SPK [1] x SKN5 [8]	0.231^{ns}	0.213	0.273
SPK [1] x SKN6 [9]	0.228^{ns}	0.229	0.252
SPK [1] x SKN7 [10]	0.272^{ns}	0.255	0.220
BKK1 [2] x BKK2 [3]	0.194^{ns}	0.308	0.030
BKK1 [2] x SKN1 [4]	0.246*	0.099	–0.024
BKK1 [2] x SKN2 [5]	0.293*	0.075	–0.019
BKK1 [2] x SKN3 [6]	0.184*	0.202	0.113
BKK1 [2] x SKN4 [7]	0.254^{ns}	0.116	0.147
BKK1 [2] x SKN5 [8]	0.215*	0.226	0.167
BKK1 [2] x SKN6 [9]	0.231^{ns}	0.227	0.237
BKK1 [2] x SKN7 [10]	0.228^{ns}	0.185	0.253
BKK2 [3] x SKN1 [4]	0.400*	0.390	0.360
BKK2 [3] x SKN2 [5]	0.204^{ns}	0.229	0.239
BKK2 [3] x SKN3 [6]	0.378*	0.371	0.153
BKK2 [3] x SKN4 [7]	0.217^{ns}	0.228	0.103
BKK2 [3] x SKN5 [8]	0.343^{ns}	0.379	0.333
BKK2 [3] x SKN6 [9]	0.217^{ns}	0.245	0.263
BKK2 [3] x SKN7 [10]	0.386^{ns}	0.386	0.216
SKN1 [4] x SKN2	0.045*	0.050	0.169
SKN1 [4] x SKN3 [6]	0.221*	0.184	0.111
SKN1 [4] x SKN4 [7]	0.106*	0.104	0.229
SKN1 [4] x SKN5 [8]	0.220^{ns}	0.237	0.238
SKN1 [4] x SKN6 [9]	0.285^{ns}	0.287	0.243
SKN1 [4] x SKN7 [10]	0.229^{ns}	0.188	0.358
SKN2 [5] x SKN3 [6]	0.081*	0.099	0.265
SKN2 [5] x SKN4 [7]	0.034*	0.032	0.086
SKN2 [5] x SKN5 [8]	0.086*	0.095	0.108
SKN2 [5] x SKN6 [9]	0.138*	0.155	0.315
SKN2 [5] x SKN7 [10]	0.112^{ns}	0.122	0.160
SKN3 [6] x SKN4 [7]	0.196*	0.184	0.183
SKN3 [6] x SKN5 [8]	0.251^{ns}	0.281	0.219
SKN3 [6] x SKN6 [9]	0.286^{ns}	0.307	0.204
SKN3 [6] x SKN7 [10]	0.230^{ns}	0.208	0.253
SKN4 [7] x SKN5 [8]	0.043*	0.037	–0.006
SKN4 [7] x SKN6 [9]	0.080*	0.085	0.276

Combination	F_{ST}	F_{ST}^{ENA}	R_{ST}
SKN4 [7] x SKN7 [10]	0.186*	0.165	0.098
SKN5 [8] x SKN6 [9]	0.027*	0.092	0.150
SKN5 [8] x SKN7 [10]	0.189*	0.182	0.015
SKN6 [9] x SKN7 [10]	0.252*	0.264	0.394

[1] SPK = Samut Prakan. [2] BKK = Bangkok 1. [3] BKK2 = Bangkok 2. [4] SKN1 = Samut Sakhon 1. [5] SKN2 = Samut Sakhon 2. [6] SKN3 = Samut Sakhon 3. [7] SKN4 = Samut Sakhon 4. [8] SKN5 = Samut Sakhon 5. [9] SKN6 = Samut Sakhon 6. [10] SKN7 = Samut Sakhon 7. * = statistically significant difference. ns = not statistically significant.

Goudet, J.F. 1995. FSTAT (version 1.2): A computer program to calculate F-statistics. J. Hered 86, 485– 486. https://doi.org/10.1093/oxfordjournals.jhered.a111627.

Table S39. Analysis of molecular variance (AMOVA) results for Mahachai betta (*Betta mahachaiensis*) individuals based on 13 microsatellite loci using Arlequin version 3.5.2.2 (Excoffier & Lischer, 2010).

Source of variation	df	Sum of squares	Variance components	Percentage of variation
Among populations	9	107.498	0.553	16%
Among individual	71	249.273	0.660	19%
Within individual	81	177.500	2.191	64%
Total	161	534.272	3.404	100%

Excoffier, L., Lischer, H.E. 2010. Arlequin suite ver 3.5: A new series of programs to perform population genetics analyses under Linux and Windows. Mol. Ecol. Resour. 10, 564–567. https:// doi.org/10.1111/j.1755-0998.2010.02847.x.

Table S40. Pairwise population Nei's genetic distance (*D*) values using GenAlEx version 6.5 (Peakall & Smouse, 2012) of Mahachai betta (*Betta mahachaiensis*) individuals based on 13 microsatellite loci.

Nei D	SPK	BKK1	BKK2	SKN1	SKN2	SKN3	SKN4	SKN5	SKN6	SKN7
SPK [1]	0.000									
BKK1 [2]	0.240	0.000								
BKK2 [3]	0.325	0.406	0.000							
SKN1 [4]	0.426	0.179	0.375	0.000						
SKN2 [5]	0.218	0.133	0.377	0.170	0.000					
SKN3 [6]	0.308	0.264	0.432	0.242	0.121	0.000				
SKN4 [7]	0.330	0.257	0.499	0.293	0.091	0.297	0.000			
SKN5 [8]	0.581	0.683	0.771	0.675	0.451	0.728	0.371	0.000		
SKN6 [9]	0.494	0.476	0.395	0.562	0.375	0.478	0.316	0.521	0.000	
SKN7 [10]	0.297	0.229	0.421	0.278	0.182	0.269	0.319	0.477	0.473	0.000

[1] SPK = Samut Prakan. [2] BKK = Bangkok 1. [3] BKK2 = Bangkok 2. [4] SKN1 = Samut Sakhon 1. [5] SKN2 = Samut Sakhon 2. [6] SKN3 = Samut Sakhon 3. [7] SKN4 = Samut Sakhon 4. [8] SKN5 = Samut Sakhon 5. [9] SKN6 = Samut Sakhon 6. [10] SKN7 = Samut Sakhon 7.

Peakall, R., Smouse, P.E. 2012. GenAlEx 6.5: genetic analysis in Excel. Population genetic software for teaching and research–an update. Bioinformatics 28, 2537–2539. https://doi.org/ 10.1093/bioinformatics/bts4

Table S41. The Wilcoxon signed-rank test to assess mutation drift equilibrium across various models in 81 sample of Mahachai betta (*Betta mahachaiensis*).

Locality	Wilcoxon test		Mode-shift test
	T.P.M.	S.M.M	
	Probability for one tail test for *H* excess	Probability for one tail test for *H* excess	
SPK [1]	0.945	0.954	normal L-shaped distribution
BKK1 [2]	0.945	0.945	normal L-shaped distribution
BKK2 [3]	0.922	0.953	Shifted mode
SKN1 [4]	0.004	0.004	Shifted mode
SKN2 [5]	0.974	0.979	normal L-shaped distribution
SKN3 [6]	0.711	0.711	Shifted mode
SKN4 [7]	0.741	0.765	normal L-shaped distribution
SKN5 [8]	0.633	0.633	Shifted mode
SKN6 [9]	0.618	0.711	Shifted mode
SKN7 [10]	0.531	0.531	Shifted mode

[1] SPK = Samut Prakan. [2] BKK = Bangkok 1. [3] BKK2 = Bangkok 2. [4] SKN1 = Samut Sakhon 1. [5] SKN2 = Samut Sakhon 2. [6] SKN3 = Samut Sakhon 3. [7] SKN4 = Samut Sakhon 4. [8] SKN5 = Samut Sakhon 5. [9] SKN6 = Samut Sakhon 6. [10] SKN7 = Samut Sakhon 7.

Table S42. Each comparison between the source and recipient populations includes the mean migration rates and their corresponding 95% confidence intervals, which were calculated using microsatellite data and analyzed with BAYESASS.

Migration route	Posterior mean of migration rates	Standard deviation
BKK1 [2] → SPK [1]	0.013	0.012
BKK2 [3] → SPK [1]	0.013	0.012
SKN1 [4] → SPK [1]	0.013	0.012
SKN2 [5] → SPK [1]	0.014	0.014
SKN3 [6] → SPK [1]	0.011	0.011
SKN4 [7] → SPK [1]	0.013	0.012
SKN5 [8] → SPK [1]	0.012	0.012
SKN6 [9] → SPK [1]	0.012	0.012
SKN7 [10] → SPK [1]	0.012	0.012
SPK [1] → SPK [1]	0.888	0.029
BKK1 [2] → BKK1 [2]	0.787	0.042
BKK2 [3] → BKK1 [2]	0.015	0.015
SKN1 [4] → BKK1 [2]	0.016	0.016
SKN2 [5] → BKK1 [2]	0.046	0.030
SKN3 [6] → BKK1 [2]	0.016	0.014
SKN4 [7] → BKK1 [2]	0.017	0.017
SKN5 [8] → BKK1 [2]	0.017	0.016
SKN6 [9] → BKK1 [2]	0.015	0.014
SKN7 [10] → BKK1 [2]	0.016	0.015
SPK [1] → BKK1 [2]	0.056	0.037
BKK1 [2] → BKK2 [3]	0.025	0.024
BKK2 [3] → BKK2 [3]	0.692	0.024
SKN1 [4] → BKK2 [3]	0.026	0.024
SKN2 [5] → BKK2 [3]	0.101	0.042
SKN3 [6] → BKK2 [3]	0.025	0.023
SKN4 [7] → BKK2 [3]	0.028	0.025
SKN5 [8] → BKK2 [3]	0.026	0.024
SKN6 [9] → BKK2 [3]	0.026	0.026
SKN7 [10] → BKK2 [3]	0.025	0.024
SPK [1] → BKK2 [3]	0.025	0.023
BKK1 [2] → SKN1 [4]	0.027	0.026
BKK2 [3] → SKN1 [4]	0.024	0.023
SKN1 [4] → SKN1 [4]	0.691	0.022
SKN2 [5] → SKN1 [4]	0.114	0.043
SKN3 [6] → SKN1 [4]	0.024	0.022
SKN4 [7] → SKN1 [4]	0.023	0.022
SKN5 [8] → SKN1 [4]	0.025	0.024
SKN6 [9] → SKN1 [4]	0.025	0.024
SKN7 [10] → SKN1 [4]	0.025	0.023
SPK [1] → SKN1 [4]	0.024	0.023
BKK1 [2] → SKN2 [5]	0.013	0.013
BKK2 [3] → SKN2 [5]	0.012	0.012
SKN1 [4] → SKN2 [5]	0.012	0.011
SKN2 [5] → SKN2 [5]	0.890	0.030
SKN3 [6] → SKN2 [5]	0.012	0.013
SKN4 [7] → SKN2 [5]	0.015	0.014
SKN5 [8] → SKN2 [5]	0.011	0.010
SKN6 [9] → SKN2 [5]	0.013	0.012
SKN7 [10] → SKN2 [5]	0.011	0.010

Migration route	Posterior mean of migration rates	Standard deviation
SPK [1] → SKN2 [5]	0.011	0.010
BKK1 [2] → SKN3 [6]	0.022	0.020
BKK2 [3] → SKN3 [6]	0.023	0.022
SKN1 [4] → SKN3 [6]	0.022	0.021
SKN2 [5] → SKN3 [6]	0.134	0.043
SKN3 [6] → SKN3 [6]	0.689	0.021
SKN4 [7] → SKN3 [6]	0.023	0.022
SKN5 [8] → SKN3 [6]	0.021	0.021
SKN6 [9] → SKN3 [6]	0.021	0.019
SKN7 [10] → SKN3 [6]	0.023	0.022
SPK [1] → SKN3 [6]	0.023	0.021
BKK1 [2] → SKN4 [7]	0.017	0.016
BKK2 [3] → SKN4 [7]	0.016	0.015
SKN1 [4] → SKN4 [7]	0.016	0.015
SKN2 [5] → SKN4 [7]	0.118	0.069
SKN3 [6] → SKN4 [7]	0.016	0.014
SKN4 [7] → SKN4 [7]	0.749	0.064
SKN5 [8] → SKN4 [7]	0.017	0.016
SKN6 [9] → SKN4 [7]	0.016	0.016
SKN7 [10] → SKN4 [7]	0.017	0.017
SPK [1] → SKN4 [7]	0.018	0.017
BKK1 [2] → SKN5 [8]	0.027	0.024
BKK2 [3] → SKN5 [8]	0.026	0.024
SKN1 [4] → SKN5 [8]	0.025	0.024
SKN2 [5] → SKN5 [8]	0.048	0.034
SKN3 [6] → SKN5 [8]	0.025	0.023
SKN4 [7] → SKN5 [8]	0.080	0.041
SKN5 [8] → SKN5 [8]	0.693	0.024
SKN6 [9] → SKN5 [8]	0.026	0.023
SKN7 [10] → SKN5 [8]	0.026	0.024
SPK [1] → SKN5 [8]	0.025	0.024
BKK1 [2] → SKN6	0.026	0.023
BKK2 [3] → SKN6 [9]	0.027	0.024
SKN1 [4] → SKN6 [9]	0.026	0.024
SKN2 [5] → SKN6 [9]	0.048	0.044
SKN3 [6] → SKN6 [9]	0.025	0.023
SKN4 [7] → SKN6 [9]	0.081	0.050
SKN5 [8] → SKN6 [9]	0.026	0.023
SKN6 [9] → SKN6 [9]	0.693	0.024
SKN7 [10] → SKN6 [9]	0.025	0.023
SPK [1] → SKN6 [9]	0.025	0.024
BKK1 [2] → SKN7 [10]	0.022	0.021
BKK2 [3] → SKN7 [10]	0.022	0.020
SKN1 [4] → SKN7 [10]	0.024	0.022
SKN2 [5] → SKN7 [10]	0.135	0.042
SKN3 [6] → SKN7 [10]	0.022	0.021
SKN4 [7] → SKN7 [10]	0.022	0.020
SKN5 [8] → SKN7 [10]	0.022	0.020
SKN6 [9] → SKN7 [10]	0.023	0.021
SKN7 [10] → SKN7 [10]	0.689	0.021
SPK [1] → SKN7 [10]	0.022	0.020

[1] SPK = Samut Prakan. [2] BKK = Bangkok 1. [3] BKK2 = Bangkok 2. [4] SKN1 = Samut Sakhon 1. [5] SKN2 = Samut Sakhon 2. [6] SKN3 = Samut Sakhon 3. [7] SKN4 = Samut Sakhon 4. [8] SKN5 = Samut Sakhon 5. [9] SKN6 = Samut Sakhon 6. [10] SKN7 = Samut Sakhon 7.

Table S43. Bayesian estimates of mutation-scaled effective population sizes (Θ) and asymmetric migration rates (*M*) among 81 Mahachai betta (*Betta mahachaiensis*) for 13 microsatellite loci.

Parameter	2.50%	25.00%	Mode	75.00%	97.50%
ΘSPK [1]	0.000	0.001	0.002	0.003	0.004
ΘBKK1 [2]	0.000	0.000	0.000	0.002	0.004
ΘBKK2 [3]	0.000	0.000	0.000	0.002	0.003
ΘSKN1 [4]	0.000	0.001	0.002	0.003	0.004
ΘSKN2 [5]	0.000	0.006	0.008	0.009	0.010
ΘSKN3 [6]	0.000	0.000	0.000	0.001	0.003
ΘSKN4 [7]	0.001	0.002	0.002	0.003	0.004
ΘSKN5 [8]	0.095	0.096	0.099	0.100	0.100
ΘSKN6 [9]	0.071	0.073	0.078	0.081	0.097
ΘSKN7 [10]	0.000	0.000	0.000	0.002	0.011
$M_{BKK1 \rightarrow SPK}$	142.667	302.000	325.000	346.667	378.000
$M_{BKK2 \rightarrow SPK}$	0.000	37.333	59.000	80.000	152.667
$M_{SKN1 \rightarrow SPK}$	442.000	611.333	670.333	688.667	744.000
$M_{SKN2 \rightarrow SPK}$	609.333	870.667	893.000	939.333	956.000
$M_{SKN3 \rightarrow SPK}$	24.667	46.000	55.667	66.000	122.000
$M_{SKN4 \rightarrow SPK}$	226.667	333.333	353.000	372.667	427.333
$M_{SKN5 \rightarrow SPK}$	392.000	492.667	515.667	537.333	648.667
$M_{SKN6 \rightarrow SPK}$	0.000	39.333	57.667	74.667	86.667
$M_{SKN7 \rightarrow SPK}$	252.000	264.667	287.000	307.333	318.000
$M_{SPK \rightarrow BKK1}$	40.000	44.000	61.667	78.667	82.667
$M_{BKK2 \rightarrow BKK1}$	864.667	876.667	893.667	912.000	996.000
$M_{SKN1 \rightarrow BKK1}$	98.667	126.000	151.667	186.667	259.333
$M_{SKN2 \rightarrow BKK1}$	186.667	214.667	225.667	253.333	285.333
$M_{SKN3 \rightarrow BKK1}$	418.000	443.333	497.000	520.000	600.000
$M_{SKN4 \rightarrow BKK1}$	202.667	263.333	309.000	329.333	462.000
$M_{SKN5 \rightarrow BKK1}$	716.667	758.667	784.333	809.333	976.000
$M_{SKN6 \rightarrow BKK1}$	248.667	336.000	359.667	398.000	458.000
$M_{SKN7 \rightarrow BKK1}$	423.333	478.000	537.667	558.000	686.000
$M_{SPK \rightarrow BKK2}$	647.333	772.667	856.333	882.000	994.000
$M_{BKK1 \rightarrow BKK2}$	306.667	388.000	401.667	415.333	533.333
$M_{SKN1 \rightarrow BKK2}$	286.667	402.000	419.667	437.333	638.667
$M_{SKN2 \rightarrow BKK2}$	7.333	26.667	41.000	56.000	72.667
$M_{SKN3 \rightarrow BKK2}$	621.333	690.000	715.000	741.333	781.333
$M_{SKN4 \rightarrow BKK2}$	702.667	717.333	737.667	766.667	795.333
$M_{SKN5 \rightarrow BKK2}$	345.333	539.333	565.000	615.333	652.667
$M_{SKN6 \rightarrow BKK2}$	394.000	538.667	557.000	572.000	590.667
$M_{SKN7 \rightarrow BKK2}$	0.000	6.667	30.333	72.667	114.667
$M_{SPK \rightarrow SKN1}$	37.333	58.000	99.000	118.000	130.000
$M_{BKK1 \rightarrow SKN1}$	161.333	232.667	267.667	287.333	371.333
$M_{BKK2 \rightarrow SKN1}$	514.000	556.667	584.333	612.000	686.000
$M_{SKN2 \rightarrow SKN1}$	700.667	707.333	727.000	746.667	754.000

Parameter	2.50%	25.00%	Mode	75.00%	97.50%
$M_{SKN3 \rightarrow SKN1}$	215.333	256.000	283.000	312.000	327.333
$M_{SKN4 \rightarrow SKN1}$	441.333	507.333	541.667	572.667	654.000
$M_{SKN5 \rightarrow SKN1}$	132.667	160.667	184.333	204.000	216.000
$M_{SKN6 \rightarrow SKN1}$	716.667	896.667	915.667	953.333	1000.000
$M_{SKN7 \rightarrow SKN1}$	141.333	157.333	172.333	188.000	328.667
$M_{SPK \rightarrow SKN2}$	9.333	50.667	63.667	72.000	82.667
$M_{BKK1 \rightarrow SKN2}$	507.333	660.000	696.333	724.000	854.667
$M_{BKK2 \rightarrow SKN2}$	3.333	22.667	42.333	61.333	178.667
$M_{SKN1 \rightarrow SKN2}$	345.333	355.333	375.667	396.667	660.667
$M_{SKN3 \rightarrow SKN2}$	68.667	111.333	125.000	138.667	162.667
$M_{SKN4 \rightarrow SKN2}$	120.000	238.667	261.667	282.667	292.000
$M_{SKN5 \rightarrow SKN2}$	0.000	12.667	23.667	34.000	54.667
$M_{SKN6 \rightarrow SKN2}$	349.333	416.667	435.000	452.667	592.000
$M_{SKN7 \rightarrow SKN2}$	0.000	0.000	13.000	28.667	42.667
$M_{SPK \rightarrow SKN3}$	0.000	6.000	27.667	50.667	66.000
$M_{BKK1 \rightarrow SKN3}$	141.333	206.000	219.000	231.333	250.000
$M_{BKK2 \rightarrow SKN3}$	20.000	86.667	115.000	135.333	145.333
$M_{SKN1 \rightarrow SKN3}$	398.000	593.333	638.333	664.000	744.667
$M_{SKN2 \rightarrow SKN3}$	183.333	244.667	259.000	274.667	316.000
$M_{SKN4 \rightarrow SKN3}$	590.000	675.333	717.667	752.000	884.667
$M_{SKN5 \rightarrow SKN3}$	11.333	37.333	52.333	68.667	117.333
$M_{SKN6 \rightarrow SKN3}$	97.333	124.667	139.000	152.667	206.000
$M_{SKN7 \rightarrow SKN3}$	248.000	261.333	277.667	294.000	445.333
$M_{SPK \rightarrow SKN4}$	147.333	162.000	193.000	217.333	280.667
$M_{BKK1 \rightarrow SKN4}$	409.333	420.000	438.333	458.667	776.667
$M_{BKK2 \rightarrow SKN4}$	0.000	0.000	5.667	20.000	40.667
$M_{SKN1 \rightarrow SKN4}$	262.000	320.000	337.000	353.333	436.667
$M_{SKN2 \rightarrow SKN4}$	679.333	698.000	719.000	746.000	822.667
$M_{SKN3 \rightarrow SKN4}$	440.000	582.667	607.667	650.667	756.667
$M_{SKN5 \rightarrow SKN4}$	179.333	198.667	209.000	218.667	247.333
$M_{SKN6 \rightarrow SKN4}$	767.333	952.667	979.000	1000.000	1000.000
$M_{SKN7 \rightarrow SKN4}$	7.333	22.000	47.000	68.000	81.333
$M_{SPK \rightarrow SKN5}$	10.667	27.333	35.667	44.000	58.667
$M_{BKK1 \rightarrow SKN5}$	277.333	298.000	323.000	345.333	514.667
$M_{BKK2 \rightarrow SKN5}$	62.000	73.333	90.333	107.333	220.000
$M_{SKN1 \rightarrow SKN5}$	873.333	944.000	967.667	1000.000	1000.000
$M_{SKN2 \rightarrow SKN5}$	395.333	428.667	452.333	480.000	522.000
$M_{SKN3 \rightarrow SKN5}$	0.000	0.000	17.000	36.000	42.667
$M_{SKN4 \rightarrow SKN5}$	706.667	780.000	800.333	820.667	990.667
$M_{SKN6 \rightarrow SKN5}$	379.333	553.333	606.333	640.000	771.333
$M_{SKN7 \rightarrow SKN5}$	0.000	0.000	7.667	15.333	56.000
$M_{SPK \rightarrow SKN6}$	0.000	13.333	24.333	37.333	57.333
$M_{BKK1 \rightarrow SKN6}$	588.667	681.333	704.333	725.333	735.333

Parameter	2.50%	25.00%	Mode	75.00%	97.50%
$M_{BKK2 \rightarrow SKN6}$	13.333	48.000	60.333	70.667	96.667
$M_{SKN1 \rightarrow SKN6}$	284.667	296.000	318.333	345.333	514.000
$M_{SKN2 \rightarrow SKN6}$	39.333	155.333	197.000	220.667	238.667
$M_{SKN3 \rightarrow SKN6}$	38.667	97.333	128.333	147.333	160.667
$M_{SKN4 \rightarrow SKN6}$	14.000	24.667	42.333	60.667	118.667
$M_{SKN5 \rightarrow SKN6}$	765.333	870.000	890.333	908.667	967.333
$M_{SKN7 \rightarrow SKN6}$	400.667	412.000	432.333	452.000	460.667
$M_{SPK \rightarrow SKN7}$	0.000	13.333	24.333	34.000	56.667
$M_{BKK1 \rightarrow SKN7}$	377.333	507.333	535.667	560.667	660.667
$M_{BKK2 \rightarrow SKN7}$	0.000	22.667	32.333	42.000	63.333
$M_{SKN1 \rightarrow SKN7}$	264.000	343.333	359.667	400.000	501.333
$M_{SKN2 \rightarrow SKN7}$	40.000	103.333	118.333	134.000	182.000
$M_{SKN3 \rightarrow SKN7}$	330.667	391.333	411.667	431.333	576.000
$M_{SKN4 \rightarrow SKN7}$	210.000	264.000	285.667	303.333	505.333
$M_{SKN5 \rightarrow SKN7}$	0.000	9.333	37.000	59.333	70.000
$M_{SKN6 \rightarrow SKN7}$	86.667	138.000	152.333	166.667	278.667

[1] SPK = Samut Prakan. [2] BKK = Bangkok 1. [3] BKK2 = Bangkok 2. [4] SKN1 = Samut Sakhon 1. [5] SKN2 = Samut Sakhon 2. [6] SKN3 = Samut Sakhon 3. [7] SKN4 = Samut Sakhon 4. [8] SKN5 = Samut Sakhon 5. [9] SKN6 = Samut Sakhon 6. [10] SKN7 = Samut Sakhon 7.

Table S44. The effective number of immigrants (N_m) from population i into population j per generation.

i \ j	SPK	BKK1	BKK2	SKN1	SKN2	SKN3	SKN4	SKN5	SKN6	SKN7
SPK [1]		0.006	0.021	0.044	0.120	0.000	0.114	0.879	0.475	0.000
BKK1 [2]	0.144		0.010	0.118	1.318	0.002	0.260	7.964	13.740	0.004
BKK2 [3]	0.026	0.083		0.259	0.080	0.001	0.003	2.227	1.177	0.000
SKN1 [4]	0.297	0.014	0.010		0.711	0.005	0.200	23.860	6.210	0.003
SKN2 [5]	0.395	0.021	0.001	0.322		0.002	0.426	11.153	3.843	0.001
SKN3 [6]	0.025	0.046	0.018	0.125	0.237		0.360	0.419	2.503	0.003
SKN4 [7]	0.156	0.029	0.018	0.240	0.495	0.005		19.734	0.826	0.002
SKN5 [8]	0.228	0.073	0.014	0.082	0.045	0.000	0.124		17.368	0.000
SKN6 [9]	0.026	0.033	0.014	0.405	0.823	0.001	0.580	14.951		0.001
SKN7 [10]	0.127	0.050	0.001	0.076	0.025	0.002	0.028	0.189	8.434	

[1] SPK = Samut Prakan. [2] BKK = Bangkok 1. [3] BKK2 = Bangkok 2. [4] SKN1 = Samut Sakhon 1. [5] SKN2 = Samut Sakhon 2. [6] SKN3 = Samut Sakhon 3. [7] SKN4 = Samut Sakhon 4. [8] SKN5 = Samut Sakhon 5. [9] SKN6 = Samut Sakhon 6. [10] SKN7 = Samut Sakhon 7.

Table S45. Multiple linear regression model for the genetic diversity and habitat suitability of *B. mahachaiensis* with landscape-level variable.

Multiple regression model	*p* - value	AIC	R^2
Allelic Richness= 1120.8745 + 1.628X_1 -0.279X_2 – 3.251X_3+ 5.071X_4 - 1.260X_5 + 0.642X_6- 1.078X_7 -42.547X_8	0.978	*	0.495
Expected Heterozygosity= 94.436-0.201X_3+ 0.312X_4- 0.174X_5+ 0.082X_6-3.446 X_7+ 0.050 X_8	0.722	–12.164	0.549
F_{IS} = 363.193+ 0.249X_1 -0.062X_2 -0.742 X_3+ 1.156X_4 - -0.478X_5 + 0.318X_6- 13.550X_7 + 0.124X_8	0.772	–0.399	0.824

Notes: X_1: pH; X_2: Do; X_3: Condutivity, X_4:Salinity; X_5 :Water Temperature; X_6:Precipitation; X_7: Temperature; X_8: Elevation ; AIC: Akaike information criterion; R^2: sum of squared residuals. *None of the candidate covariates were retained in the final model following AIC stepwise selection.

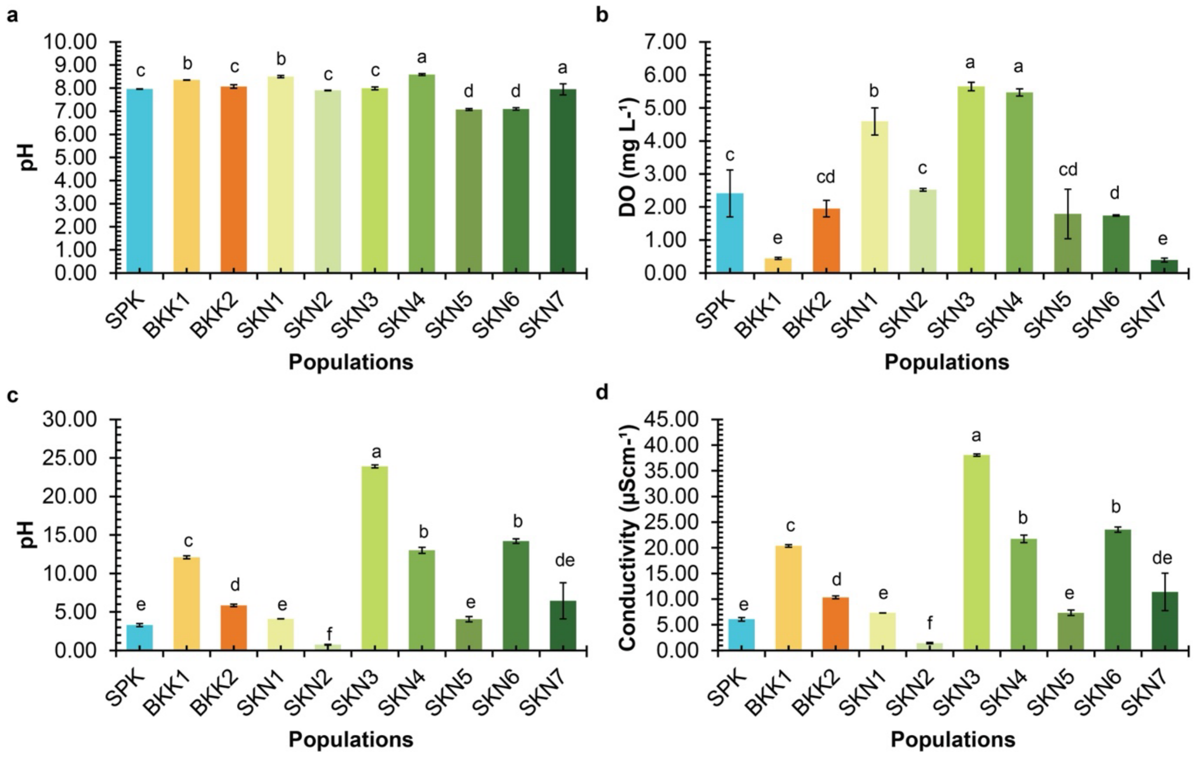

a
pH
10.00
9.00
8.00
7.00
6.00
5.00
4.00
3.00
2.00
1.00
0.00
c b c b c c a d d a
SPK BKK1 BKK2 SKN1 SKN2 SKN3 SKN4 SKN5 SKN6 SKN7
Populations
b
DO (mg L⁻¹)
7.00
6.00
5.00
4.00
3.00
2.00
1.00
0.00
c e cd b c a a cd d e
SPK BKK1 BKK2 SKN1 SKN2 SKN3 SKN4 SKN5 SKN6 SKN7
Populations
c
pH
30.00
25.00
20.00
15.00
10.00
5.00
0.00
e c d e f a b e b de
SPK BKK1 BKK2 SKN1 SKN2 SKN3 SKN4 SKN5 SKN6 SKN7
Populations
d
Conductivity (µScm⁻¹)
45.00
40.00
35.00
30.00
25.00
20.00
15.00
10.00
5.00
0.00
e c d e f a b e b de
SPK BKK1 BKK2 SKN1 SKN2 SKN3 SKN4 SKN5 SKN6 SKN7
Populations

Figure S1.

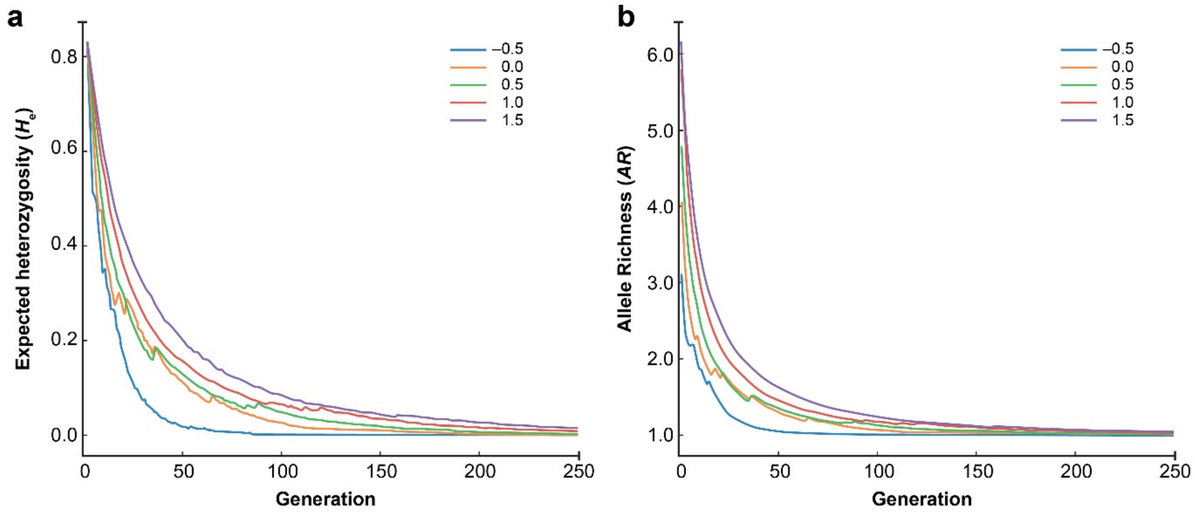

a
Expected heterozygosity (H_e)
0.8
0.6
0.4
0.2
0.0
0
50
100
150
200
250
Generation
–0.5
0.0
0.5
1.0
1.5
b
Allele Richness (*AR*)
6.0
5.0
4.0
3.0
2.0
1.0
0
50
100
150
200
250
Generation
–0.5
0.0
0.5
1.0
1.5

Figure S2.

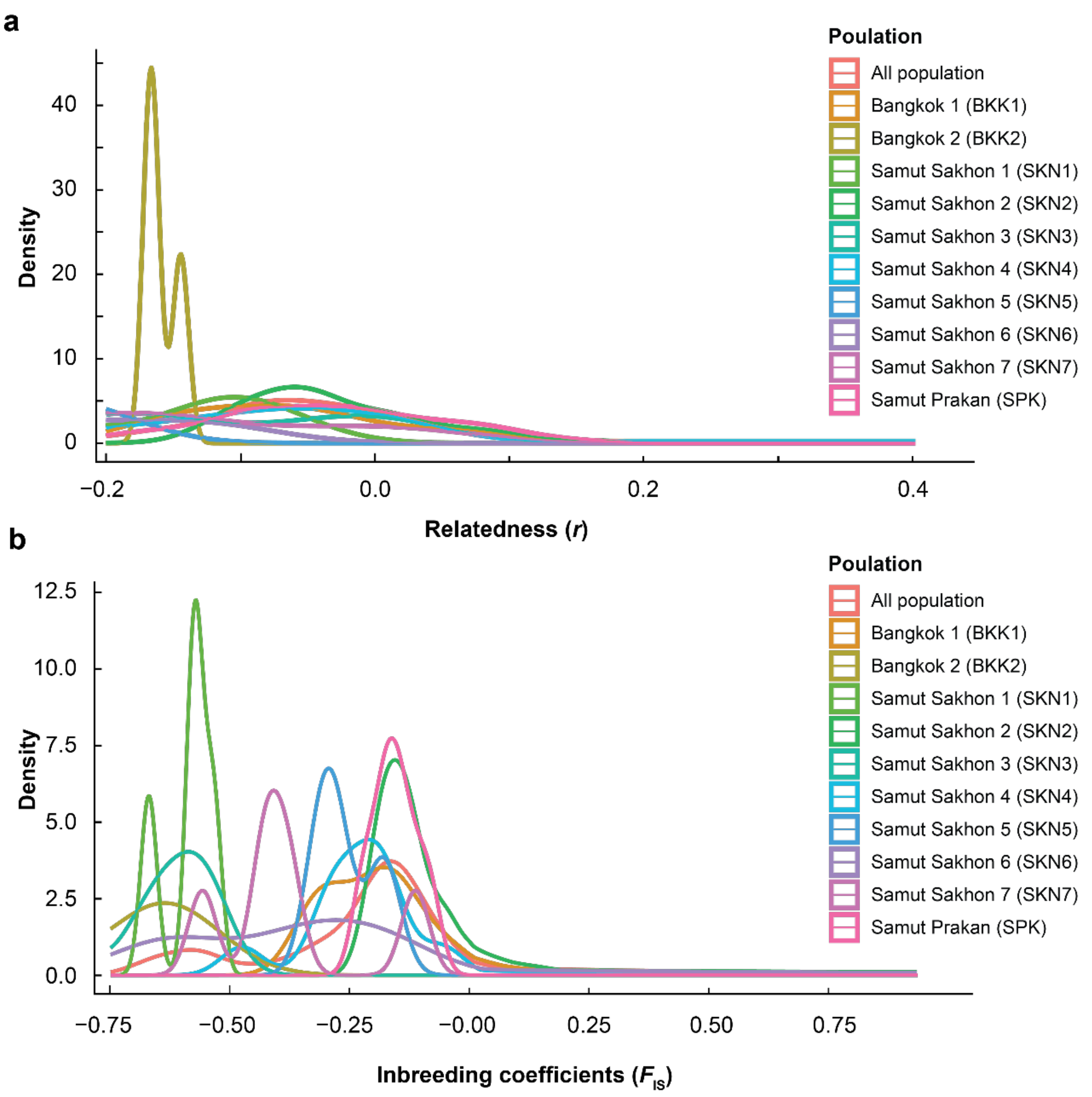
a
Density
40
30
20
10
0
−0.2
0.0
0.2
0.4
Relatedness (r)
Poulation
All population
Bangkok 1 (BKK1)
Bangkok 2 (BKK2)
Samut Sakhon 1 (SKN1)
Samut Sakhon 2 (SKN2)
Samut Sakhon 3 (SKN3)
Samut Sakhon 4 (SKN4)
Samut Sakhon 5 (SKN5)
Samut Sakhon 6 (SKN6)
Samut Sakhon 7 (SKN7)
Samut Prakan (SPK)
b
Density
12.5
10.0
7.5
5.0
2.5
0.0
−0.75
−0.50
−0.25
−0.00
0.25
0.50
0.75
Inbreeding coefficients (F IS)
Poulation
All population
Bangkok 1 (BKK1)
Bangkok 2 (BKK2)
Samut Sakhon 1 (SKN1)
Samut Sakhon 2 (SKN2)
Samut Sakhon 3 (SKN3)
Samut Sakhon 4 (SKN4)
Samut Sakhon 5 (SKN5)
Samut Sakhon 6 (SKN6)
Samut Sakhon 7 (SKN7)
Samut Prakan (SPK)

Figure S3.

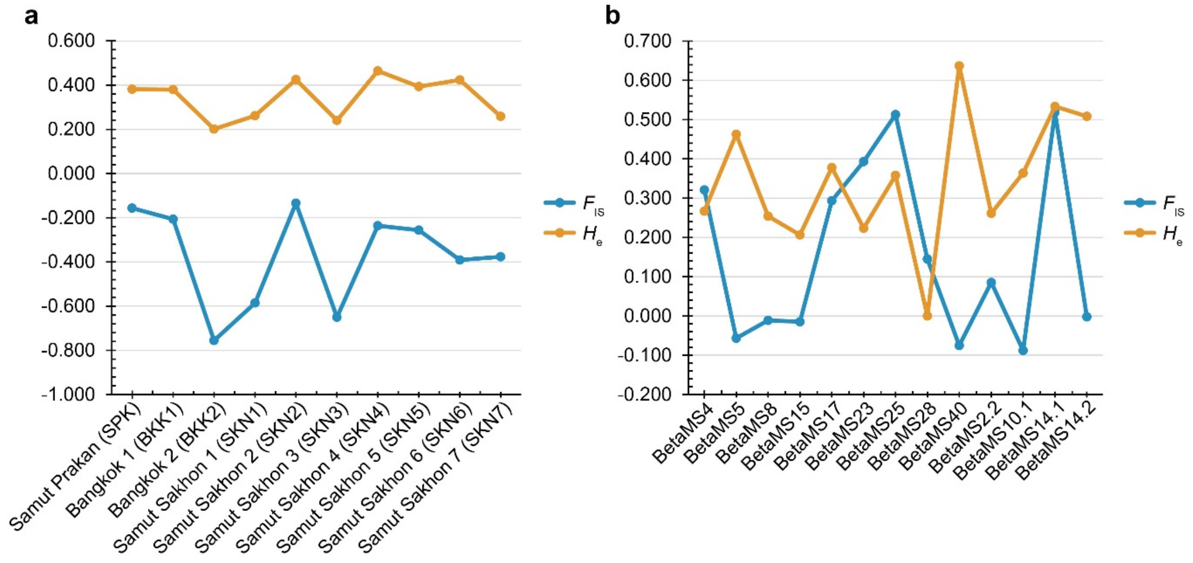

a
0.600
0.400
0.200
0.000
-0.200
-0.400
-0.600
-0.800
-1.000
Samut Prakan (SPK)
Bangkok 1 (BKK1)
Bangkok 2 (BKK2)
Samut Sakhon 1 (SKN1)
Samut Sakhon 2 (SKN2)
Samut Sakhon 3 (SKN3)
Samut Sakhon 4 (SKN4)
Samut Sakhon 5 (SKN5)
Samut Sakhon 6 (SKN6)
Samut Sakhon 7 (SKN7)
F_{IS}
H_e
b
0.700
0.600
0.500
0.400
0.300
0.200
0.100
0.000
-0.100
-0.200
BetaMS4
BetaMS5
BetaMS8
BetaMS15
BetaMS17
BetaMS23
BetaMS25
BetaMS28
BetaMS40
BetaMS2.2
BetaMS10.1
BetaMS14.1
BetaMS14.2
F_{IS}
H_e

Figure S4.

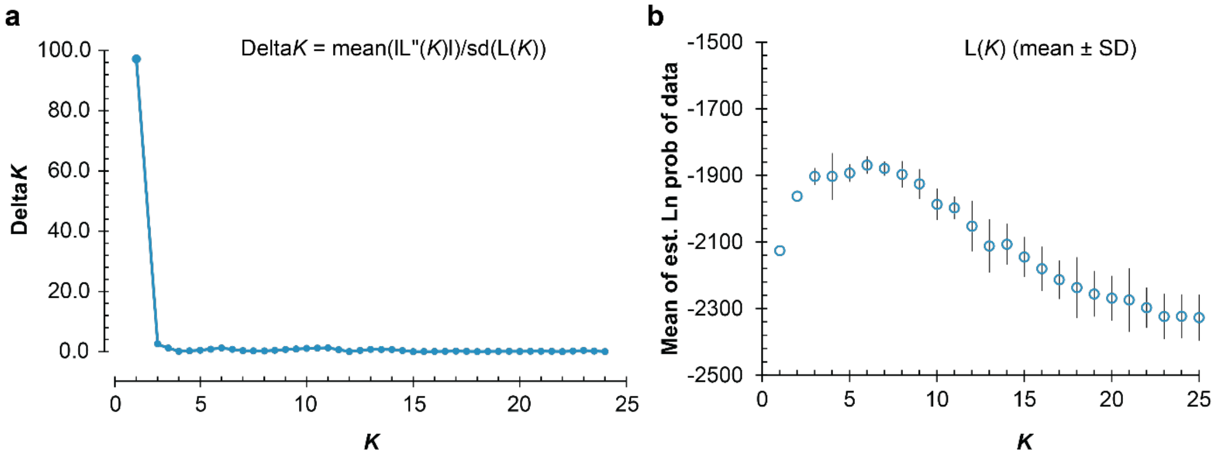
a
b
DeltaK = mean(|L"(K)|)/sd(L(K))
L(K) (mean ± SD)
DeltaK
Mean of est. Ln prob of data
K
K
100.0
80.0
60.0
40.0
20.0
0.0
-1500
-1700
-1900
-2100
-2300
-2500
0
5
10
15
20
25

Figure S5.

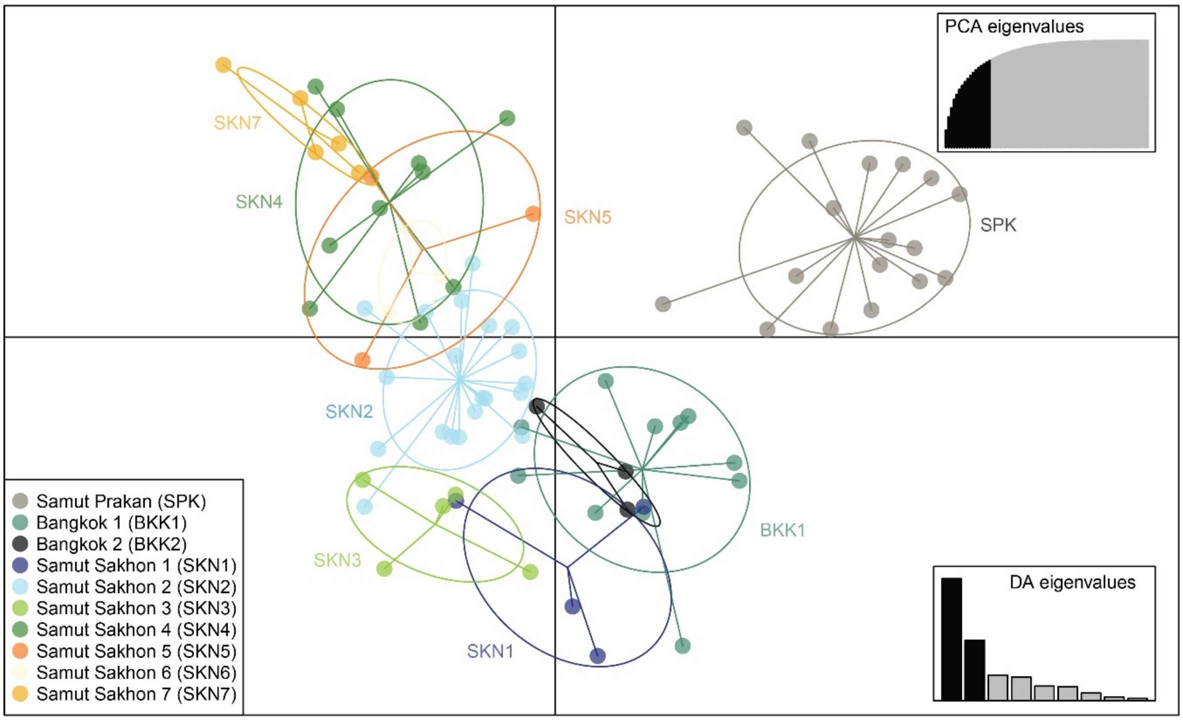
PCA eigenvalues
SKN7
SKN4
SKN5
SPK
SKN2
BKK1
SKN3
SKN1
DA eigenvalues
Samut Prakan (SPK)
Bangkok 1 (BKK1)
Bangkok 2 (BKK2)
Samut Sakhon 1 (SKN1)
Samut Sakhon 2 (SKN2)
Samut Sakhon 3 (SKN3)
Samut Sakhon 4 (SKN4)
Samut Sakhon 5 (SKN5)
Samut Sakhon 6 (SKN6)
Samut Sakhon 7 (SKN7)

Figure S6.

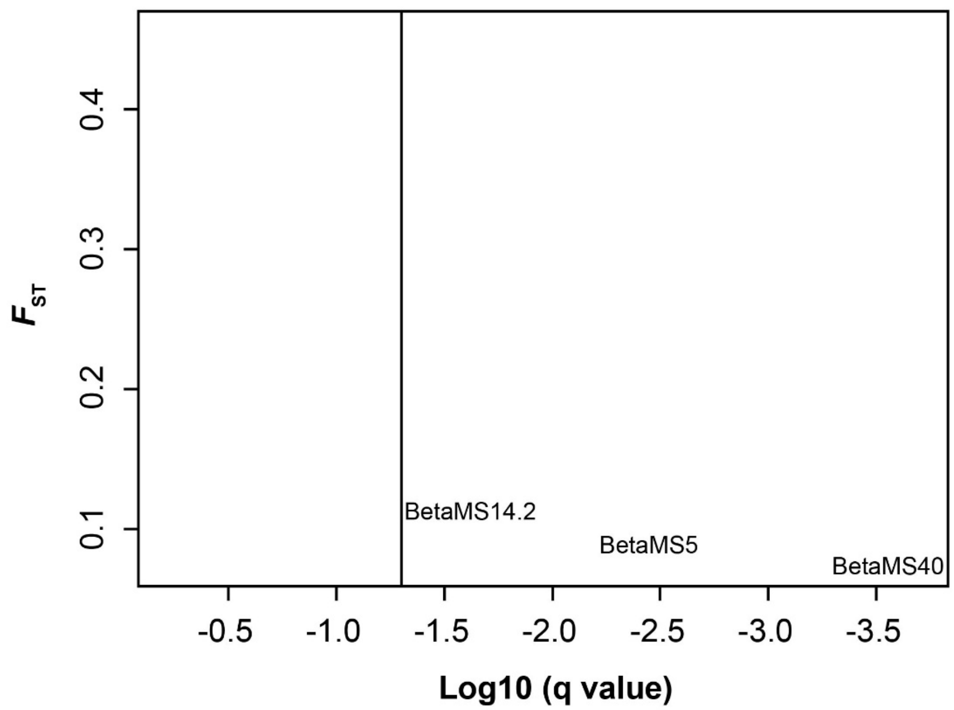

0.4
0.3
0.2
0.1
F_{ST}
BetaMS14.2
BetaMS5
BetaMS40
-0.5
-1.0
-1.5
-2.0
-2.5
-3.0
-3.5
Log10 (q value)

Figure S7.

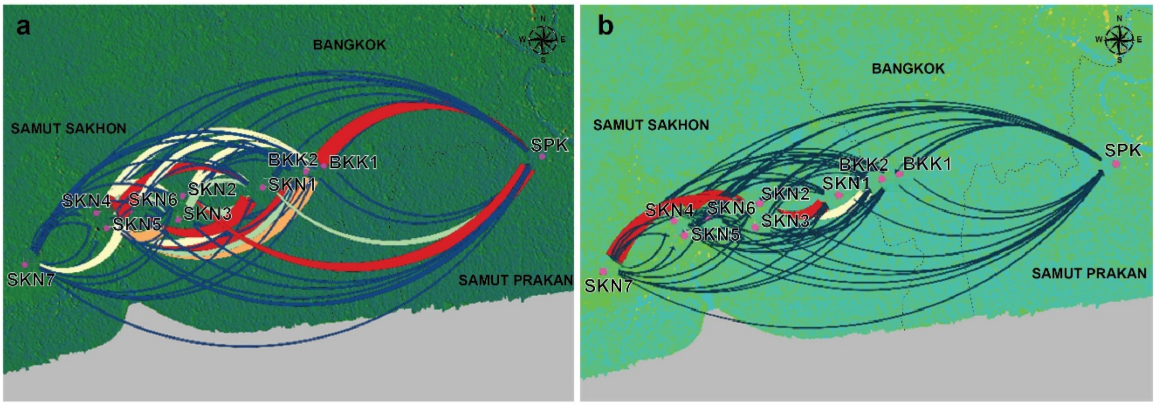

a
BANGKOK
SAMUT SAKHON
SPK
BKK2
BKK1
SKN2
SKN1
SKN4
SKN6
SKN3
SKN5
SKN7
SAMUT PRAKAN
b
BANGKOK
SAMUT SAKHON
SPK
BKK2
BKK1
SKN2
SKN1
SKN4
SKN6
SKN3
SKN5
SKN7
SAMUT PRAKAN

Figure S8.

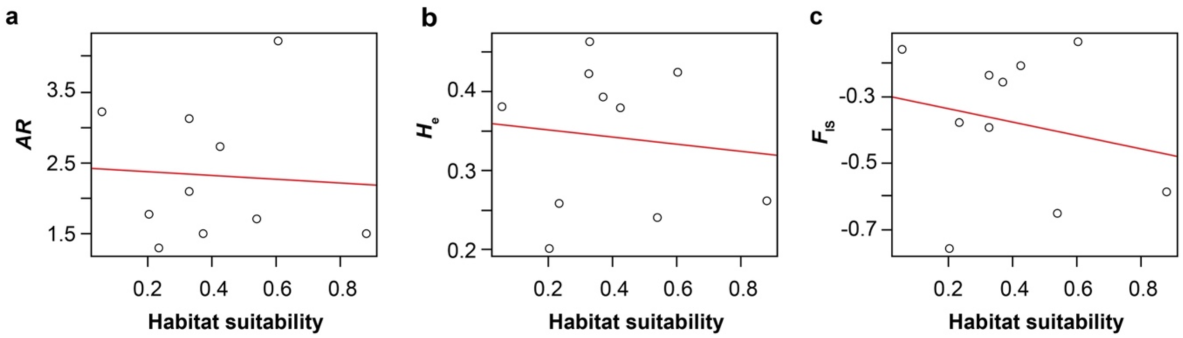
a
AR
3.5
2.5
1.5
0.2
0.4
0.6
0.8
Habitat suitability
b
H_e
0.4
0.3
0.2
0.2
0.4
0.6
0.8
Habitat suitability
c
F_{IS}
-0.3
-0.5
-0.7
0.2
0.4
0.6
0.8
Habitat suitability

Figure S9.

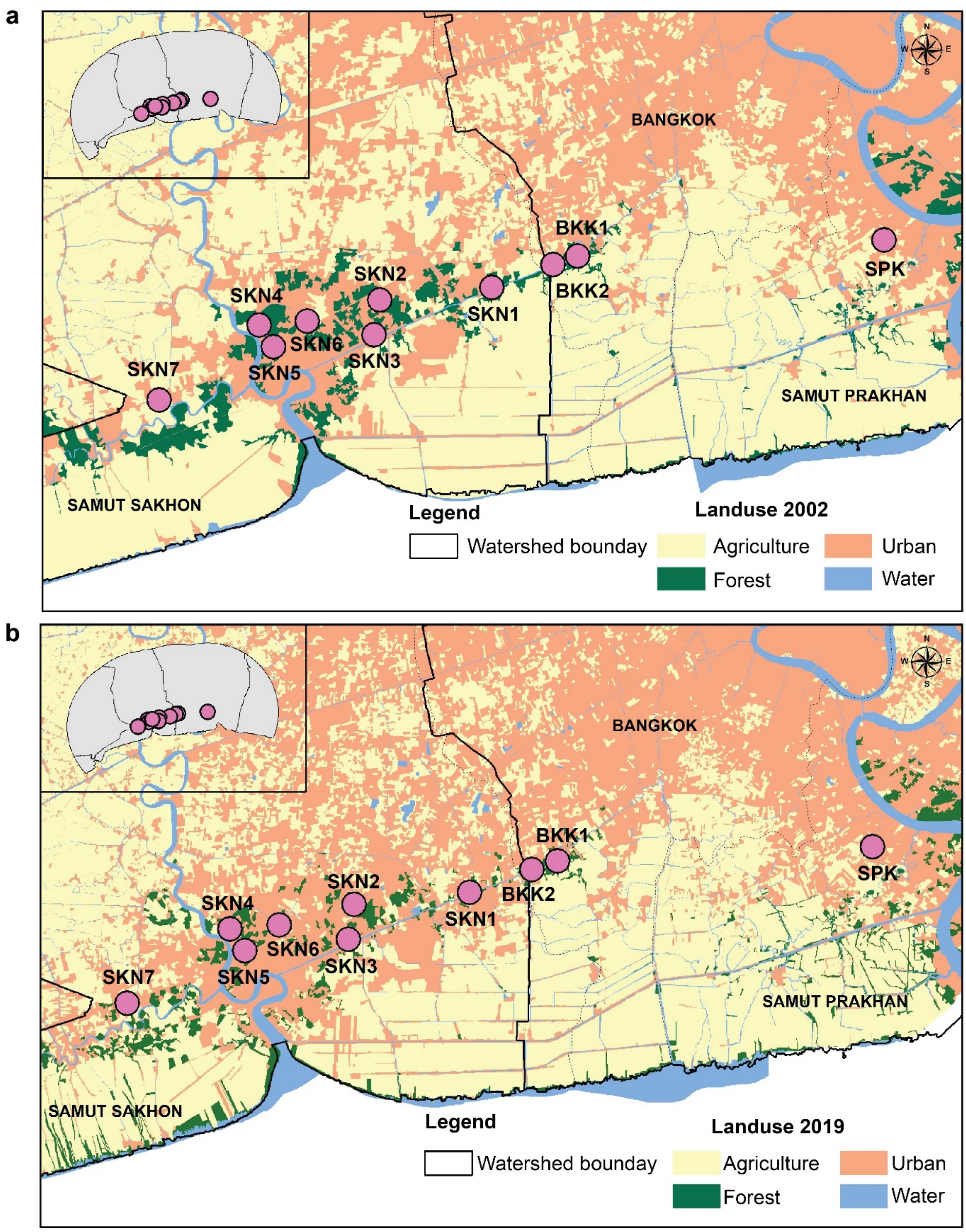
a
BANGKOK
BKK1
BKK2
SPK
SKN1
SKN2
SKN3
SKN4
SKN5
SKN6
SKN7
SAMUT PRAKHAN
SAMUT SAKHON
N
S
W
E
Legend
Landuse 2002
Watershed bounday
Agriculture
Urban
Forest
Water
b
BANGKOK
BKK1
BKK2
SPK
SKN1
SKN2
SKN3
SKN4
SKN5
SKN6
SKN7
SAMUT PRAKHAN
SAMUT SAKHON
N
S
W
E
Legend
Landuse 2019
Watershed bounday
Agriculture
Urban
Forest
Water

Figure S10.

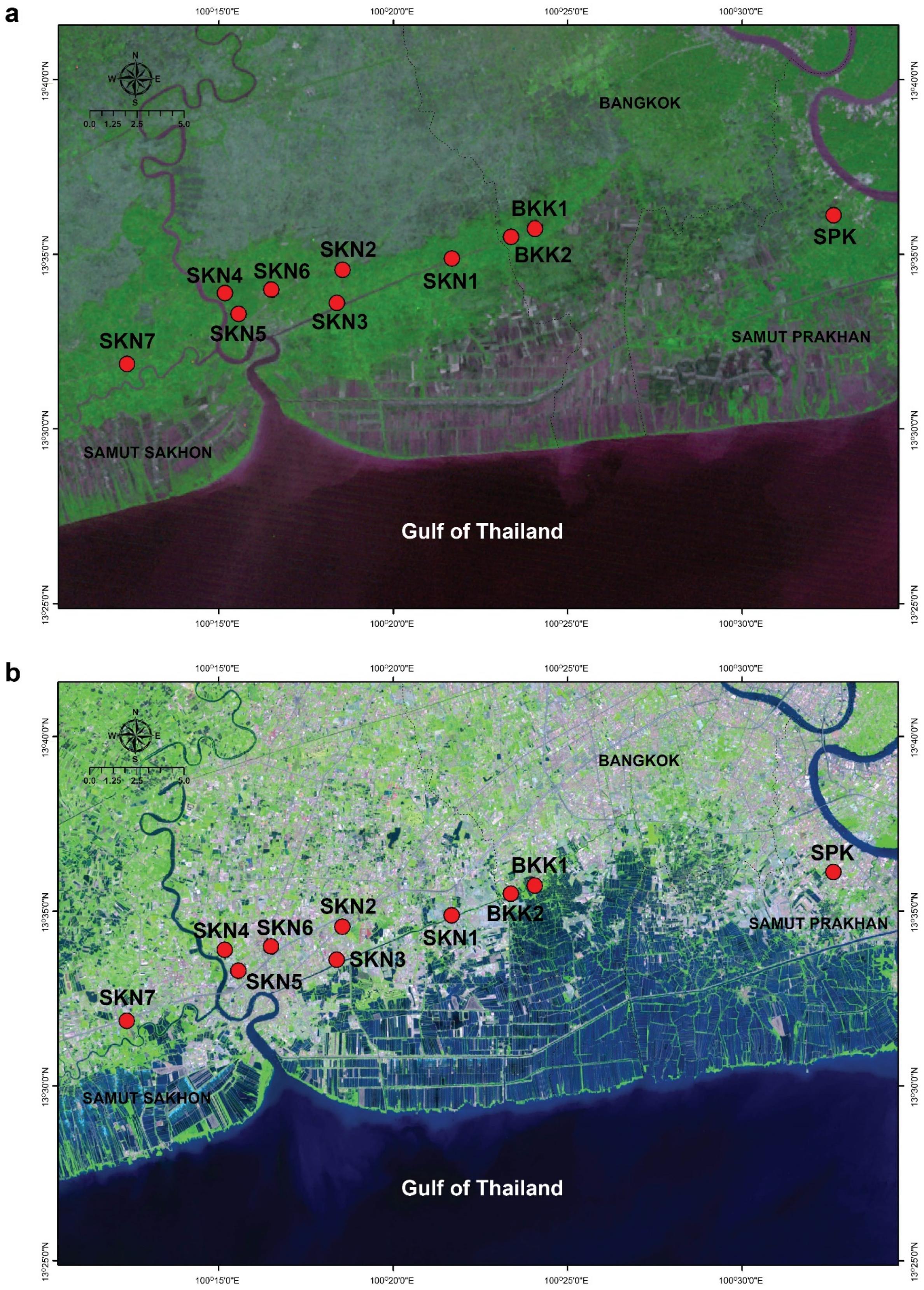
a
100°15'0"E
100°20'0"E
100°25'0"E
100°30'0"E
13°40'0"N
13°35'0"N
13°30'0"N
13°25'0"N
N
W
E
S
0.0 1.25 2.5 5.0
BANGKOK
BKK1
BKK2
SPK
SKN1
SKN2
SKN3
SKN4
SKN5
SKN6
SKN7
SAMUT PRAKHAN
SAMUT SAKHON
Gulf of Thailand
b
BANGKOK
BKK1
BKK2
SPK
SKN1
SKN2
SKN3
SKN4
SKN5
SKN6
SKN7
SAMUT PRAKHAN
SAMUT SAKHON
Gulf of Thailand

Figure S11.
