# Supplementary material for: Saving the Mahachai Betta: Genetic Erosion and Conservation Priorities Under Urbanization Pressure
Source: Animals (Basel). 2025 Sep 26;15(19):2820. doi: 10.3390/ani15192820 (PMC12523270; doi:10.3390/ani15192820)
Supplement: Supplementary file 1 [file animals-15-02820-s001.zip › ★★Supplementary_Information_V5.pdf]

Supplementary Tables

Table S1. Summary of Mahachai Betta (*Betta mahachaiensis*) individuals sampled in this study.

Table S2. Microsatellite primers and sequences used in this study.

Table S3. Mahachai Betta (*Betta mahachaiensis*) occurrence points considering geographic coordinates, genetic diversity, habitat suitability value, and landscape-level variables.

Table S4. Genetic diversity of 81 Mahachai Betta (*Betta mahachaiensis*) individuals based on 13 microsatellite loci.

Table S5. Comparison of genetic diversity parameters between Mahachai Betta (*Betta mahachaiensis*) individuals based on 13 microsatellite loci.

Table S6. Hardy–Weinberg and linkage disequilibrium analysis of the alleles of 13 microsatellites of Mahachai Betta (*Betta mahachaiensis*) individuals from SPK.

Table S7. Hardy–Weinberg and linkage disequilibrium analysis of the alleles of 13 microsatellites of Mahachai Betta (*Betta mahachaiensis*) individuals from BKK1.

Table S8. Hardy–Weinberg and linkage disequilibrium analysis of the alleles of 13 microsatellites of Mahachai Betta (*Betta mahachaiensis*) individuals from BKK2.

Table S9. Hardy–Weinberg and linkage disequilibrium analysis of the alleles of 13 microsatellites of Mahachai Betta (*Betta mahachaiensis*) individuals from SPK1.

Table S10. Hardy–Weinberg and linkage disequilibrium analysis of the alleles of 13 microsatellites of Mahachai Betta (*Betta mahachaiensis*) individuals from SPK2.

Table S11. Hardy–Weinberg and linkage disequilibrium analysis of the alleles of 13 microsatellites of Mahachai Betta (*Betta mahachaiensis*) individuals from SPK3.

Table S12. Hardy–Weinberg and linkage disequilibrium analysis of the alleles of 13 microsatellites of Mahachai Betta (*Betta mahachaiensis*) individuals from SPK4.

Table S13. Hardy–Weinberg and linkage disequilibrium analysis of the alleles of 13 microsatellites of Mahachai Betta (*Betta mahachaiensis*) individuals from SPK5.

Table S14. Hardy–Weinberg and linkage disequilibrium analysis of the alleles of 13 microsatellites of Mahachai Betta (*Betta mahachaiensis*) individuals from SPK6.

Table S15. Hardy–Weinberg and linkage disequilibrium analysis of the alleles of 13 microsatellites of Mahachai Betta (*Betta mahachaiensis*) individuals from SPK7.

Table S16. Pairwise genetic relatedness (r) of all 17 Mahachai Betta (*Betta mahachaiensis*) individuals from SPK.

Table S17. Inbreeding coefficients, relatedness, effective population size, and the ratio of effective population size and census population (N_e/N) of the 81 Mahachai Betta (*Betta mahachaiensis*) individuals.
Table S18. Pairwise genetic relatedness (*r*) of all 11 Mahachai Betta (*Betta mahachaiensis*) individuals from BKK1.
Table S19. Pairwise genetic relatedness (*r*) of all three Mahachai Betta (*Betta mahachaiensis*) individuals from BKK2.
Table S20. Pairwise genetic relatedness (*r*) of all four Mahachai Betta (*Betta mahachaiensis*) individuals from SKN1.
Table S21. Pairwise genetic relatedness (*r*) of all 20 Mahachai Betta (*Betta mahachaiensis*) individuals from SKN2.
Table S22. Pairwise genetic relatedness (*r*) of all five Mahachai Betta (*Betta mahachaiensis*) individuals from SKN3.
Table S23. Pairwise genetic relatedness (*r*) of all 10 Mahachai Betta (*Betta mahachaiensis*) individuals from SKN4.
Table S24. Pairwise genetic relatedness (*r*) of all three Mahachai Betta (*Betta mahachaiensis*) individuals from SKN5.
Table S25. Pairwise genetic relatedness (*r*) of all three Mahachai Betta (*Betta mahachaiensis*) individuals from SKN6.
Table S26. Pairwise genetic relatedness (*r*) of all five Mahachai Betta (*Betta mahachaiensis*) individuals from SKN7.
Table S27. Distributions of *r* and F_{IS} values for Mahachai Betta (*Betta mahachaiensis*).
Table S28. Pairwise inbreeding coefficients (F_{IS}) of all 17 Mahachai Betta (*Betta mahachaiensis*) individuals from SPK.
Table S29. Pairwise inbreeding coefficients (F_{IS}) of all 11 Mahachai Betta (*Betta mahachaiensis*) individuals from BKK1.
Table S30. Pairwise inbreeding coefficients (F_{IS}) of all three Mahachai Betta (*Betta mahachaiensis*) individuals from BKK2.
Table S31. Pairwise inbreeding coefficients (F_{IS}) of all four Mahachai Betta (*Betta mahachaiensis*) individuals from SKN1.
Table S32. Pairwise inbreeding coefficients (F_{IS}) of all 20 Mahachai Betta (*Betta mahachaiensis*) individuals from SKN2.
Table S33. Pairwise inbreeding coefficients (F_{IS}) of all five Mahachai Betta (*Betta mahachaiensis*) individuals from SKN3.

Table S34. Pairwise inbreeding coefficients (F_{IS}) of all 10 Mahachai Betta (*Betta mahachaiensis*) individuals from SKN4.

Table S35. Pairwise inbreeding coefficients (F_{IS}) of all three Mahachai Betta (*Betta mahachaiensis*) individuals from SKN5.

Table S36. Pairwise inbreeding coefficients (F_{IS}) of all three Mahachai Betta (*Betta mahachaiensis*) individuals from SKN6.

Table S37. Pairwise inbreeding coefficients (F_{IS}) of all five Mahachai Betta (*Betta mahachaiensis*) individuals from SKN7.

Table S38. Pairwise genetic differentiation (F_{ST}), pairwise F_{ST}^{ENA} values with ENA correction for null alleles, and R_{ST} values determined using FSTAT version 2.9.3 (Goudet, 1995) of Mahachai Betta (*Betta mahachaiensis*) based on 13 microsatellite loci. The number indicates *p*-values with 110 permutations.

Table S39. Analysis of molecular variance (AMOVA) results for Mahachai Betta (*Betta mahachaiensis*) individuals based on 13 microsatellite loci using Arlequin version 3.5.2.2 (Excoffier & Lischer, 2010).

Table S40. Pairwise population Nei's genetic distance (*D*) values (GenAlEx version 6.5) (Peakall & Smouse, 2012) of Mahachai Betta (*Betta mahachaiensis*) individuals based on 13 microsatellite loci.

Table S41. Wilcoxon signed-rank test to assess mutation drift equilibrium across various models in the 81 samples of Mahachai Betta (*Betta mahachaiensis*).

Table S42. All source/recipient population comparisons with the mean migration rates and 95% confidence intervals determined by BAYESASS using microsatellite data.

Table S43. Bayesian estimates of mutation-scaled effective population sizes (Θ) and asymmetric migration rates (*M*) calculated among the 81 Mahachai Betta (*Betta mahachaiensis*) individuals across 13 microsatellite loci.

Table S44. The effective number of immigrants (N_m) from population *i* to population *j* per generation.

Table S45. Multiple linear regression model of the genetic diversity and habitat suitability of Mahachai Betta (*Betta mahachaiensis*) considering landscape-level variables.

Supplementary Figure

Figure S1. Water quality in the habitats of populations of Mahachai Betta (*Betta mahachaiensis*). (a) pH, (b) dissolved oxygen, (c) conductivity, and (d) salinity.

Figure S2. Simulation results illustrating the intergenerational connections in terms of (a) expected heterozygosity and (b) allelic richness.

Figure S3. (a) Observed distribution of relatedness (*r*) in Mahachai Betta (*Betta mahachaiensis*) populations plotted against expected distributions. (b) Observed distribution of inbreeding coefficients (F_{IS}) in Mahachai Betta (*Betta mahachaiensis*) populations plotted against expected distributions

Figure S4. Mapping of expected heterozygosity (H_e) against inbreeding coefficients (F_{IS}) along the length of the physical map. (a) Mahachai Betta (*Betta mahachaiensis*) populations. (b) microsatellite loci.

Figure S5. Population structure of the 10 Mahachai Betta (*Betta mahachaiensis*) populations, (a) Evanno's Δ*K* and (b) ln P(*K*) plots.

Figure S6. Discriminant analysis of principal components (DAPC) for Mahachai Betta (*Betta mahachaiensis*) based on 13 microsatellite loci.

Figure S7. Loci bias in Mahachai Betta (*Betta mahachaiensis*) populations.

Figure S8. (a) Historical gene flow dynamics based on MIGRATE-N among *Betta mahachaiensis* populations over space (only values ≥500 are shown). (b) Recent gene flow dynamics based on BayesAss among *B. mahachaiensis* populations (only values ≥0.05 are shown). The width of the curves represents the relative magnitude of migration.

Figure S9. Relationships between genetic diversity and habitat suitability in Mahachai Betta (*Betta mahachaiensis*). (a) Allelic richness ($\rho = -0.042, p = 0.918$). (b) Expected heterozygosity ($\rho = 0.127, p = 0.732$). (c) Inbreeding coefficients (F_{IS}) ($\rho = 0.030, p = 0.945$).

Figure S10. Land-use types in 2002 (a) and 2019 (b) in the study area. Source: Land Development Department (2021).

Figure S11. Combination bands 7, 6, and 5 from Landsat 1 view on January 7, 1973 (a) and February 14, 2024 (b). In this false-color image, shades of green indicate vegetated land.
